# Fresh and Fermented Beetroot: From Microbial Biotransformation to Nitrate–Nitrite–NO Signalling, Redox Homeostasis and Microbiome Interactions

**DOI:** 10.3390/ijms27198629

**Published:** 2026-09-26

**Authors:** Halina Tkaczenko, Anna Rymuszka, Renata Kołodziejska, Natalia Kurhaluk

**Affiliations:** 1 Institute of Biology, Pomeranian University in Słupsk, Arciszewski St. 22B, 76-200 Słupsk, Poland; 2 Department of Physiology and Toxicology, Faculty of Medicine, The John Paul II Catholic University of Lublin, Konstantynów St. 1I, 20-708 Lublin, Poland; anna.rymuszka@kul.pl; 3 Department of Medical Biology and Biochemistry, Collegium Medicum in Bydgoszcz, Nicolaus Copernicus University in Toruń, M. Karłowicz St. 24, 85-092 Bydgoszcz, Poland; renatak@cm.umk.pl

**Keywords:** dietary nitrate, betalains, gut microbiota, functional foods, nitrate–nitrite–NO metabolism, cardiovascular health

## Abstract

Beetroot (*Beta vulgaris* L.) is a rich source of dietary nitrates, polyphenols, and other bioactive compounds that have potential cardiovascular, metabolic, and health-promoting effects. While previous reviews have largely focused on the nitrate-derived nitric oxide (NO) signalling pathway or the antioxidant properties of the pigments known as ‘betalains’, there is an increasing body of evidence that suggests microbial transformation and fermentation can substantially modify the biological properties of beetroot. This narrative review summarises the latest research on beetroot phytochemistry, nitrate–nitrite–NO metabolism, microbial biotransformation, fermentation and host physiological responses. Particular attention is given to the impact of fermentation on phenolic compounds, betalains, and the metabolism of nitrates and nitrites, as well as to its effects on antioxidant activity, bioaccessibility, and interactions with the gut microbiome. The available evidence suggests that fermentation can alter the beetroot matrix, increasing the accessibility or recovery of certain bioactive compounds while producing additional microbial metabolites with potential biological activity. These changes may complement the well-established effects of beetroot-derived nitrates and betalains on vascular function, redox homeostasis, inflammation and exercise-related physiology. However, evidence specifically linking fermented beetroot to clinical outcomes is limited, and the effects of fermentation depend heavily on microbial strains, processing conditions, the food matrix and host characteristics. Interindividual differences in microbiome composition may further contribute to variability in responses to beetroot-derived compounds. Thus, fermentation is a promising strategy for increasing the functional potential of beetroot by altering the bioaccessibility of its bioactive constituents through microbial biotransformation. Further well-designed human studies are needed to establish whether these compositional and mechanistic changes result in clinically significant benefits, and to clarify how individual microbiome profiles contribute to responses to fermented beetroot.

## 1. Introduction

Beetroot (*Beta vulgaris* L.) is an important cultivated crop and widely consumed vegetable, with a nutritional and functional relevance that extends beyond its traditional use as a food ingredient. Red beetroot (Conditiva Group) is a particularly good source of dietary fibre, minerals, vitamins, nitrogen-containing compounds, phenolic compounds, and relatively high levels of inorganic nitrate [1,2]. Nitrate is of particular interest due to its involvement in the nitrate–nitrite–nitric oxide pathway, while phenolic compounds and betalains contribute to beetroot’s antioxidant and redox-related properties [1,3,4]. However, the biological potential of beetroot cannot be attributed to individual compounds alone. The composition of beetroot and the stability, release and bioaccessibility of its constituents depend on factors such as genotype, cultivar, cultivation conditions, maturity, storage and processing, as well as interactions within the food matrix [1,5,6,7,8]. Therefore, beetroot should be considered a chemically dynamic matrix, where the potential physiological effects of bioactive constituents depend on their stability, bioaccessibility, metabolism, and gastrointestinal fate, as well as their presence and concentration.

These compositional features are directly relevant to the physiological effects of beetroot, particularly nitrate-rich products, which have been extensively studied in relation to vascular function, blood pressure and exercise performance. Dietary nitrate contributes to the formation of nitric oxide (NO) and vascular regulation via the enterosalivary pathway. In this pathway, oral nitrate-reducing bacteria convert nitrate to nitrite, thereby emphasising the significance of the oral microbiome in systemic NO signalling [9,10,11,12]. Studies in humans suggest that nitrate-rich beetroot products may reduce blood pressure and improve certain aspects of exercise performance; however, these effects vary depending on dose, exercise modality, population characteristics and other individual factors [11,13,14,15,16,17,18,19]. Consequently, nitrate concentration alone is insufficient to predict physiological effects, which also depend on the characteristics of the food matrix, the capacity of the oral microbiome to reduce nitrate, and subsequent nitrate and nitrite metabolism. Processing can further influence these relationships by modifying the structure of the food matrix, the stability of phytochemicals, their chemical form and their bioaccessibility [7,8]. Thermal treatment, freezing and other technological processes may affect beetroot pigments and phenolic compounds, as well as their gastrointestinal release. Betalains are particularly sensitive to temperature, pH, oxygen and light [1,3,20,21,22]. Therefore, changes in compound concentrations or antioxidant activity following processing do not necessarily indicate improved nutritional or physiological efficacy, since the biological relevance of a compound depends on its abundance, release from the matrix, stability, gastrointestinal transformation, bioaccessibility and subsequent absorption or metabolism [8,23].

Fermentation is of particular interest among the processing approaches capable of modifying this matrix because it combines physicochemical changes in the plant material with microbial metabolism. Spontaneous and controlled lactic acid fermentation of beetroot have both been investigated, but the resulting changes depend strongly on the microorganisms involved, as well as on the conditions of the process, including fermentation time and temperature, the characteristics of the substrate, salt concentration, oxygen availability, particle size, the starter culture used, the formulation, post-fermentation processing and storage [24,25,26,27,28,29,30]. Fermentation can alter the pH level, the concentration of organic acids and phenolic compounds, the levels of nitrate and nitrite, and the stability, release and accessibility of phytochemicals [31,32]. Meanwhile, microbial activity can generate or release additional metabolites, such as free amino acids and other transformation products [23,24,26]. Consequently, fermented beetroot cannot be considered a standardised or chemically uniform product, and compositional changes must be interpreted in the context of the specific raw material, microbial strain or consortium, and processing conditions [26,27,30]. It is important to note that an increase or decrease in the measured concentration or extractability of a compound does not necessarily indicate a corresponding change in its bioaccessibility, systemic bioavailability, or physiological effect [8,23].

Although the effects of nitrate-rich, unfermented beetroot products on blood pressure and exercise performance have been extensively investigated, less is known about whether fermentation alters the biological relevance of these compounds or their subsequent physiological effects [11,13,14,15,16,17,18,19,33,34,35,36]. In particular, there is limited direct evidence establishing whether fermentation enhances systemic nitrate bioavailability, modifies nitrate-to-nitrite conversion or produces greater NO-mediated effects than appropriately matched unfermented beetroot products [28,37,38,39]. The oral microbiome is an important determinant of this pathway, as demonstrated by the effects of antibacterial mouthwash on nitrate reduction and some physiological responses to dietary nitrate [40,41,42,43,44]. Similarly, experimental studies indicate that fermentation can modify phytochemical profiles, their gastrointestinal recovery and their interactions with the intestinal microbiome. However, these observations do not demonstrate improved systemic bioavailability or human health outcomes by themselves [23,27,31,32,45]. Furthermore, fermentation-induced changes are not necessarily linear or uniformly beneficial; effects on phenolic concentration and bioaccessibility can vary depending on the beetroot matrix and processing conditions [46]. These findings highlight a central knowledge gap: although the composition, processing and fermentation of beetroot have been reviewed, it remains unclear to what extent fermentation-induced compositional changes translate into altered bioaccessibility, metabolism, microbiome interactions and biological effects [23,37].

Against this background, the present review critically examines how fermentation modifies beetroot, and to what extent these changes may affect its functional properties. It does not assume that fermented beetroot is inherently superior to fresh or conventionally processed products. It focuses on the metabolism of nitrates and nitrites, the nitrate–nitrite–NO pathway, and phenolic compounds and betalain pigments, including betacyanins and betaxanthins, as well as fermentation-derived metabolites and their interactions with the oral and gastrointestinal microbiome. It also considers the effects on redox, inflammation, the cardiovascular system, metabolism, exercise, healthy ageing, product safety and process standardisation. Particular emphasis is placed on distinguishing evidence obtained directly from fermented beetroot from findings derived from fresh or conventionally processed beetroot, isolated nitrate, or individual beetroot constituents. The review also aims to separate compositional changes from bioaccessibility, mechanistic effects, and demonstrated human health outcomes [37]. By integrating these levels of evidence, the review aims to identify the conditions under which fermentation may modify the functional potential of beetroot, and highlight key uncertainties that require comparative, pharmacokinetic, experimental and adequately powered human clinical studies.

## 2. Review Methodology

### 2.1. Literature Search Strategy

A narrative literature review was conducted to critically evaluate the available evidence on the composition, processing, fermentation, bioaccessibility, biological activity and potential health effects of beetroot (*Beta vulgaris* L.), particularly in relation to fermented products. Searches were performed in the databases PubMed, Scopus and Web of Science, covering literature from the fields of biomedicine, nutrition, food science, microbiology and interdisciplinarity. Publications from 2006 to June 2026 were considered. This timeframe was chosen to capture the most recent evidence on beetroot phytochemistry, nitrate–nitrite–nitric oxide metabolism, processing, fermentation, microbial biotransformation, bioaccessibility and functional food applications. Earlier publications were also included if they provided essential methodological or mechanistic information that was not adequately represented in more recent literature. Search terms included “beetroot” OR “Beta vulgaris”, “fermented beetroot” OR “beetroot fermentation”, “nitrate”, “nitrite”, “nitric oxide”, “betalain” OR “betanin”, “polyphenol”, “phenolic compounds”, “bioaccessibility”, “bioavailability”, “gut microbiota”, “microbial fermentation”, “food processing”, “antioxidant”, “anti-inflammatory”, “cardiovascular”, “exercise”, “ageing”, and “functional food”. Reference lists of key articles and reviews were also screened to identify additional relevant publications. Only publications available in English were considered for literature selection.

### 2.2. Eligibility and Selection of Evidence

Eligible evidence included peer-reviewed original research, clinical studies, human intervention studies, experimental studies, systematic reviews, meta-analyses and relevant food processing and fermentation studies that addressed one or more of the following domains: (i) beetroot composition and phytochemistry, (ii) nitrate and nitrite metabolism, (iii) betalains and phenolic compounds, (iv) fermentation and microbial biotransformation, (v) processing-related changes in chemical composition and matrix properties, (vi) bioaccessibility and bioavailability, (vii) interactions with oral or gastrointestinal microbiota, (viii) cardiovascular, metabolic, inflammatory, exercise-related or ageing-associated outcomes, and (ix) food safety, stability and technological applications.

As the review focused primarily on fermented beetroot, particular emphasis was placed on studies that directly investigated fermented products or compared fermented and non-fermented preparations. Evidence from fresh or conventionally processed beetroot, isolated nitrates or nitrites, purified betalains and other fermented foods was mainly used to support mechanistic interpretation and contextualise findings, rather than as equivalent evidence for fermented beetroot. Studies employing chromatographic or mass spectrometric profiling, metabolomics, simulated gastrointestinal digestion, microbial profiling and controlled fermentation models were deemed particularly informative in characterising compositional changes and potential mechanisms.

### 2.3. Evidence Synthesis and Critical Appraisal

As this was a narrative review, the evidence was synthesised qualitatively rather than quantitatively. The findings were categorised according to the preparation of the beetroot, the fermentation conditions, the constituents investigated, the experimental model and the biological outcome. Particular attention was paid to the differences between chemical and in vitro assays, simulated digestion, animal studies, observational studies, clinical trials and systematic reviews or meta-analyses. The strength of the evidence was evaluated based on whether fermentation-related compositional changes could reasonably be linked to biological effects. Changes in nitrate, nitrite, betalains, phenolic compounds, antioxidant assay values or microbial metabolites were distinguished from evidence of gastrointestinal bioaccessibility, systemic pharmacokinetics and clinically relevant physiological outcomes. This distinction was employed to prevent the interpretation of chemical or in vitro changes as direct evidence of improved health effects in humans.

The synthesis also considered heterogeneity in fermented beetroot products, including cultivar and raw material, microbial strains or consortia, fermentation time and temperature, salt concentration, pH, particle size, post-fermentation processing, storage and pasteurisation. This enabled the assessment of whether the reported effects were reproducible across products or dependent on specific processing conditions. Studies addressing fermentation-related safety issues, such as microbiological stability and the potential formation of undesirable metabolites, were also considered.

## 3. Composition of Beetroot Before Fermentation

Fresh beetroot (*Beta vulgaris* L.) is a chemically complex plant matrix containing inorganic nitrates, betalain pigments, phenolic compounds, carbohydrates, dietary fibre, nitrogen-containing compounds, vitamins, minerals and a variety of other phytochemicals [1,47,48,49]. The relative abundance and chemical profile of these constituents are not fixed but may vary considerably among cultivars. They are also influenced by genotype, agronomic and environmental conditions, plant maturity, and post-harvest handling and storage [1,47,50]. This variability in composition is particularly relevant in the context of fermentation, as the biochemical characteristics of the raw material can affect the extent of microbial transformation, as well as the composition and functional properties of the final fermented product [1,8,47].

Importantly, beetroot should not simply be regarded as a source of individual bioactive compounds. Unlike isolated nitrates or purified betalains, whole beetroot provides these constituents within a complex food matrix comprising dietary fibre, cell wall components, phenolic compounds, pigments and other constituents. The stability, release and accessibility of these constituents during digestion may be affected by interactions within this matrix [8,46,51]. Consequently, the concentration of a given compound measured in fresh beetroot does not necessarily predict its bioaccessibility or biological relevance after consumption [52]. This distinction is particularly important for phenolic compounds and betalains, as their stability and release from the plant matrix can be significantly impacted by gastrointestinal conditions, food processing and interactions with other matrix components [49,52].

Processing may modify these relationships further by altering the structure of plant tissue and changing the physicochemical environment in which beetroot constituents are present. Disruption of cellular and cell wall structures may facilitate the release of matrix-bound or entrapped compounds. However, processing-induced degradation or chemical transformation may simultaneously reduce the concentration of other bioactive constituents [8,49,53]. Therefore, processing cannot be considered solely in terms of nutrient retention or loss, as it may also alter the balance between compound stability, release and bioaccessibility [8,49,53]. Fermentation is particularly relevant in this context, as microbial metabolism can modify the beetroot matrix itself while simultaneously transforming selected chemical constituents and generating additional metabolites [8,46].

For these reasons, the biochemical composition of fresh beetroot is the essential reference point against which fermentation-induced changes should be evaluated. A meaningful assessment of fermented beetroot requires consideration of both absolute changes in the concentrations of nitrates, betalains, phenolic compounds and other constituents and changes in their chemical forms, interactions with the food matrix and potential accessibility within the gastrointestinal tract [8,46,51]. This matrix-oriented approach is important for understanding how fermentation can affect the functional properties of beetroot, ultimately influencing nitrate–nitrite–NO signalling, redox homeostasis and microbiome-mediated cellular responses [8,46].

### 3.1. Nitrate-, Nitrite- and Nitrogen-Containing Compounds

Beetroot is one of the most widely investigated dietary sources of inorganic nitrate (NO_3_^−^), and beetroot juice remains the most commonly used formulation in nitrate supplementation studies [54,55]. However, the nitrate content of beetroot products is not uniform and depends strongly on the formulation and processing method. For example, when nitrate concentrations were expressed per 100 g or 100 mL of product, beetroot-cereal bars contained approximately 14.0 mmol NO_3_^−^, compared to 6.30 mmol in beetroot gel, 6.90 mmol in beetroot chips, and 4.10 mmol in beetroot juice [56]. These differences are important when translating experimental doses into realistic dietary portions, because the physiological effects of beetroot supplementation depend not only on the presence of nitrate but also on the amount delivered and the form in which it is consumed.

In practical terms, relatively large portions of some beetroot formulations may be required to achieve nitrate doses commonly used in intervention studies. A portion of approximately 200 mL of beetroot juice or 100 g of beetroot gel or chips can provide more than 6.3 mmol of dietary nitrate [56]. Although such doses can be effective, gastrointestinal discomfort and beeturia have been reported in some individuals and may influence the acceptability of prolonged supplementation [48]. This issue is particularly relevant for fermented beetroot, because fermentation may offer an alternative food matrix in which nitrate-rich beetroot can be incorporated into a more convenient and potentially better-tolerated dietary product [37]. Importantly, however, the suitability of fermented beetroot as a nitrate-delivery system should be evaluated on the basis of its actual nitrate content, stability during fermentation and storage, and the extent to which the nitrate remains available for subsequent conversion to nitrite and nitric oxide.

The biological significance of dietary nitrate is closely related to its ability to enter the nitrate–nitrite–NO pathway, which complements the classical nitric oxide synthase (NOS)-dependent pathway. Under physiological conditions, NO is produced predominantly through the oxidation of L-arginine by NOS enzymes, including endothelial NOS (eNOS), neuronal NOS (nNOS), and inducible NOS (iNOS), with eNOS-derived NO playing a central role in vascular homeostasis [57,58]. However, NOS-dependent NO synthesis requires molecular oxygen and can become less efficient under conditions such as hypoxia or reduced endothelial NOS activity. Dietary nitrate provides an alternative, oxygen-independent route for NO generation and therefore represents an important metabolic link between diet, the microbiome, and vascular signalling [40,57,59].

Following ingestion, dietary nitrate is rapidly absorbed in the upper gastrointestinal tract and enters the systemic circulation. Approximately one quarter of circulating nitrate is actively taken up by the salivary glands and secreted back into the oral cavity, where its concentration in saliva can considerably exceed that in plasma [40,59]. This process, known as enterosalivary nitrate circulation, creates an important interface between dietary nitrate and the oral microbiome. Nitrate-reducing bacteria, which reside predominantly on the tongue and other oral surfaces, express nitrate reductases that convert nitrate (NO_3_^−^) to nitrite (NO_2_^−^). Genera including *Neisseria*, *Rothia*, *Veillonella*, *Actinomyces*, and related taxa have been implicated in this process [57,59,60]. Therefore, the oral microbiome is an active participant in the bioactivation of dietary nitrate, not merely a passive component of the digestive tract.

Once swallowed, nitrite can follow several metabolic routes. In the acidic environment of the stomach, nitrite is protonated to nitrous acid, which can subsequently decompose to NO and other reactive nitrogen species [40,59]. A proportion of nitrite also reaches the systemic circulation, where it can serve as a circulating reservoir for NO. Under conditions of reduced oxygen availability or low pH, nitrite can be reduced to NO by several enzymatic and non-enzymatic mechanisms involving deoxyhemoglobin, deoxymyoglobin, xanthine oxidoreductase, and components of the mitochondrial respiratory chain [59,61,62]. The importance of this pathway therefore extends beyond simple nitrate supplementation: it provides a mechanism through which dietary nitrate can contribute to NO signalling precisely under physiological conditions in which oxygen-dependent NO synthesis may be limited.

The resulting NO participates in a well-characterised signalling cascade in vascular smooth muscle. NO diffuses from the endothelium into adjacent smooth muscle cells, where it activates soluble guanylate cyclase (sGC), increasing intracellular cyclic guanosine monophosphate (cGMP). Activation of the sGC-cGMP pathway promotes calcium-dependent smooth muscle relaxation and vasodilation [63,64]. Through these mechanisms, increased NO availability can support endothelial function, vascular compliance, microvascular perfusion, and tissue oxygen delivery, while dietary nitrate supplementation has been associated with reductions in blood pressure and improvements in vascular function in selected populations [14,38,65].

The biological effects of NO are not restricted to vasodilation. NO also participates in the regulation of platelet activity, vascular inflammation, endothelial integrity, and redox signalling [63,66]. However, these effects are strongly dependent on NO concentration, cellular source, and biological context; therefore, NO should not be considered uniformly anti-inflammatory. Physiological NO generated by eNOS generally supports vascular homeostasis, whereas excessive NO production, particularly in conjunction with superoxide, may contribute to reactive nitrogen species formation and nitrosative stress [66,67]. This distinction is important when considering beetroot-derived nitrate, because the potential cardiovascular benefits of enhanced NO bioavailability must be interpreted within the broader redox environment of the cell.

This is where the phytochemical composition of beetroot becomes particularly relevant. Beetroot is not simply a nitrate-containing food but a complex source of betalains, phenolic compounds, betaine, and other bioactive constituents that may act through pathways complementary to nitrate-derived NO signalling [55,68]. Betalains, including betanin and betaxanthins such as vulgaxanthin, possess direct radical-scavenging activity and can modulate endogenous antioxidant defence mechanisms [68,69,70]. Experimental evidence indicates that betanin can promote Nrf2 activation and its interaction with antioxidant response elements (AREs), thereby increasing the expression of antioxidant and phase II detoxification enzymes [68,69]. Consequently, the antioxidant activity of beetroot may involve not only direct neutralisation of reactive species but also stimulation of endogenous cellular defence systems.

The interaction between betalains and inflammatory signalling provides another potential layer of protection. Experimental studies have shown that beetroot betalains can modulate NF-κB activity and reduce the expression or production of inflammatory mediators, including TNF-α and IL-6, while also affecting inflammatory enzymes such as cyclooxygenase and lipoxygenase [68,70,71]. These observations suggest that betalains and other beetroot polyphenols may help to create a cellular redox environment that is more favourable for maintaining NO bioavailability. Because superoxide can rapidly react with NO to form peroxynitrite, limiting excessive oxidative stress may indirectly preserve NO signalling and reduce the shift from physiological NO signalling toward nitrosative stress [67,72].

These mechanisms suggest that the effects of beetroot supplementation are more widespread than previously thought. Rather than acting solely as a source of nitrate, beetroot can influence nitrate–nitrite–NO metabolism, vascular signalling, oxidative stress and inflammatory pathways by combining the effects of its constituent compounds [55,68]. This multi-component activity is particularly relevant to fermented beetroot, where microbial metabolism can further modify the chemical composition, bioaccessibility and biological activity of nitrates, betacyanins, phenolic compounds and other matrix-associated substances. Fermentation can therefore introduce an extra dimension to the nitrate–nitrite–NO axis by altering the food matrix and potentially interacting with the microbial communities responsible for nitrate reduction. Accordingly, fermented beetroot should not simply be considered an alternative nitrate supplement, but rather a dynamic food matrix that links dietary nitrate metabolism with redox regulation, inflammatory signalling and microbiome-mediated cellular responses.

### 3.2. Betalains

Alongside inorganic nitrates, the group of bioactive compounds known as “betalains” is one of the most characteristic of red beetroot, and they may contribute substantially to its antioxidant, anti-inflammatory and cytoprotective properties. These water-soluble, nitrogen-containing pigments are synthesised through a tyrosine-derived pathway and are characteristic of plants belonging to the order Caryophyllales [69,73,74]. According to their chemical structure and colour, they are divided into two major subclasses: red-violet betacyanins and yellow-orange betaxanthins. Betacyanins include betanin, isobetanin, betanidin and related compounds, while betaxanthins comprise pigments such as vulgaxanthin I and II, and indicaxanthin [69,73,74]. Betanin is generally the predominant betalain in red beetroot and is considered one of the main contributors to its characteristic red-violet colour [69,74].

The profile of betalain pigments in beetroot is not constant. It varies depending on the cultivar, the maturity of the plant, the growing conditions, environmental factors and post-harvest handling. Exposure to elevated temperatures, light, oxygen and an unfavourable pH can accelerate pigment degradation [69,74,75]. Consequently, the amount of betalains in fresh beetroot cannot be assumed to remain unchanged during processing or fermentation. This is particularly relevant for fermented beetroot because acidification, microbial metabolism, matrix disruption and storage can all alter the concentration and relative distribution of individual betalains. Indeed, experimental studies of spontaneous beetroot fermentation have shown substantial changes in the profile of the pigments, with reductions in total content accompanied by the formation or relative enrichment of individual derivatives [25]. More recent studies using selected lactic acid bacteria have likewise demonstrated that fermentation can promote the formation of compounds such as isobetanin and neobetanin, modifying the polyphenolic profile of beetroot juice [27]. Therefore, fermentation should not simply be viewed as a process that preserves or decreases the amount of betalains present, but rather as a biochemical transformation that can change the composition of the pigment fraction.

This distinction is important when considering the functional properties of fermented beetroot. Betanin and related betalains have a significant chemical antioxidant capacity, which is partly due to their ability to donate electrons or hydrogen atoms and interact with reactive species [69,76]. In vitro studies have demonstrated that betanin retains considerable antioxidant activity during simulated gastrointestinal digestion, though its recovery decreases substantially during subsequent colonic fermentation [77,78]. These findings highlight a significant limitation of conventional antioxidant measurements: the antioxidant capacity of a food or an in vitro digestive model does not necessarily equate to systemic exposure or biological activity in humans. Therefore, the relevant question is not only how much betanin is present in fermented beetroot, but also which of the derived compounds remain chemically stable and become bioaccessible during digestion to be available for interaction with intestinal or systemic targets.

Further support for this cautious interpretation comes from human pharmacokinetic studies. Following the consumption of beetroot juice or whole beetroot, intact betalains can be detected in the urine; however, only a small proportion of the administered dose is recovered as the original compounds [68,79]. One human study found that approximately 0.28% of the administered dose of betanin and isobetanin was recovered in urine as intact compounds, with urinary excretion peaking at around 3 h after ingestion [79]. Another controlled study found that betanin was not detectable in plasma following the consumption of 250 mL of beetroot juice or 300 g of whole beetroot, despite the products having a relatively high betanin content [80]. These observations suggest that the biological effects of beetroot betalains cannot be attributed solely to sustained high circulating concentrations of intact betanin. Alternative explanations include rapid tissue uptake, extensive metabolism below current detection limits, transformation by intestinal microbiota and elimination through non-renal pathways [22,48,68].

The antioxidant activity of betanin and related compounds appears to involve both direct and indirect mechanisms (Figure 1). As well as scavenging reactive oxygen species (ROS), betanin and related compounds have been reported to modulate endogenous antioxidant defence pathways, particularly the Nrf2-ARE system [68,69,70,76]. Experimental studies indicate that betanins can promote Nrf2 nuclear accumulation and enhance Nrf2-ARE signalling, resulting in increased expression of genes that encode antioxidant and phase II detoxification enzymes [68,69,70]. The distinction between direct radical scavenging and the activation of endogenous antioxidant defences is important because the latter mechanism may persist even when circulating concentrations of intact betalains are relatively low. However, the precise molecular targets responsible for Nrf2 activation by betanin in vivo are not fully understood, and most evidence has been generated in experimental models.

The redox effects of betalains may also be relevant to vascular protection. Excessive oxidative stress can reduce the bioavailability of NO by promoting its reaction with superoxide to form peroxynitrite, thereby shifting NO signalling towards a state of oxidative and nitrosative stress [67]. By limiting these reactions, beetroot betalains may therefore indirectly support the biological activity of nitrate-derived NO. This provides a plausible complementarity between the two major bioactive fractions of beetroot: nitrates contribute to NO generation, while the presence of antioxidants such as betalains may help maintain a cellular redox environment favourable for NO signalling [56,68]. Nevertheless, such an interaction should be regarded as a working hypothesis rather than a demonstrated synergistic effect in humans.

In addition to their antioxidant properties, studies have shown that betalains exhibit anti-inflammatory activity in experimental models. Betalain-rich preparations have been reported to suppress NF-κB-dependent signalling and reduce the expression of inflammatory mediators, including TNF-α and IL-6 [22]. Betanin and related compounds may also modulate enzymes involved in arachidonic acid metabolism, such as cyclooxygenase and lipoxygenase [81]. Further experimental evidence suggests an interaction between Nrf2 and NF-κB signalling, which could provide a molecular basis for simultaneously enhancing antioxidant defences and attenuating inflammatory responses [56,68,82]. However, evidence from human intervention studies is considerably more limited than the extensive in vitro and animal literature. Effects observed with betanin-rich extracts cannot automatically be attributed to purified betanin or fermented beetroot as a whole.

Clinical observations provide preliminary evidence of the cardiovascular and anti-inflammatory effects of beetroot preparations that are rich in betalain. In individuals with coronary artery disease, short-term supplementation with a beetroot extract rich in betalains was associated with favourable changes in selected lipid parameters and blood pressure [83,84]. However, these findings should be interpreted with caution, as the preparations used in human studies contain multiple compounds, and the independent contribution of the different constituents, including nitrate and other beetroot compounds, remains difficult to establish.

From a food technology perspective, betanin is relevant due to its established use as a natural food colouring agent (E162) and its ability to delay lipid oxidation in certain food products [68,69]. However, the same chemical properties that make betanin biologically interesting also present technological challenges. They are sensitive to temperature, light, oxygen, pH and other processing conditions [68,69,75]. Fermentation is therefore a particularly interesting processing strategy, as the gradual reduction in pH and the activity of lactic acid bacteria can influence pigment stability, matrix disintegration, microbial transformation and the formation of secondary metabolites simultaneously. Recent evidence indicates that fermentation can substantially modify the composition of betanin rather than simply preserving its original profile [25,27]. This underlines the importance of characterising the chemistry of fermented beetroot products rather than extrapolating their composition from fresh beetroot or beetroot juice.

Thus, the available evidence suggests that beetroot’s biologically relevant component, betalains, complement nitrate-dependent NO signalling through antioxidant and anti-inflammatory mechanisms. Their direct radical-scavenging activity, potential modulation of the Nrf2-ARE and NF-κB pathways, and interactions with food and the microbial matrix offer several potential mechanisms by which beetroot could influence cellular redox homeostasis and vascular protection [68,69,76]. However, in fermented beetroot, these effects should be considered within a dynamic matrix in which fermentation may alter the composition and bioaccessibility of betalains, as well as microbial metabolism and interactions with other phytochemicals. This is central to understanding fermented beetroot as a functional food that acts via multiple pathways, rather than simply as a vehicle for nitrates or a source of isolated betalains.

### 3.3. Polyphenols, Vitamins, Minerals and Other Constituents

Although nitrates and betalains are the best-known bioactive constituents of beetroot, the biological activity of *B. vulgaris* cannot be explained by these two groups of compounds alone. Beetroot provides a complex matrix of phytochemicals and nutrients that includes phenolic acids, flavonoids, organic acids, vitamins, minerals, amino acids, betaine, saponins and other secondary metabolites. The composition and relative abundance of these constituents vary substantially depending on the cultivar, plant tissue, agronomic conditions, maturity, storage and processing. Fermentation can further modify this profile through microbial metabolism, the release of bound compounds and the formation of new metabolites [7,56,68]. This broader compositional perspective is particularly relevant because several non-nitrate constituents may interact indirectly with nitrate–nitrite–NO signalling. These compounds may help preserve NO bioavailability and complement the vascular and cytoprotective effects associated with the nitrate–nitrite–NO pathway by limiting oxidative reactions, chelating redox-active metals, modulating endogenous antioxidant defence systems, or influencing microbial metabolism [56,68].

Phenolic compounds are another key contributor to the antioxidant potential of beetroot. A variety of phenolic acids have been identified in various beetroot cultivars and preparations, such as gallic acid, caffeic acid, ferulic acid, p-coumaric acid, chlorogenic acid, syringic acid, vanillic acid and p-hydroxybenzoic acid. The concentrations of these acids are influenced by the plant tissue analysed, as well as by cultivation and processing conditions. Generally, the peel contains higher concentrations of several phenolic compounds than the inner root tissue [7,56,68].

The biological relevance of beetroot phenolic compounds lies in their ability to react with reactive oxygen species and influence redox-sensitive and inflammatory signalling pathways. For example, ferulic and caffeic acids have been extensively investigated for their antioxidant and anti-inflammatory activities [85,86], while chlorogenic acid has been linked to the regulation of oxidative stress, inflammation, glucose homeostasis and vascular function [87]. Experimental studies indicate that chlorogenic acid can activate cytoprotective pathways involving the transcription factor Nrf2 and ARE-dependent gene expression. This includes the induction of haem oxygenase-1 (HO-1) and NQO1, as well as enzymes involved in glutathione synthesis. These mechanisms are of particular interest as they may influence the cellular redox environment in which NO signalling operates [87].

Gallic acid is frequently reported as one of the main phenolic constituents of beetroot-derived products. There is experimental and clinical evidence suggesting that gallic acid has potential effects on oxidative stress and inflammatory processes. However, its biological activity is strongly influenced by dose, metabolism, and interactions with other dietary components [88]. Similarly, caffeic acid has pronounced antioxidant properties related to its catechol structure and ability to limit the oxidative modification of biomolecules [89]. However, most data on individual phenolic acids originates from studies using isolated compounds at concentrations that may not be achieved by consuming beetroot or fermented beetroot products. For this reason, the contribution of individual phenolic acids should be interpreted in the context of the whole food.

Beetroot also contains several flavonoids. Reported compounds in *B. vulgaris* include betavulgarin, betagarin, cochliolin A and dihydroisorhamnetin, as well as additional flavonoid derivatives identified in phytochemical analyses [7,90]. More broadly, flavonoids may contribute to antioxidant and cytoprotective effects through radical scavenging, metal chelation and the modulation of redox-sensitive and inflammatory pathways. However, their biological activity depends strongly on their chemical structure, intestinal absorption, microbial biotransformation and subsequent metabolism [91]. This is particularly important in the context of fermented foods, where microbial activity can alter the availability and metabolic fate of phenolic compounds.

Although often less discussed, organic acids are an important component of the beetroot phytochemical matrix. Malic, citric, ascorbic, fumaric and oxalic acids have been identified in beetroot and beetroot-derived products, and depending on the formulation, succinic acid may also be present. Their concentrations can vary considerably between fresh roots, juices, cooked products, powders, chips and more complex formulations, reflecting differences in processing and the original plant material [56,92]. In plants, organic acids contribute to nutrient acquisition, metal chelation and adaptation to environmental stress [93]. In the human diet, they may also influence the redox properties and physicochemical characteristics of the food matrix [52].

Malic and citric acids are generally among the most abundant organic acids reported in beetroot-derived products [56,92]. In addition to their metabolic roles, these acids may contribute to antioxidant protection by interacting with metal ions and limiting metal-catalysed oxidative reactions [94]. Citric acid, in particular, is an effective chelating compound and may act synergistically with other antioxidants present in plant foods [95]. Therefore, organic acids may contribute to the overall redox environment in which nitrate, betalains and phenolic compounds exert their biological effects.

The presence of ascorbic acid is particularly relevant in the context of beetroot. Ascorbic acid, also known as vitamin C, is a well-established water-soluble antioxidant and an important cofactor for several enzymatic reactions. Through its electron-donating capacity, ascorbic acid can protect biomolecules against oxidative damage and participate in the regeneration of other antioxidant systems. It is also necessary for the activity of prolyl and lysyl hydroxylases, which are involved in collagen maturation. This contributes to maintaining normal connective tissue and vascular structure [96]. From the perspective of nitric oxide biology, a favourable antioxidant environment may help to limit the consumption of NO by oxidation and reduce the formation of reactive nitrogen species under conditions of oxidative stress. Therefore, vitamin C may complement rather than directly replicate the effects of dietary nitrate [97].

Other organic acids may also be biologically relevant. For example, fumarate and succinate are intermediates of central metabolic pathways and have important signalling functions in mammalian cells. For example, succinate participates in mitochondrial metabolism and can act as a signalling metabolite through succinate receptor 1 (SUCNR1) [98]. However, the biological effects observed following exposure to purified fumarate or succinate should not be directly extrapolated to beetroot consumption, as the quantities present in food and their bioavailability differ substantially from those in pharmacological interventions [56,92]. Nevertheless, their presence illustrates the metabolic diversity of beetroot and supports the concept that its biological activity results from complex interactions among nutrients and phytochemicals.

In addition to its secondary metabolites, beetroot contains several nutrients that may contribute to cellular and metabolic homeostasis [56,92]. Betaine (trimethylglycine) is of particular interest due to its functions as an osmolyte and a methyl group donor. By participating in homocysteine remethylation, betaine contributes to one-carbon metabolism and may influence methylation-dependent processes [99]. Beetroot also provides minerals such as potassium, magnesium, calcium, phosphorus, iron, copper, zinc and sodium, as well as vitamins including vitamin C and several B vitamins [7,56,68]. Their physiological importance extends beyond basic nutritional value. For example, potassium and magnesium are relevant to vascular and neuromuscular function [100,101], while iron and copper participate in redox and enzymatic processes [102]. It is important to note that the concentrations of these minerals can vary between different beetroot cultivars and preparations, and fermentation can also affect their bioaccessibility by altering the food matrix’s physicochemical properties. Beetroot has also been found to contain both essential and non-essential amino acids, including methionine, cysteine, leucine, isoleucine, histidine and arginine [56,90,92]. Arginine is particularly noteworthy as it is the physiological substrate for NOS-dependent NO production [103]. Beetroot is a complex dietary matrix in which nitrate- and NOS-derived routes of NO production coexist alongside a broader network of nutrients and metabolites.

Triterpenoid saponins are another group of secondary metabolites found in *B. vulgaris*. Several beetroot saponins, including betavulgarosides, predominantly originate from oleanolic acid and related triterpenoid structures. Their composition differs between roots and leaves, and can vary considerably among cultivars [104]. Oleanolic acid and related triterpenoids have demonstrated anti-inflammatory, antioxidant, hepatoprotective and metabolic effects in experimental models [105]. Carotenoids are also present in *B. vulgaris*, particularly in the leaves, where β-carotene and xanthophylls such as lutein have been reported [7,90]. These compounds contribute to antioxidant defence and, in the case of β-carotene, provide provitamin A activity. Lutein and related xanthophylls are also important components of ocular antioxidant protection. However, the carotenoid profile of beetroot roots is less prominent than that of the leaves [2].

Table 1 summarises the main nutritional and bioactive constituents of red beetroot (*Beta vulgaris* L.) before fermentation. It highlights representative compounds and their potential nutritional or biological relevance, as well as the main factors influencing their content. The table provides a concise overview of the basis of the functional properties of beetroot and establishes a reference point for understanding how fermentation may subsequently modify these constituents.

These findings support the view that the biological activity of beetroot stems from the interaction of various compounds. Organic acids, vitamin C, polyphenols, flavonoids, betaine, minerals, amino acids, saponins and other constituents may affect various aspects of cellular homeostasis, such as redox regulation, inflammatory signalling, mitochondrial metabolism, endothelial function and microbial activity. The presence of all these constituents simultaneously may generate additive or, in some cases, synergistic effects that cannot be predicted from studies of isolated compounds.

### 3.4. Factors Affecting Beetroot Composition

The chemical composition of beetroot is not fixed and can vary considerably before fermentation begins. This variability is determined by a combination of genetic, agronomic, environmental, developmental, post-harvest and processing factors. Genotype and cultivar are among the most important factors influencing the concentration and profile of beetroot phytochemicals, including betacyanins and betaine. Consequently, different *B. vulgaris* cultivars can differ considerably in the relative abundance of betacyanins, betaxanthins, and other bioactive constituents [106,107]. In particular, Skalicky et al. (2020) [107] demonstrated substantial differences in betacyanin and betaxanthin profiles among cultivated *B. vulgaris* varieties, confirming that the pigment composition of beetroot is strongly genotype-dependent. Such cultivar-dependent variation is particularly relevant when beetroot is used as a fermentation substrate because differences in the initial concentrations of nitrates, betalains, sugars, and other phytochemicals can affect both microbial growth and the extent of subsequent biochemical transformations [107].

Agricultural and environmental conditions are another major source of compositional variability. Soil characteristics, nitrogen availability, fertilisation practices, water supply, temperature, light exposure, and other environmental factors can influence primary metabolism and the accumulation of secondary metabolites. Nitrogen availability is particularly important for beetroot because it is capable of accumulating substantial amounts of nitrate, the concentration of which may vary according to agricultural practices and growing conditions [54,108]. Environmental conditions affect more than just nitrate accumulation: abiotic stress, temperature and light can also influence pigment synthesis and the accumulation of other phytochemicals in beetroot [1,106]. Therefore, even beetroot belonging to the same cultivar may differ in its biochemical composition if produced under different environmental or agronomic conditions.

The maturity of the plant and the specific tissue analysed may also contribute to compositional differences. The phytochemical and nutritional profiles of the root, peel, leaves, stems and other fractions of *B. vulgaris* are not necessarily identical. This distinction is important because the composition of a fermented product depends on both the cultivar and which plant fraction is used as the fermentation substrate. Comparative analyses of beetroot fractions have demonstrated variations in minerals, fibre, proteins, sugars and bioactive compounds between juice, peel, leaves, stems and pomace [109]. Using whole roots, peeled roots, juices, extracts or preparations incorporating by-products may therefore result in substantially different initial matrices.

Post-harvest handling and storage are additional sources of variability. For example, temperature, oxygen exposure and light can affect the stability of betalains and phenolic compounds, while cutting and mechanical disruption can modify cellular integrity, enzyme activity, and the release or accumulation of phytochemicals [1,75]. It is important to note that post-harvest responses are not necessarily limited to compound degradation. For example, mechanical injury can activate defence-related metabolic pathways, thereby modifying the concentrations of selected phytochemicals. For instance, Preczenhak et al. (2019) [110] reported that treating minimally processed, fresh-cut red beetroot with cysteine increased or maintained the concentrations of several phenolic compounds and betalains during storage. This suggests that post-harvest treatment can actively influence the phytochemical profile of beetroot. This finding shows that storage should not simply be regarded as a passive period during which bioactive compounds decline progressively [110]. Depending on the processing conditions, the biochemical responses of plant tissues may also contribute to changes in their composition.

Applying processing before fermentation can further modify the initial chemical and physical characteristics of beetroot. Methods such as juicing, drying, freezing, boiling, blanching and mechanical disruption can alter cellular structure, water distribution, and the stability or extractability of bioactive compounds [1,25]. The effect of thermal treatment on betalains depends particularly on processing intensity: elevated temperature and prolonged exposure may promote pigment degradation, whereas milder treatments may preserve a substantial proportion of the original pigments [75,111]. Importantly, processing can decrease the concentration of some compounds while increasing their accessibility by disrupting plant cell structures. Therefore, a reduction in the measured concentration of a compound does not necessarily indicate a proportional reduction in its potential biological availability. Studies using different processing technologies have shown that the relationship between processing, compound retention and subsequent bioaccessibility can be complex [112,113].

When considering the biological effects of fermented beetroot, the importance of standardising the starting matrix becomes even greater. A change in the concentration of a particular compound does not necessarily correspond to a proportional change in its bioaccessibility or biological activity. Conversely, a compound that decreases during fermentation may give rise to metabolites with different physicochemical properties or biological effects. Therefore, fermentation should be evaluated not only as a process that changes the concentration of pre-existing compounds, but also as a process that can modify their chemical form, matrix association, stability, and accessibility during digestion. This distinction is particularly relevant for compounds such as phenolic compounds, nitrate/nitrite and betalains, whose biological fate depends on their chemical characteristics as well as their interactions with the food matrix and microorganisms [8,68]. Accordingly, fresh beetroot should be considered a complex biochemical matrix rather than a single-compound delivery system. Its nitrate, betalain, phenolic, fibre, vitamin, mineral, carbohydrate and nitrogen-containing fractions provide distinct substrates and targets for subsequent technological and microbial transformation. The composition of this initial matrix determines the biochemical context in which fermentation occurs and consequently influences the composition of the final product.

## 4. Fermentation of Beetroot

Fermentation is a microbial process whereby microorganisms transform the components of a food matrix into organic acids, gases, alcohols and other metabolites. In plant-based foods, lactic acid fermentation is particularly important because lactic acid bacteria (LAB) can modify the metabolism of carbohydrates, the acidity and texture of foods, and the chemical accessibility of plant-derived phytochemicals [114]. Therefore, fermentation may serve as both a preservation method and a means of modifying the physicochemical and biochemical properties of the original plant matrix. This distinction is particularly relevant in beetroot because the raw material contains several groups of compounds that may respond differently to microbial metabolism, including nitrates, betalains, phenolic compounds, carbohydrates, organic acids and nitrogen-containing metabolites. It is important to note that the term “fermented beetroot” does not describe a single standardised product. Beetroot can undergo either spontaneous fermentation, driven by its indigenous microbiota, or controlled fermentation using selected starter cultures. These approaches can result in substantially different microbial communities and chemical profiles [114,115]. The final composition also depends on whether whole beetroot, slices, pulp or juice is fermented, as well as the salt concentration, temperature, oxygen availability, fermentation time and subsequent storage conditions. Therefore, fermentation should be considered a process-dependent biochemical transformation rather than an inherently beneficial modification of the raw vegetable.

Studies indicate that fermentation can modify the concentrations and profiles of beetroot metabolites, including betalains, phenolic compounds, amino acids, organic acids, volatile compounds and other low-molecular-weight metabolites [26,27,30,116]. For instance, the metabolomic profiles of naturally fermented beetroot juices differ from those of the original raw materials. Furthermore, fermentation with selected LAB can produce strain-dependent changes in betalains, polyphenols, and antioxidant capacity [27,116]. However, fermentation can also result in the degradation, redistribution or transformation of native compounds. This means that an increase in one functional parameter may occur alongside a decrease in another. Therefore, the biological consequences of fermentation cannot be inferred simply from an increase or decrease in the concentration of an individual compound.

### 4.1. Spontaneous and Controlled Fermentation

Beetroot can undergo spontaneous fermentation, whereby microorganisms that are naturally present in the raw material and are introduced from the processing environment initiate the process. Alternatively, controlled fermentation relies on defined starter cultures selected for specific technological or functional characteristics. The two approaches differ in terms of microbial succession, reproducibility, fermentation kinetics and the resulting chemical composition [114,115]. Spontaneous fermentation is traditionally used for vegetable products and can result in complex microbial communities adapted to the plant matrix. The fermentation process is influenced by the initial microbial population, the availability of fermentable carbohydrates, salt concentration, temperature, oxygen exposure, particle size and fermentation duration [114,117]. Specifically with regard to beetroot, Miszczak et al. (2024) [118] demonstrated that fermentation time, temperature and slice thickness significantly impacted the physicochemical properties of the resulting leaven. Fermentation time and temperature were the most influential parameters, whereas slice thickness had a more limited effect, particularly during longer fermentation. A pH below 4.1 was achieved between the fourth and sixth days, depending on the fermentation temperature [118].

The microbial succession during spontaneous vegetable fermentation is also relevant to the safety and biochemical trajectory of the process. In the early stages, facultative anaerobic microorganisms consume residual oxygen. This is followed by the increasing dominance of acid-tolerant LAB, such as *Leuconostoc*, *Weissella* and *Lactobacilli*, as the pH decreases [114,117]. However, this succession is not universal because it depends on factors such as the raw material used, environmental conditions, salt concentration, and the initial microbiota present. Studies of traditional beetroot fermentation have confirmed that temperature and salinity can substantially influence the structure of the microbial community [117].

Naturally fermented beetroot juices have also been shown to differ from fresh beetroot in terms of their metabolomic profiles and antioxidant properties. Kazimierczak et al. (2014) [116] demonstrated that fermented beetroot juices obtained from organic and conventional production systems could be distinguished by metabolomic analysis, exhibiting differences in biological activity. These observations support the view that fermentation can substantially modify the biochemical characteristics of beetroot. However, they do not demonstrate that every fermented beetroot product is physiologically superior to its non-fermented counterpart. Controlled fermentation provides greater control over the microbial population and, consequently, the biochemical trajectory of the process. Choińska et al. (2022) [26] demonstrated that using different starter cultures affects the free amino acid profile, biogenic amine concentrations, and antioxidant properties of fermented red beetroot. These findings are particularly relevant to product development, as they show that the choice of microorganism can affect both desirable and undesirable fermentation products.

Controlled LAB fermentation has also been used to investigate changes in beetroot betalains and phenolic compounds. Sawicki et al. (2024) [27] compared the effects of different species of LAB, including *Lactobacillus acidophilus*, *Levilactobacillus brevis* and *Lactiplantibacillus plantarum*, on the profiles of betalains and polyphenols, as well as on antioxidant and other in vitro biological activities. Among other changes, the authors reported the formation of isobetanin and neobetanin, as well as increases in selected flavonoids, including rutin, kaempferol, and (+)-catechin [27]. These results highlight the importance of considering fermentation as a strain-dependent biotransformation rather than a uniform technological treatment. This point is reinforced by studies using different microbial consortia. Casciano et al. (2022) [115] demonstrated that different fermentation approaches and microbial consortia affect the microbiological, physicochemical, structural, and volatile profiles of red beetroot. Specific starter combinations are associated with distinct volatile organic compounds [115].

An important consequence of these observations is that fermentation-induced changes should not automatically be interpreted as improvements. A reduction in the concentration of a native compound may coexist with the formation, release or redistribution of other compounds that have different chemical or biological properties. For instance, changes in betalain composition may occur even when total betalain concentration decreases [27]. Therefore, the effects of fermentation should be evaluated using a combination of quantitative compositional measurements, metabolite profiling, bioaccessibility studies and appropriate biological assays.

### 4.2. Microorganisms and Fermentation Parameters

LAB are the main microbial group studied in the context of controlled vegetable fermentation. They convert fermentable carbohydrates primarily into lactic acid through metabolism, although heterofermentative species can also produce carbon dioxide, ethanol and acetic acid [114]. The progressive acidification that occurs changes the physicochemical environment, contributing to preservation by restricting the growth of many spoilage and potentially pathogenic microorganisms. Studies focusing on beetroot have identified several LAB taxa associated with fermentation. For example, Kumari et al. (2022) [119] isolated *Lactobacillus* strains from fermented beetroot and characterised their probiotic and metabolic properties. Similarly, Maślak et al. (2022) [120] identified *Lactococcus lactis* and *Weissella cibaria* in fermented beetroot, evaluating their technological and probiotic potential. These findings demonstrate that beetroot can support the growth of microorganisms with potentially useful technological properties [119,120]. However, the isolation of a potentially probiotic strain from fermented beetroot does not constitute evidence that the fermented product itself is a probiotic food or produces probiotic effects in humans. A probiotic claim requires evidence that the microorganism is present in an adequate viable quantity, survives relevant gastrointestinal conditions and provides a demonstrated health benefit to the intended host.

The outcome of the fermentation of beetroot depends on several interacting parameters, including the microbial strain or consortium used, the temperature, the duration of the process, the salt concentration, the pH level, the concentration of the substrate, the availability of oxygen and the particle size [114,117,118]. Miszczak et al. (2024) [118] demonstrated the importance of fermentation time and temperature in particular, while Janiszewska–Turak et al. (2024) [121] showed that salt concentration can influence the content of betalains and other physicochemical characteristics of fermented red beetroot [118,121].

Particle size is important because mechanical disruption increases the surface area available to microorganisms, which can affect mass transfer, substrate accessibility, and the release of intracellular compounds. This is particularly important when comparing the fermentation of whole or sliced beetroot with the fermentation of beetroot juice because the physical structure of the matrix can substantially alter microbial access to nutrients and phytochemicals [121,122].

Temperature influences microbial growth and enzyme activity, thereby affecting the rate and direction of metabolite formation. The duration of fermentation determines the extent of carbohydrate utilisation, acidification and microbial transformation. If fermentation is too short, acidification may be incomplete, whereas if it is prolonged, some sensitive phytochemicals may degrade or undergo further transformation. Consequently, an optimal fermentation time for microbiological stabilisation may not necessarily preserve every bioactive constituent [123,124].

The pH trajectory is another critical factor. The production of organic acids progressively lowers the pH, creating a more selective environment that favours acid-tolerant microorganisms. Changes in pH can affect pigment stability, phenolic chemistry, enzyme activity and microbial safety simultaneously. This is particularly relevant to betalains, whose stability is strongly influenced by environmental conditions [125]. Therefore, acidification can have different effects on the various components of the beetroot matrix; it can contribute to the preservation of certain compounds under specific conditions, while promoting the transformation or degradation of others [115].

Evidence from the fermentation of vegetables suggests that the concentrations of nitrates and nitrites can change during the fermentation process. However, the direction and magnitude of these changes depend strongly on the microbial community and the conditions of the process. In fermented vegetables, for example, nitrate levels may decrease during fermentation, whereas nitrite levels can increase temporarily in the early stages before declining as acidification progresses [126,127]. Notably, nitrate reduction is not necessarily driven by LAB themselves. Previous research involving fermented carrots revealed that the initial nitrate reduction was linked to Gram-negative bacteria present in the initial fermentation stage, whereas the LAB used as starter cultures had little impact on nitrate concentration [128]. At the same time, nitrate-reducing LAB have been identified in fermented vegetable systems, and strain-specific nitrate and nitrite metabolism has been documented [129]. Therefore, nitrate metabolism during fermentation cannot be predicted solely from the designation “LAB”; it must be determined experimentally for each particular strain or microbial consortium.

This distinction is particularly important for beetroot. Although beetroot is naturally nitrate-rich, a reduction in nitrate concentration during fermentation does not necessarily represent a loss of physiological value, nor does an increase in nitrite necessarily imply increased NO production in the consumer. The oral microbiome plays a significant role in converting dietary nitrate to nitrite, which can then participate in NO formation under suitable physiological conditions [130,131]. Therefore, where possible, measurements of nitrate and nitrite in the fermented product should be complemented by studies of nitrate absorption, plasma nitrate/nitrite concentrations, urinary nitrate excretion or other relevant pharmacokinetic endpoints. For this reason, future studies of fermented beetroot intended to support claims concerning cardiovascular or NO-related effects should report nitrate and nitrite concentrations before and after fermentation, rather than assuming that fermentation increases nitrate bioavailability. This is particularly important given that recent research into fermented vegetables has demonstrated that uncontrolled nitrate reduction and nitrite accumulation may also pose a food safety concern in certain circumstances [132].

Fermentation can generate metabolites that are either absent or present at low concentrations in the original vegetable. Depending on the substrate and microbial community involved, these may include lactate, acetate, other organic acids, microbial peptides, modified phenolic compounds, volatile metabolites, and other products resulting from microbial transformation [114,115]. Recent studies focusing on beetroot support this interpretation. For example, Jakubczyk et al. (2024) [30] reported changes in the phenolic composition and antioxidant properties of fermented beetroot juices, and Sawicki et al. (2024) [27] demonstrated that different LAB species produce distinct changes in betalains and polyphenols. These findings suggest that microbial identity plays a significant role in determining the final product, rather than being merely a technical parameter.

### 4.3. Product Heterogeneity, Safety and Standardisation

A major challenge when evaluating fermented beetroot is the significant variation in the products described in the literature. Preparations may differ in terms of cultivar, plant maturity, cultivation system, composition of raw materials, particle size, water-to-solid ratio, salt concentration, fermentation substrate, microbial inoculum, fermentation temperature and duration, exposure to oxygen, pH endpoint, storage conditions, and post-fermentation treatment. Consequently, results obtained with one preparation cannot automatically be extrapolated to another. This is particularly important when comparing fermented and fresh beetroot. Differences in biological activity may result from variations in nitrate dosage, concentration of betalain pigments, phenolic profile, sugar content, acidity, microbial metabolites or other components, rather than from fermentation itself. Ideally, therefore, comparative studies should use the same cultivar and starting material, reporting the complete formulation and fermentation conditions. Where possible, fermented and non-fermented products should also be compared based on the concentrations of relevant constituents rather than simply on product mass or volume.

The choice of microorganism is another major source of variation. Controlled fermentation can improve reproducibility by introducing defined starter cultures, whereas spontaneous fermentation involves variable microbial communities and microbial succession. For example, Choińska et al. (2022) [26] demonstrated that the selection of a starter culture affected the levels of free amino acids, biogenic amines, and antioxidant properties in fermented red beetroot. Similarly, using different microbial consortia can generate distinct volatile and sensory profiles, showing that microbial composition affects the technological characteristics and biochemical identity of the final product [115].

Beetroot juices and other minimally processed products present particular concerns in terms of food safety. Fresh, unpasteurised vegetable juices may contain spoilage microorganisms and foodborne pathogens, and inadequate processing or storage can increase microbiological risks. Although fermentation can contribute to microbial control through acidification, competition and the production of antimicrobial metabolites, it should not be considered a substitute for hygienic processing and validated safety controls. The safety of the final product depends on the initial microbiological quality of the raw material, the kinetics of fermentation, the achieved pH level and salt concentration, the microbial community, processing hygiene, and storage conditions [114].

Biogenic amines are another parameter that requires attention. These compounds can be produced by the decarboxylation of amino acids by microbes and can accumulate during fermentation depending on the microorganism and environmental conditions. Choińska et al. (2022) [26] specifically demonstrated that the selection of the starter culture influenced the concentration of biogenic amines in fermented red beetroot. This further illustrates why it should not be assumed that fermentation reduces all undesirable compounds. Instead, selecting the right starter and controlling the process are essential for achieving a favourable balance between the desired microbial metabolites and the potentially undesirable products. Therefore, standardisation should encompass both chemical and microbiological parameters. At a minimum, studies should report the initial and final concentrations of nitrate and nitrite, major betalains, relevant phenolic compounds, pH, titratable acidity, fermentation temperature and duration, salt concentration, microbial composition or starter strain and storage conditions. For studies addressing the proposed nitrate–nitrite–NO mechanism, the measurement of nitrate and nitrite should be considered essential rather than optional. Where relevant, biogenic amines, microbial counts, and microbiological safety indicators should also be reported.

Another issue concerns the interpretation of broad antioxidant assays as indicators of functional improvement. Studies of fermented beetroot often report FRAP, ABTS, DPPH, total phenolic content and total flavonoid content. While these parameters are useful for characterising chemical antioxidant capacity and phytochemical composition, they do not directly demonstrate anti-inflammatory activity, improved vascular function, modulation of cellular redox signalling or clinical benefit. Indeed, Sawicki et al. (2024) [27] demonstrated increased antioxidant activity in selected fermented beetroot juices alongside changes in the composition of betalains and polyphenols, but these observations were primarily obtained in chemical and in vitro models. While such findings provide valuable evidence, they should be clearly distinguished from evidence obtained in animal models or human intervention studies.

Thus, current evidence suggests that beetroot fermentation is a highly context-dependent process. Spontaneous and controlled fermentation can produce different microbial and chemical profiles, and the effects of factors such as starter culture, temperature, duration, salt concentration, particle size, and other processing parameters may be substantial [117,118,121]. Therefore, the term “fermented beetroot” should not be interpreted as referring to a single, reproducible functional food intervention. Instead, it describes a diverse group of products whose composition and biological properties depend on the characteristics of the initial beetroot and the fermentation process. This process-dependent heterogeneity is particularly relevant to the interpretation of nitrate–nitrite–NO signalling. Fermentation can alter the chemical form and concentration of nitrates and nitrites while also changing the levels of betalains, phenolic compounds, organic acids, and microbial metabolites. The resulting biological response is therefore likely to reflect the combined properties of the fermented matrix rather than the action of a single compound. The next section consequently examines how fermentation affects specific components of beetroot, including nitrates, nitrites, betalains, phenolic compounds, organic acids and other metabolites, and whether these compositional changes are accompanied by altered bioaccessibility and biological activity.

## 5. Effects of Fermentation on Beetroot Composition and Bioaccessibility

Fermentation is not merely a preservation step; it is a process that can alter the chemical composition and physical organisation of the beetroot matrix. Microbial growth, enzymatic activity, acidification and changes in plant cell structure can release compounds that were previously bound to cell walls or other matrix components while simultaneously transforming or degrading some native constituents. Consequently, the effects of fermentation depend heavily on the microorganism used, the fermentation conditions, the characteristics of the raw material and the composition of the final food matrix. Studies specifically focusing on beetroot have reported changes in profiles of betalain, phenolic composition, antioxidant capacity and metabolite patterns following fermentation. However, the direction and magnitude of these changes are not consistent [25,30,46,133].

It is therefore important to distinguish between chemical composition, bioaccessibility, bioavailability and biological activity. An increase in the concentration of a compound in a fermented product does not necessarily mean that more of it will be released during gastrointestinal digestion or absorbed into the bloodstream. Similarly, an increase in an in vitro antioxidant assay cannot be directly interpreted as evidence of enhanced antioxidant protection in humans. Fermentation may also generate metabolites with different biological properties to those of the original beetroot constituents. Therefore, the nutritional significance of fermentation must be evaluated at multiple levels: first, changes in the food matrix and chemical profile; then, gastrointestinal release and bioaccessibility; then, absorption and systemic exposure; and finally, biological effects in appropriate experimental or clinical models [46,133].

### 5.1. Nitrate and Nitrite

Although nitrate is one of the principal bioactive constituents of beetroot, its concentration can vary substantially depending on the cultivar, growing conditions, agricultural practices and processing methods used. Fermentation introduces an additional metabolic variable because the microorganisms involved may modify the food’s chemical environment and participate in nitrate reduction, depending on the microorganisms and fermentation conditions. Therefore, fermentation should not simply be regarded as a process that increases the bioavailability of nitrates and nitrites; it may also alter the chemical form, distribution and subsequent metabolic fate of nitrogen-containing compounds in the beetroot matrix. Currently, available evidence does not support the conclusion that fermentation consistently increases the bioavailability of nitrates or nitrites. This distinction is important because the nitrate present in fermented foods, the nitrite generated during fermentation, the microbial reduction in nitrate in the oral cavity, the circulating nitrate and nitrite, and the subsequent formation of nitric oxide represent interconnected but distinct stages of nitrate metabolism [130,134].

Particular caution is warranted when interpreting changes in nitrite levels. According to experimental evidence from fermented food systems, nitrate originating from beetroot can be converted to nitrite during fermentation. However, the extent of this conversion depends on the food matrix and processing conditions [135]. At the same time, however, fermentation may also promote microbial processes that limit nitrite accumulation. For instance, changes in the bacterial community during sauerkraut fermentation have been linked to variations in nitrite concentration. Lactic acid bacteria, including species such as *Lactobacillus* and *Leuconostoc*, have been found to inhibit nitrate-reducing bacteria, thereby reducing nitrite accumulation [136]. These observations demonstrate that the net nitrate–nitrite balance during fermentation is the result of competing microbial and chemical processes rather than a predictable, one-directional conversion. Results obtained with vegetable powders in fermented meat products further demonstrate that the final nitrite concentration depends on the food matrix’s composition and the processing conditions used [137].

Importantly, the physiological significance of dietary nitrate goes beyond its concentration in fermented foods. After ingestion, a significant amount of the nitrate in the bloodstream is actively absorbed by the salivary glands and released into the mouth, where nitrate-reducing bacteria convert some of it into nitrite. This nitrite can then contribute to the formation of nitric oxide in the body, particularly in situations of low oxygen availability and low pH [12,134]. Therefore, the oral microbiome is an essential component of the dietary nitrate–nitrite–NO pathway. Disruption to oral bacterial nitrate reduction can markedly reduce the increase in circulating nitrite and some of the vascular effects observed after consuming beetroot rich in nitrates [41,131].

Consistent with this mechanism, numerous clinical studies have demonstrated that nitrate-rich beetroot preparations can increase circulating nitrate and nitrite levels, influencing vascular function and blood pressure [35,138,139,140]. However, these findings demonstrate the physiological potential of dietary nitrate and should not be interpreted as evidence of a specific benefit attributable to fermentation. Systematic reviews and meta-analyses suggest that beetroot-derived dietary nitrate can increase circulating nitrate and nitrite levels, potentially improving certain vascular outcomes. However, the extent of the response depends on factors such as dosage, duration, population characteristics, and the formulation of the nitrate source [14,36,141]. Therefore, for fermented beetroot, the critical question is not simply whether fermentation increases the nitrate content, but whether it modifies the availability and metabolic conversion of nitrate in a way that remains after digestion and is integrated into the oral nitrate–nitrite–NO pathway. This question remains insufficiently investigated and represents an important area for future experimental and clinical research.

### 5.2. Betalains

Betalains are the characteristic bioactive pigments found in red beetroot. They are divided into two main groups: red-violet betacyanins and yellow-orange betaxanthins. Their chemical stability is strongly influenced by processing conditions, particularly temperature, pH, oxygen, light, water activity and the composition of the surrounding matrix [4,111,142]. Because fermentation simultaneously alters the acidity, microbial activity, matrix structure and chemical environment of the product, the effects on betalains are unlikely to be explained by a single degradation mechanism; fermentation should therefore be considered a dynamic process capable of modifying the quantity and distribution of individual betalain compounds.

Evidence from the red beetroot itself supports this interpretation. Sawicki and Wiczkowski (2018) [25] demonstrated that boiling and spontaneous fermentation both substantially altered the profile of the beetroot’s betalains. The total content of betalains decreased during fermentation, with losses reported in the range of 61–88% depending on the product and processing conditions. Betanin, isobetanin, betanidin and vulgaxanthin I were among the predominant pigments identified. Importantly, the reduction in total betalain content was accompanied by changes in the relative contribution of individual pigments. This indicates that fermentation affects the betalain profile rather than simply causing uniform pigment loss. The authors suggested that acidification, matrix softening, leaching and microbial activity may all contribute to these changes [25]. Therefore, fermentation cannot be considered a process that invariably preserves or increases the concentration of betalains.

At the same time, a decrease in total betalain concentration does not necessarily result in a proportional reduction in functional activity. Fermentation can disrupt plant tissue and alter the distribution of pigments between the soluble and matrix-associated fractions. This can potentially increase the extractability of certain compounds, even when the total pigment content decreases. This distinction is particularly relevant when antioxidant activity is used as an endpoint [26,30,116]. A change in measured antioxidant capacity may reflect changes in the absolute amount of betalains, pigment composition, extractability, degradation products and interactions with other fermentation-derived compounds. Consequently, measurements of total betalains should ideally be complemented by compound-specific profiling and independent assessments of biological activity.

More recent evidence obtained using fermented red beetroot juice further demonstrates that the outcome depends on the microorganism used for fermentation. Sawicki et al. (2024) [27] compared fermentation by different lactic acid bacteria and found that the bacterial species affected the profiles of both betalains and polyphenols, as well as the resulting in vitro bioactivity of the fermented juice. These findings suggest that “fermented beetroot” is not a chemically uniform product and that the final betalain profile is influenced by the identity of the fermenting microorganism and the fermentation conditions. This strain- or species-dependent effect is particularly important when comparing spontaneous fermentation with controlled fermentation using selected lactic acid bacteria.

The effect of fermentation on betalains should also be considered in the context of changes to the physicochemical environment. Fermentation-associated acidification may affect pigment stability, while microbial growth, oxygen exposure and changes in the plant matrix may influence the degradation, release or transformation of individual compounds. However, the direction of these effects cannot be predicted solely from the final pH or total betalain concentration because the stability of individual betalains differs, and multiple factors may act simultaneously. Betacyanins and betaxanthins do not necessarily exhibit identical stability under processing conditions, and individual compounds such as betanin, isobetanin, betanidin derivatives and vulgaxanthin I may follow different degradation or transformation pathways [111,142].

Thus, the available evidence suggests that, while fermentation can substantially alter the composition of red beetroot’s betalain pigments, it does not consistently improve their stability or bioavailability. Rather, it seems that the outcome depends on factors such as the microbial species or strain, fermentation duration, acidification, oxygen exposure, temperature, and the characteristics of the original beetroot matrix. For this reason, future studies should not only measure total betalain content, but also report on individual pigments whenever analytical methods permit.

### 5.3. Polyphenols and Antioxidant Capacity

Fermentation can substantially modify the phenolic composition of beetroot by altering the chemical environment and accessibility of compounds within the plant matrix. Microbial enzymes can break down the bonds that link phenolic compounds to sugars, cell wall components or other matrix constituents, thereby releasing them into the soluble fraction. At the same time, microbial metabolism may transform or degrade individual phenolic compounds. The resulting changes should therefore be understood as a redistribution and biotransformation of phenolic compounds rather than an overall increase in their content [143,144]. This distinction is important because changes in the concentration, chemical form and accessibility of individual compounds may have different nutritional and biological consequences. Evidence specific to beetroot supports this interpretation. Płatosz et al. (2020) [133] identified phenolic acids and flavonoids in predominantly bound or conjugated forms in fresh beetroot, and demonstrated that fermentation increased the proportion of free phenolic acids while reducing the amount of conjugated forms present. Consequently, the phenolic profile was altered at the level of individual compounds, with isoferulic acid, protocatechuic acid, epicatechin and apigenin being the predominant compounds detected. Notably, these findings suggest that an apparent increase in the free fraction does not necessarily signify the synthesis of new phenolic compounds. Rather, it may reflect the release or conversion of compounds that were previously bound to the plant matrix. Such changes may affect the compounds’ solubility and accessibility to digestive processes. However, their ultimate biological significance depends on what happens to them after ingestion [133]. Thus, an increase in a particular phenolic fraction should not automatically be interpreted as an increase in the total amount of biologically available phenolic compounds (Figure 2).

The extent and direction of these changes may depend on the formulation of the fermented product. Jakubczyk et al. (2024) [30] reported differences in the phenolic composition and antioxidant properties of fermented beetroot juices prepared using various additives. The tested formulations differed in terms of their total phenolic and flavonoid content, as well as their antioxidant capacity, which was measured using several in vitro assays. These findings suggest that fermentation and substrate composition can both influence the chemical antioxidant properties of beetroot beverages. They also reinforce the importance of considering fermentation as a matrix-dependent process in which the final composition reflects the interactions between the original beetroot constituents, the added ingredients and the microbial metabolism. However, it should be noted that antioxidant capacity measured in vitro does not equate to antioxidant protection in humans.

Recent evidence from fermented beetroot products demonstrates that changes in phenolic content do not necessarily predict their subsequent bioaccessibility. Mana de Wolf et al. (2025) [46] showed that lactic fermentation increased the concentration of free phenolics in both a low-fibre beetroot juice and a high-fibre puree-based beverage. Despite this similar compositional response, the two matrices behaved differently during simulated gastrointestinal digestion: fermentation decreased the in vitro bioaccessibility of phenolics in the juice but increased their bioaccessibility in the puree-based beverage. These findings provide direct evidence that the same fermentation process can have opposite consequences for phenolic bioaccessibility depending on the structure and composition of the food matrix [46].

Therefore, the food matrix should be regarded as an active determinant of the fate of phenolic compounds rather than a passive carrier. Phenolic compounds may interact with dietary fibre, proteins, polysaccharides, minerals and other food constituents [145]. Fermentation can alter these interactions through enzymatic hydrolysis, acidification and structural changes to the matrix. In this context, the findings of Mana de Wolf et al. (2025) [46] are particularly relevant because they demonstrate that a higher proportion of free phenolic compounds does not necessarily result in greater gastrointestinal accessibility. In fermented beetroot juice, for example, an increase in free phenolic compounds was accompanied by lower bioaccessibility, showing that release from the original matrix and accessibility during gastrointestinal digestion are distinct processes. Consequently, measurements performed before digestion cannot reliably predict the fraction that will become available for intestinal uptake.

It is also essential to understand the distinction between bioaccessibility and bioavailability when considering the potential biological effects of fermented beetroot. Bioaccessibility describes the fraction of a compound released from the food matrix during digestion that is potentially available for absorption. Bioavailability, however, also depends on intestinal uptake, metabolism, transport, systemic exposure and tissue distribution [146,147,148]. Phenolic compounds undergo extensive transformation in the gastrointestinal tract and liver, and their circulating metabolites can differ substantially from the parent compounds found in food [149,150,151]. Therefore, an increase in free phenolic compounds in fermented beetroot should not be interpreted as evidence of increased systemic exposure in the absence of supporting pharmacokinetic or human intervention data [30,133].

Thus, the available evidence suggests that fermentation can significantly alter the phenolic profile and in vitro antioxidant properties of beetroot. However, these effects are inconsistent and do not necessarily reflect biological activity in humans. The outcome depends on microbial species or strains, fermentation conditions, substrate composition and the food matrix, as well as subsequent processes occurring during digestion and absorption. For this reason, future studies should combine compound-specific phenolic profiling with simulated gastrointestinal digestion and assessment of bioaccessibility.

### 5.4. Fermentation-Derived Metabolites and Bioaccessibility

An important consequence of beetroot fermentation is the remodelling of its chemical environment and the formation or enrichment of compounds that were absent from, or present only in low concentrations in, the original plant matrix. During lactic acid fermentation, microorganisms utilise available carbohydrates and other substrates to produce lactic acid, as well as other organic acids and microbial metabolites. At the same time, the accompanying changes in pH and matrix structure, as well as microbial enzymes, can modify phenolic compounds, betalains, and other plant constituents [27,133]. Thus, fermentation not only preserves the original phytochemical composition of beetroot, but also creates a matrix that is constantly changing, in which plant-derived compounds and microbial metabolites coexist and influence one another.

These changes can be particularly significant during gastrointestinal digestion. Microbial enzymatic activity can alter the molecular structure of phenolic compounds by breaking down glycosidic, ester, and other chemical bonds. This can potentially increase the proportion of compounds that are more easily released from the food matrix [152]. However, this should not be equated with improved absorption. Fermentation can also produce metabolites with different physicochemical properties and biological activities to the parent compounds. Evidence from fermented beetroot illustrates this concept: Płatosz et al. (2020) [133] observed changes in the balance between free and conjugated phenolic acids, as well as alterations in the flavonoid profile, following fermentation. These findings suggest that fermentation may influence both the quantity of phenolic compounds measured in food and the forms in which they subsequently enter the gastrointestinal environment.

The same principle applies to betalains, although their response to fermentation is more complex as release and degradation can happen at the same time. Disruption to the plant matrix during fermentation can facilitate the transfer of pigments into the liquid phase, while acidification, microbial activity, oxygen exposure and other physicochemical factors can promote the transformation or loss of individual betalains [3,121]. Sawicki and Wiczkowski (2018) [25] demonstrated that fermentation altered the profile of the red beetroot’s betalains and was accompanied by a substantial reduction in total content. Consequently, increased extractability of a pigment from fermented beetroot should not be interpreted as evidence of improved retention or bioavailability; rather, extractability, chemical stability, bioaccessibility and bioavailability represent distinct stages in the fate of beetroot phytochemicals.

The importance of this distinction is further illustrated by studies of the bioaccessibility of phytochemicals in beetroot. Deseva et al. (2020) [146] demonstrated that the bioaccessibility of phytochemicals in red beetroot juice changed significantly during simulated gastrointestinal digestion. This shows that the compounds present in the original beverage are not necessarily the same as those that remain available after digestion. For fermented beetroot, this issue is even more pertinent as fermentation alters the phytochemical composition and physical structure of the food matrix prior to digestion. The quantity of a compound available for absorption is therefore contingent upon the cumulative impact of fermentation, matrix interactions, digestive conditions, microbial metabolism and intestinal uptake.

The food matrix plays a key role in determining these outcomes. Phenolic compounds can interact with dietary fibre, proteins, polysaccharides and other matrix constituents, and fermentation can modify these interactions via acidification, enzymatic hydrolysis and structural changes. Recent evidence from fermented beetroot products suggests that an increase in free phenolic compounds may be accompanied by different changes in bioaccessibility, depending on the matrix used [46]. This observation highlights the importance of considering fermentation-derived changes in chemical composition alongside the characteristics of the fermented substrate and gastrointestinal digestion conditions. Accordingly, the biological significance of fermentation-derived metabolites should be evaluated across several levels, from chemical characterisation to gastrointestinal fate and ultimately biological response. Future studies should identify and quantify individual fermentation-derived metabolites, determine their chemical stability, assess their release and transformation during simulated gastrointestinal digestion and distinguish bioaccessibility from actual absorption and systemic bioavailability. Where appropriate, these approaches should be complemented by cellular, animal and human studies.

Table 2 summarises the currently available experimental evidence on how fermentation modifies the composition, stability, bioaccessibility and functional properties of beetroot products. Particular attention is given to changes in betalains, phenolic compounds and fermentation-derived metabolites, as well as to the effects on gut microbiota and in vitro or in vivo functional responses. The distinction is made between food-model, gastrointestinal digestion, microbiological and animal studies.

Thus, the current evidence suggests that fermentation can reconfigure, rather than simply enrich, the beetroot matrix. It can promote the release of certain matrix-associated phytochemicals, transform others and generate organic acids and other microbial metabolites. It can also alter the physicochemical conditions that determine gastrointestinal accessibility. However, the direction and magnitude of these effects vary among fermentation systems. Direct evidence that fermented beetroot consistently provides greater systemic bioavailability than compositionally matched fresh beetroot remains insufficient.

## 6. Potential Biological Mechanisms

The biological effects of beetroot-derived products are unlikely to be attributable to a single constituent. Instead, they are probably the result of the combined activity of several chemically and biologically distinct components, including dietary nitrate, betalains, phenolic compounds, organic acids and fermentation-derived metabolites. Of these, nitrate has the most clearly established link with vascular physiology via the nitrate–nitrite–NO pathway [2,68]. Betalains and phenolic compounds, on the other hand, may contribute to the modulation of redox-sensitive and inflammatory processes [22]. Fermentation adds another level of complexity as it can alter the chemical form of plant constituents, modify the food matrix, alter microbial populations and generate metabolites such as organic acids and other low-molecular-weight compounds [2,115]. Recent work on lactic-fermented beetroot beverages illustrates this complexity: fermentation increased the content of free phenolics and the antioxidant activity of some products, but the effect on phenolic bioaccessibility depended on whether the product was prepared as a juice or a fibre-rich purée [46]. Nevertheless, this framework should be interpreted according to the strength of the available evidence. The nitrate–nitrite–NO pathway has been extensively characterised in humans, with studies examining the effects of nitrate salts, nitrate-rich vegetables and beetroot products. In contrast, evidence directly demonstrating that fermentation-induced changes in beetroot composition result in specific physiological effects in humans is limited.

### 6.1. Nitrate–Nitrite–NO Pathway

The nitrate–nitrite–NO pathway is the best-established mechanism through which nitrate-rich beetroot exerts its effects on the vasculature. Dietary nitrate (NO_3_^−^) is absorbed from the gastrointestinal tract and enters the systemic circulation. It is then concentrated in saliva via the enterosalivary circulation. In the oral cavity, commensal nitrate-reducing bacteria then convert the nitrate to nitrite (NO_2_^−^), which initiates the reductive pathway that contributes to the formation of NO [157,158].

Nitrate, nitrite and NO represent the different stages of this pathway. Nitrite is a more proximal intermediate and circulating reservoir for NO bioactivity. Following swallowing, some of the nitrite undergoes reactions in the acidic stomach, while the rest enters the circulation where it can be reduced to NO through enzymatic and non-enzymatic mechanisms involving deoxyhaemoglobin, myoglobin and xanthine oxidoreductase, among others [158,159,160]. These reactions are particularly important under hypoxic, acidic, or otherwise oxygen-limited conditions, as they complement the canonical oxygen-dependent L-arginine-eNOS pathway. Once formed, NO activates soluble guanylate cyclase in vascular smooth muscle, increasing cGMP signalling and promoting vasodilation [158,159,160]. This mechanism provides a biological basis for the vascular effects observed after consuming nitrate-rich beetroot products.

Human intervention studies and meta-analyses have reported increases in circulating nitrate and nitrite, improvements in endothelial function, and reductions in systolic blood pressure following beetroot or inorganic nitrate supplementation [65,161]. However, responses vary between populations and outcomes. A recent meta-analysis in individuals with hypertension found reduced clinical systolic blood pressure following nitrate-rich beetroot juice consumption, but no significant effect on 24 h blood pressure, with the overall certainty of evidence considered low [36]. Thus, although the nitrate–nitrite–NO pathway provides a coherent mechanistic basis for vascular effects, the magnitude of the response is not uniform.

A major determinant of this variability is the oral microbiome. The efficiency of nitrate-to-nitrite conversion depends not only on nitrate intake but also on oral nitrate-reducing activity, food matrix characteristics, baseline vascular function, and individual factors such as age [157,160]. Antibacterial mouthwash can inhibit oral nitrate reduction and attenuate some vascular responses to dietary nitrate, supporting a causal role for oral bacteria in nitrate bioactivation [157,162]. Dietary nitrate can also modify the abundance of oral taxa involved in nitrate metabolism, including *Rothia* and *Neisseria*, although changes in bacterial abundance do not necessarily translate into enhanced vascular responses. Burleigh et al. (2019) [162], for example, observed alterations in the oral microbiome following nitrate supplementation without an increased vascular response to a subsequent acute nitrate dose. This indicates that microbial composition, nitrate-reducing activity, substrate availability, and host physiology represent related but distinct determinants of NO bioavailability (Figure 3) [163].

Further pharmacokinetic evidence demonstrates why nitrate content alone cannot be equated with NO bioavailability. Consuming beetroot produces dose-dependent increases in circulating nitrate and changes in nitrite concentrations, but these responses may differ between individuals and age groups. In a randomised crossover study, whole beetroot was found to increase plasma, salivary and urinary nitrate levels in both younger and older adults, while plasma nitrite levels were lower in the older participants [164]. Therefore, the final biological response depends on a sequence of processes encompassing absorption, salivary concentration, microbial nitrate reduction, systemic nitrite handling and tissue-level NO formation.

These considerations are particularly important for fermented beetroot. Fermentation can modify the physical and chemical properties of the matrix, potentially influencing the release, stability and gastrointestinal handling of nitrates and other constituents. Nevertheless, current evidence does not demonstrate that fermentation consistently enhances nitrate-to-nitrite conversion or systemic NO availability. Research on fermented beetroot has predominantly focused on phenolic compounds, antioxidant activity, microbial transformation, and physicochemical characteristics, rather than making direct comparisons of nitrate pharmacokinetics and vascular responses between fermented and non-fermented products. For example, Mana de Wolf et al. (2025) [46] demonstrated substantial fermentation- and matrix-dependent changes in phenolic content and bioaccessibility, but did not establish an enhanced nitrate–nitrite–NO response. Therefore, compositional changes induced by fermentation should not be interpreted as evidence of increased NO bioavailability without direct pharmacokinetic or physiological confirmation.

Individual differences in nitrate-reducing capacity are an additional source of variability. Sex-related differences have been reported, with women exhibiting greater oral nitrate-reducing activity and higher plasma nitrite concentrations than men following the supplementation of inorganic nitrates, despite comparable plasma nitrate levels [165]. These functional differences could not be explained by significant variations in the overall composition of the salivary microbiome, which further suggests that microbial activity may be a more reliable indicator than taxonomic abundance alone.

Therefore, the nitrate–nitrite–NO pathway is a well-supported explanation for the vascular effects of nitrate-rich beetroot: dietary nitrate enters the enterosalivary cycle, where it is reduced to nitrite by oral bacteria. This nitrite can then contribute to systemic NO formation and vascular signalling [157,158,159,160]. However, it remains unclear whether fermentation modifies these processes sufficiently to increase NO bioavailability or improve physiological outcomes. The available evidence therefore supports the biological plausibility of such an effect, but not its demonstration. Direct comparative studies of fermented and non-fermented beetroot, including nitrate and nitrite pharmacokinetics, oral nitrate-reducing activity, NO-related biomarkers and vascular outcomes, are needed to determine whether fermentation provides a meaningful functional advantage.

### 6.2. Redox and Inflammatory Regulation

Redox homeostasis is closely linked to nitric oxide (NO) signalling and plays a key role in the vascular, metabolic and inflammatory effects of beetroot. Under physiological conditions, NO promotes vasodilation and endothelial function; however, excessive reactive oxygen species (ROS) can reduce NO bioavailability and impair endothelial signalling. Conversely, the nitrate–nitrite–NO pathway can increase NO availability via an NO synthase-independent mechanism, which is one of the bases for the vascular effects of nitrate-rich beetroot [166,167].

Another component of beetroot’s redox activity is provided by its pigments, the betalains, particularly betanin and related betacyanins. Beyond direct radical scavenging, these compounds may influence endogenous antioxidant defences. Experimental studies have demonstrated that betanin protects against oxidative DNA damage and activates the nuclear factor erythroid 2-related factor 2 (Nrf2) pathway. This is accompanied by increased expression of haem oxygenase-1 (HMOX1), paraoxonase-1 (PON1) and glutathione (GSH) [7,168,169]. The Nrf2/Keap1 system provides a plausible mechanism for this effect: oxidative or electrophilic stimuli can promote Nrf2 accumulation and nuclear translocation, leading to the expression of genes involved in antioxidant defence and cellular protection [169]. However, these findings predominantly derive from experimental models and cannot be directly equated with Nrf2 activation following dietary beetroot consumption in humans.

Fermentation can increase the redox potential by altering native phytochemicals and creating new bioactive metabolites. An example of this is 5-hydroxymaltol, which was identified following the fermentation of beetroot juice with Lactobacillus spp. In experimental models, this fermentation-derived compound reduced oxidative stress and inflammatory responses, as well as modulating Nrf2/HO-1 and NF-κB/MAPK signalling [170]. While this lends support to the idea of fermented beetroot as a biologically active, multi-component matrix, the contribution of 5-hydroxymaltol to the effects of consuming fermented beetroot remains to be established in humans.

Changes in antioxidant capacity observed after fermentation provide additional, albeit indirect, evidence of altered redox potential. Fermented beetroot juices have demonstrated substantial radical-scavenging and reducing activity, with reported FRAP values of approximately 5762 μM Fe(II)/L and DPPH inhibition of up to 79% [30]. Fermentation can also modify phenolic composition and bioaccessibility, indicating that the antioxidant properties of the final product depend on the original matrix, the microorganisms involved, and the processing conditions [46]. It is important to note that analytical antioxidant capacity should not be interpreted as direct evidence of physiological antioxidant efficacy.

Redox regulation is closely interconnected with inflammatory signalling, particularly through redox-sensitive pathways such as NF-κB. Activating Nrf2 and restoring redox homeostasis can counteract inflammatory signalling. Meanwhile, beetroot betalains have been reported to affect the NF-κB, cyclooxygenase-2 (COX-2) and lipoxygenase (LOX) pathways [48,171,172]. Further experimental studies indicate that beetroot betalains can reduce NF-κB DNA-binding activity and the production of inflammatory cytokines, including TNF-α and IL-6 [172,173]. These findings suggest the existence of a connected redox-inflammatory network rather than an isolated antioxidant effect.

While evidence is stronger for conventional than fermented beetroot, human studies provide some support for anti-inflammatory effects. For example, short-term consumption of raw beetroot juice improved systemic inflammatory status in individuals with hypertension [174]. In a 12-week randomised clinical trial involving patients with type 2 diabetes, concentrated beetroot juice reduced circulating IL-6, TNF-α and NF-κB; however, malondialdehyde and high-sensitivity C-reactive protein levels did not differ significantly from those of the control group [175]. Therefore, it appears that beetroot influences certain inflammatory pathways without producing a uniform improvement in all oxidative or inflammatory biomarkers.

The relevance of these mechanisms to fermented beetroot remains unclear. Most evidence for anti-inflammatory activity mediated by betalains derives from purified compounds, extracts, or experimental models rather than fermented beetroot itself [48,68]. Fermentation could modify this activity by altering the concentration, chemical form and bioaccessibility of native betalains and phenolic compounds, generating new metabolites such as 5-hydroxymaltol and changing the properties of the matrix [46,170]. However, the direction and magnitude of these effects depend on the microbial culture, fermentation conditions, and food matrix [46].

Potential interactions between nitrate-derived NO signalling and phytochemical-mediated redox regulation provide an additional mechanistic hypothesis. Increased NO availability could influence inflammatory signalling, while phytochemicals such as betalains and phenolic compounds, as well as fermentation-derived metabolites, may modulate Nrf2, NF-κB, and MAPK pathways. These mechanisms could theoretically act together to influence vascular and cellular homeostasis, but there is currently insufficient evidence to demonstrate synergistic effects in humans. Therefore, such interactions should be regarded as a plausible hypothesis rather than an established property of fermented beetroot.

Thus, the redox and inflammatory effects of fermented beetroot are likely to be the result of interactions between nitrate-derived signalling, native phytochemicals, fermentation-derived metabolites and changes to the food matrix, rather than being caused by a single antioxidant mechanism. Fermentation can change the make-up and bioavailability of beetroot compounds, creating new bioactive metabolites that could affect Nrf2/HO-1, NF-κB and MAPK pathways. However, most of the available evidence is based on experiments or non-fermented beetroot, and there is still limited direct human evidence for fermented beetroot.

### 6.3. Interactions with Oral and Gut Microbiota

The microbiota plays an important role in the interaction between fermented beetroot and host physiology, extending beyond the digestion of food-derived compounds. This is particularly evident in the case of dietary nitrate, since the nitrate–nitrite–NO pathway requires oral nitrate-reducing bacteria. Following ingestion, nitrate is concentrated in saliva and converted into nitrite. This means that the composition and metabolic activity of the oral microbiota can influence the subsequent formation of NO in the body and contribute to differences in how individuals respond to nitrate-rich foods [130,176].

The capacity to reduce nitrate in the mouth is influenced by age, habitual diet, oral hygiene, antibiotic exposure, and other host-related factors [177]. Consumption of beetroot juice has been shown to modify the oral microbiome, including nitrate-reducing taxa. Differences in oral nitrate reduction have also been associated with the blood pressure response to nitrate supplementation [178,179,180]. In a randomised crossover trial involving older adults with treated hypertension, four weeks of consuming nitrate-rich beetroot juice increased the presence of *Neisseria* and decreased the presence of *Veillonella*, without significant changes being observed in the intestinal microbiome [180]. These findings suggest that the oral cavity may be particularly responsive during the initial stages of beetroot-derived nitrate metabolism.

In addition to nitrate metabolism, the gut microbiota provides a second major interface through which beetroot constituents are transformed. Phenolic compounds, betacyanins, dietary fibre and other plant constituents undergo microbial metabolism in the colon, generating metabolites that may differ in properties from their parent compounds [181]. Fermentation before consumption can further modify this process by altering the chemical form and bioaccessibility of compounds that reach the gastrointestinal tract [182]. Therefore, the biological effects of fermented beetroot may be the result of the combined influence of the original constituents, fermentation-derived metabolites and subsequent gastrointestinal microbial transformation.

Experimental evidence illustrates this dynamic interaction [183,184]. The fermentation of beetroot ketchup using *Lactobacillus johnsonii* K4 increased the recovery of several phenolic acids, flavonoids and betalains during simulated digestion, despite the degradation of some polyphenols. Subsequent exposure to gut microbiota further modified the polyphenolic profile, demonstrating that increased release during fermentation does not necessarily result in greater intestinal availability. The pre-existing composition of the gut microbiota may also influence the outcome. In an in vitro faecal fermentation model, red beetroot powder and betanin produced enterotype-dependent changes in microbial composition and SCFA production. The largest increase in SCFAs was observed in microbiota associated with *Bifidobacterium*, compared with *Phocaeicola*- or *Prevotella*-dominated communities [184]. While such models cannot be directly extrapolated to human outcomes, they suggest that beetroot-derived substrates may produce different metabolic responses depending on the host microbiota.

Human evidence remains limited, but it supports the possibility of dynamic gut microbial responses to beetroot. In a 14-day pilot study of healthy adults, beetroot juice was found to temporarily alter the levels of several bacterial species, initially increasing the levels of *Akkermansia muciniphila* and subsequently decreasing the levels of *Bacteroides fragilis*. Betacyanin-derived compounds detected in faeces were associated with specific microbial taxa and increased faecal (iso)butyric acid, suggesting a relationship between beetroot pigment metabolism and microbial SCFA production [183]. These effects appeared to change over time, indicating that microbial responses may adapt during continued consumption.

Fermentation may introduce an additional dimension to these interactions. Lactic acid bacteria convert plant carbohydrates into lactate and other organic acids while also modifying the original plant constituents. The resulting lactate can then be involved in microbial cross-feeding and SCFA production [185]. Studies in animals of lactofermented beetroot juice have reported changes in the composition and metabolic activity of intestinal microbes, including alterations in populations of bacteria associated with the intestinal epithelium and microbial metabolites [153,154]. However, these findings remain predominantly preclinical. Thus, microorganisms act at multiple stages: during fermentation, they modify the beetroot matrix and generate metabolites derived from fermentation, and after consumption, oral and intestinal microorganisms further transform nitrates, phenolic compounds, betacyanins, carbohydrates and microbial products.

Accordingly, the biological activity of fermented beetroot cannot be inferred solely from the concentrations of compounds measured in the original plant. It is likely to depend on the food matrix, fermentation conditions, microbial composition and metabolic capacity, and host characteristics [186]. Human evidence also illustrates the distinction between microbial changes and clinical effects. In adults experiencing the long-term effects of SARS-CoV-2 infection, 14 days of beetroot juice consumption produced favourable changes in several gut microbial groups and inflammatory markers. However, these changes were not accompanied by significant improvements in primary functional outcomes [187]. Therefore, microbiome modulation should not automatically be interpreted as evidence of clinical benefit.

A key unanswered question is whether the effects of these microbes differ between fermented and non-fermented beetroot. While most human studies have examined beetroot juice or powder, direct evidence for fermented products remains scarce. Nevertheless, experimental data suggest that fermentation can alter the recovery and subsequent microbial transformation of beetroot polyphenols [32]. Controlled human studies are therefore required to determine whether fermentation results in distinct oral and gut microbiome responses, either quantitatively or qualitatively, compared with non-fermented beetroot.

Thus, the oral and intestinal microbiota can be considered active participants in the biological processing of fermented beetroot. Fermentation modifies the food matrix prior to ingestion, after which oral and intestinal microorganisms transform nitrates, phenolics, betacyanins, carbohydrates and fermentation-derived metabolites. These processes may influence NO signalling, redox homeostasis, SCFA production, barrier function, and inflammatory pathways [130,176,181,185,186]. However, causal links between specific fermentation-derived metabolites, defined microbial changes, and clinically relevant health outcomes remain insufficiently established.

### 6.4. Potential Contribution of Fermentation-Derived Metabolites

Fermented beetroot should not simply be regarded as beetroot with an altered phytochemical profile, but rather as a dynamically transformed food matrix containing both native compounds and fermentation-derived metabolites [188,189]. Lactic acid bacteria primarily convert carbohydrates into lactic acid, while heterofermentative microorganisms may also produce acetic acid, ethanol and carbon dioxide. This acidification process contributes to microbial stability and modifies the sensory and chemical environment of the product [186]. In fermented beetroot juice, these processes substantially alter the volatile profile, generating a broader range of volatile organic compounds that contribute to preservation and the product’s characteristic sensory properties [189]. Such changes may also influence the stability and transformation of other bioactive constituents.

A major consequence of fermentation is the microbial transformation of plant phenolics. Lactic acid bacteria can hydrolyse glycosidic and ester bonds, modifying hydroxybenzoic and hydroxycinnamic acids and releasing compounds from the plant matrix or converting them into structurally distinct metabolites with different properties [190]. In beetroot, however, these changes appear to be selective rather than uniformly beneficial. Starowicz et al. (2024) [156] reported that, although fermentation reduced total phenolic content, it increased the relative predominance of syringic acid and epicatechin, and that fermented samples exhibited anti-advanced glycation end-product activity. While these findings do not establish a causal role for individual compounds, they suggest that qualitative changes in the phenolic profile may be more significant than total phenolic content alone.

Importantly, the consequences of fermentation depend on the food matrix and processing conditions. In fermented beetroot beverages, for example, lactic fermentation increased the concentration of free phenolic compounds in both juice- and purée-based products. However, in vitro bioaccessibility decreased in fermented juice and increased in fermented purée, potentially reflecting interactions between phenolic compounds and fibre [46]. Similarly, fermentation with *Lactobacillus johnsonii* K4 increased the recovery of several phenolic acids, flavonoids, and betalains during simulated digestion, although some compounds were degraded [32]. Therefore, fermentation should not be considered a universal method of enhancing phytochemical bioavailability. Rather, it can redistribute compounds into free, bound, transformed and degraded forms, depending on microbial metabolism, matrix characteristics and gastrointestinal conditions.

This principle is also relevant to betalains, which are characteristic beetroot pigments that are susceptible to processing. Fermentation can alter the profile and accessibility of individual derivatives, which may affect their stability, antioxidant activity and fate during digestion. Therefore, changes in antioxidant activity cannot be interpreted based solely on total concentrations of betalains or phenolic compounds, as qualitative and matrix-dependent changes may be more significant [46,156].

Fermentation may also produce metabolites that are not present in fresh beetroot. One example is 5-hydroxymaltol, which was isolated from beetroot fermented by *Lactobacillus rhamnosus*. In RAW 264.7 macrophages, purified 5-hydroxymaltol was found to reduce the production of nitric oxide and reactive oxygen species induced by lipopolysaccharide, as well as attenuating NF-κB and MAPK signalling and increasing Nrf2/HO-1 expression [170]. These findings provide experimental support for the potential contribution of fermentation-derived metabolites to redox and inflammatory regulation, although their relevance to human physiology remains to be established.

Fermentation can also broaden the functional profile of beetroot through proteolysis. Microbial proteases generate peptides and free amino acids, some of which may possess antioxidant or antimicrobial properties. Lactic fermentation has also been associated more generally with the formation or release of such bioactive compounds [188]. However, the contribution of these compounds to fermented beetroot remains poorly characterised, and the peptides identified in the food may undergo further hydrolysis during gastrointestinal digestion, which limits the ability to extrapolate directly from food composition to systemic effects.

Another potential contribution is the synthesis of vitamins by microbes. Some lactic acid bacteria can produce B-group vitamins, including riboflavin and niacin, although this ability varies greatly depending on the strain and conditions [191,192]. *Lactococcus lactis* and *Weissella cibaria*, which were isolated from pickled beetroot, were shown to produce certain B vitamins and lactic acid under the right conditions. However, there was substantial variation according to strain, temperature and growth medium [120]. While these observations suggest a possible role for fermented beetroot in microbial biofortification, they do not demonstrate that commercial fermented products contain nutritionally significant amounts of these vitamins.

Thus, fermentation-derived metabolites may contribute to the biological properties of fermented beetroot by acidifying the matrix, transforming phenolics and betalains, forming peptides and other low-molecular-weight metabolites, and, in selected microorganisms, synthesising B-group vitamins. At the same time, however, fermentation can degrade individual phytochemicals, and both native and newly formed compounds may undergo further transformation during digestion. The functional potential of fermented beetroot therefore reflects the interaction between its original constituents, fermentation-derived metabolites, the food matrix and gastrointestinal processes, rather than an increase in any single compound class.

## 7. Biological and Health Effects: Evidence from Experimental and Human Studies

The biological effects of beetroot have been investigated in a wide range of experimental and clinical settings. The most consistent evidence relates to vascular function, blood pressure regulation and certain aspects of exercise physiology, particularly in relation to nitrate-rich beetroot products and their role in the nitrate–nitrite–NO pathway [11,65,161]. Meta-analyses of randomised controlled trials suggest that beetroot juice supplementation may improve endothelial function and lower systolic blood pressure. However, the extent of these effects appears to depend on the population studied, nitrate exposure, intervention duration and the formulation of the beetroot product [14,15,161]. There is also accumulating evidence regarding potential metabolic, antioxidant, anti-inflammatory, gastrointestinal and exercise-recovery effects. However, these outcomes are generally supported by a more heterogeneous and less extensive evidence base than vascular and blood pressure endpoints [48,193,194].

Importantly, the available evidence is highly heterogeneous with respect to beetroot formulation, nitrate content or dose, duration of supplementation, participant characteristics, baseline health status, and clinical or physiological endpoints [11,14]. Consequently, findings obtained with fresh beetroot, conventional beetroot juice, or other nitrate-rich beetroot preparations should not be directly extrapolated to fermented beetroot products. Fermentation may substantially modify the chemical composition, phenolic profile, antioxidant capacity, organic-acid content, and bioaccessibility of beetroot-derived compounds, and these changes appear to depend on the fermentation conditions and food matrix [30,46,116]. Most of the established human intervention evidence concerning cardiovascular and exercise-related effects has been generated using non-fermented, nitrate-rich beetroot preparations. In contrast, research specifically addressing fermented beetroot remains comparatively limited and is dominated by compositional, in vitro, and experimental studies, with only a small number of human investigations available to date [30,46,116].

### 7.1. Cardiovascular Effects

Early clinical studies provided direct evidence that beetroot, which is rich in nitrates, can acutely influence cardiovascular function. In healthy volunteers, beetroot juice was found to reduce blood pressure approximately three hours after ingestion, with this effect being related to an increase in circulating nitrite. The intervention also attenuated endothelial dysfunction and platelet aggregation; however, disruption of the enterosalivary nitrate–nitrite pathway abolished these effects. These findings established dietary nitrate from beetroot as a physiologically relevant source of nitric oxide (NO) and emphasised the vital role of oral microbial nitrate reduction [195]. Subsequent studies have generally supported these effects, although their magnitude and consistency vary depending on the population, nitrate dose, duration of the intervention and baseline vascular status [160,196].

Meta-analyses indicate that the blood-pressure-lowering effect of nitrate-rich beetroot is statistically significant but modest. An early systematic review reported a reduction in systolic blood pressure of approximately 4.4 mmHg, with no significant effect on diastolic pressure [65]. A more recent meta-analysis found mean reductions of approximately 3.6 mmHg for systolic blood pressure and 1.3 mmHg for diastolic blood pressure, with greater effects being associated with longer interventions and higher beetroot juice intake [14]. Evidence from patients with arterial hypertension also supports the significant blood pressure-lowering effect of beetroot-derived nitrate, suggesting its potential value as a complementary nutritional strategy [15]. Nevertheless, these changes should be regarded as modest physiological effects rather than evidence that beetroot can substitute for established antihypertensive therapy.

The cardiovascular effects may also extend beyond blood pressure. Endothelial dysfunction is an important early indicator of cardiovascular disease and is closely linked to reduced NO bioavailability [197]. Studies using flow-mediated dilation (FMD) as an intervention suggest that nitrate supplementation may improve NO-dependent endothelial function. In patients with hypercholesterolemia, long-term nitrate supplementation increased FMD and produced modest improvements in arterial stiffness and platelet activation [198]. Similarly, beetroot juice supplementation in older adults increased FMD and reduced systolic and diastolic blood pressure, though the effects on microvascular function were inconsistent [199]. Therefore, the vascular effects of beetroot appear to involve several related processes rather than blood pressure reduction alone.

However, these responses are not uniform across individuals. An increase in circulating nitrates or nitrites does not necessarily result in measurable improvements in blood pressure, arterial stiffness or endothelial function. In older adults with treated hypertension, four weeks of nitrate-rich beetroot juice increased nitrate exposure and altered nitrate metabolism, but did not improve blood pressure or vascular function compared to nitrate-depleted beetroot juice [180]. This suggests that, although important, nitrate availability may be insufficient to produce a clinically detectable response. Age-related vascular dysfunction, medication use, baseline endothelial status, metabolic health, dietary factors and individual nitrate-reducing capacity may all contribute to this variability [200,201].

This heterogeneity is particularly relevant when considering fermented beetroot. While most cardiovascular research focuses on fresh beetroot, beetroot juice, powder, and other conventional nitrate-rich preparations, direct studies of fermented beetroot are scarce. Fermentation can alter the composition of phenolic compounds, antioxidant activity and the bioaccessibility of phenolic compounds in a matrix-dependent manner [46], and fermentation of red beetroot juice also changes its volatile profile [189]. While these findings demonstrate that fermentation modifies the chemical matrix, they do not establish a cardiovascular benefit.

Thus, conventional beetroot provides biologically plausible evidence that nitrate-derived NO signalling can influence vascular tone and, in some populations, modestly improve blood pressure and endothelial function. Fermentation could theoretically modify these effects through changes in nitrate–nitrite metabolism, food-matrix properties, phenolic bioaccessibility, redox balance and microbial interactions. However, direct clinical evidence remains insufficient to determine whether fermented beetroot provides cardiovascular benefits that are comparable to, greater than, or different from those of non-fermented beetroot.

### 7.2. Metabolic Effects

In addition to its vascular effects, beetroot may influence glucose and lipid metabolism, insulin sensitivity, oxidative stress and other aspects of metabolic dysfunction. These effects are biologically plausible given that nitrate, betacyanin and phenolic compounds can interact with pathways involved in NO signalling, redox balance, inflammation and energy metabolism [196]. Therefore, the metabolic effects of beetroot are unlikely to be the result of a single constituent, but rather the result of interactions between nitrate–nitrite–NO metabolism and the broader phytochemical profile.

Human studies provide some evidence of effects on glucose regulation, although responses are variable. In healthy volunteers, drinking 270 mL of beetroot juice with a carbohydrate-rich meal reduced and delayed postprandial glucose responses compared to a matched control, suggesting an effect on glucose absorption rather than overall glycaemia [202]. In patients with type 2 diabetes, consuming 100 g of raw red beetroot daily for eight weeks was associated with reductions in fasting glucose, HbA1c, ApoB100, liver enzymes, homocysteine and blood pressure, as well as increased antioxidant capacity and improved cognitive performance [203]. While these findings suggest potential metabolic benefits, their generalisability remains uncertain.

The nitrate–nitrite–NO pathway may contribute to glucose regulation by affecting glucose delivery, pancreatic function and insulin-mediated glucose uptake. In obese participants, consuming beetroot juice with glucose improved insulin sensitivity when the oral bacteria that reduce nitrates were intact; however, antibacterial mouthwash attenuated the response [204]. This establishes a link between nitrate metabolism, oral microbiota and metabolic regulation. However, not all studies have demonstrated improvements in glucose- or insulin-related outcomes. Differences in dose, duration, nitrate content, diet, and metabolic status may contribute to inconsistent findings. Therefore, a review of beetroot and cardiometabolic health concluded that evidence for clinically meaningful improvements in glucose and insulin responses remains insufficient [196].

Preclinical findings provide additional biological plausibility. Betanin has been shown to improve fasting glucose, glucose tolerance, and selected lipid and hepatic parameters in models of experimental diabetes. In streptozotocin-induced diabetic rats, these effects were accompanied by increased AMPK and SIRT1 expression and reduced NF-κB expression, suggesting interactions between metabolic and inflammatory pathways [205]. Other experimental studies indicate that beetroot phytochemicals and betalains may affect lipid accumulation and oxidation, and that nitrate-derived NO signalling may influence metabolic function too [206,207,208]. However, these findings cannot be equated with clinical efficacy. Indeed, a meta-analysis of randomised controlled trials in humans found no significant effect of beetroot consumption on total cholesterol, triglycerides, HDL-C or LDL-C, providing limited support for a specific lipid-lowering effect [209]. The hepatic effects are also of interest because insulin resistance, dyslipidaemia, oxidative stress and inflammation all contribute to metabolic dysfunction and non-alcoholic fatty liver disease (NAFLD). In patients with NAFLD, supplementation with *B. vulgaris* alongside standard treatment improved fasting glucose, LDL and HDL cholesterol, and selected liver enzymes [210]. However, as beetroot was administered alongside standard therapy, its independent contribution cannot be determined.

Fermentation may modify these metabolic effects by altering the chemical composition and bioaccessibility of beetroot constituents. The fermentation of beetroot juice using various lactic acid bacteria has been demonstrated to alter the profiles of both the betalains and polyphenols, including the formation of isobetanin and neobetanin. These preparations have been shown to exhibit antioxidant and anti-glycation activity in vitro [27]. Similarly, fermented beetroot has been shown to inhibit AGE formation in two in vitro models, although fermentation has also been found to reduce total phenolic and flavonoid content, producing a distinct profile of individual compounds [156]. These findings emphasise that fermentation does not necessarily enhance bioactivity overall; its effects depend on the microorganism, substrate, product matrix and processing conditions.

Thus, the available evidence supports the metabolic potential of conventional beetroot and suggests that fermented products should be investigated as sources of modified nitrates, betalains, phenolic compounds and microbial metabolites. However, the current evidence base does not demonstrate that fermentation enhances glycaemic control, insulin sensitivity, lipid metabolism or outcomes for metabolic diseases. Direct comparisons of well-characterised fermented and non-fermented beetroot products using standardised fermentation conditions and physiologically relevant doses are therefore needed to determine whether fermentation provides a distinct metabolic advantage.

### 7.3. Exercise-Related Effects

Beetroot has attracted considerable interest in the field of sports nutrition due to its status as a natural source of inorganic nitrate [211,212]. Increased NO bioavailability can affect vascular regulation, skeletal muscle function and oxygen utilisation, which could reduce the physiological cost of exercise [213,214]. The most consistent finding is a reduction in oxygen consumption during submaximal exercise, which indicates improved exercise economy [213]. Meta-analytic evidence further suggests improvements in time to exhaustion, distance covered, muscular endurance, peak power and time to peak power, although these effects are generally modest and not observed across all outcomes. An umbrella review also highlighted substantial methodological heterogeneity in supplementation protocols, exercise models, and study quality [19].

Accordingly, beetroot should not be considered a universally effective ergogenic aid, as responses depend on the characteristics of the exercise performed. In repeated short-duration, high-intensity exercise, for example, beetroot juice increased mean power output during 6 s sprints, but no significant benefit was observed during longer 30 or 60 s efforts [215]. Similar effects have been reported in some intermittent exercise studies, although findings remain inconsistent [215,216]. Proposed explanations include improved muscle perfusion, more efficient contractile function and faster phosphocreatine recovery, with possible effects on calcium handling. However, these mechanisms remain incompletely established in humans [213,214].

Importantly, improved physiological efficiency does not necessarily result in significant performance improvements. For example, reductions in oxygen consumption at a given workload may occur without faster time-trial performance, greater strength or a competitive advantage. Furthermore, systematic reviews suggest that the effects of nitrates are more consistent for certain measures of exercise capacity than for conventional time trials [19,217]. Training status also modifies the response. Recreationally active individuals and those with less training often show greater physiological benefits than highly trained athletes, although some athletes may respond during specific high-intensity or intermittent tasks. Therefore, training status should be regarded as a moderator rather than a simple determinant of response [213,214,218,219].

The form of nitrate supplementation may also influence outcomes. For example, beetroot juice may produce different physiological effects to those of isolated nitrate salts, potentially because its constituents, such as polyphenols and betalains, interact with nitrate-dependent pathways. Comparative evidence suggests that beetroot juice has a greater effect on oxygen consumption during high-intensity exercise, training adaptations and recovery from muscle damage in certain situations. However, differences in nitrate dosage and bioavailability make comparisons difficult, and the current evidence base does not conclusively demonstrate the superiority of beetroot juice over an equivalent nitrate salt [212].

Beetroot may also aid recovery after intense exercise. Controlled studies have reported reduced muscle soreness and improved recovery of selected functional measures following eccentric or repeated sprint exercise [193,220,221]. Meta-analyses indicate improvements in isometric strength recovery, countermovement jump performance and pressure-pain threshold following exercise-induced muscle damage. However, these effects are not consistently accompanied by changes in inflammatory or muscle-damage biomarkers [194,222,223]. This suggests that functional recovery may improve without a clearly established biochemical mechanism. Nitrate supplementation may also provide minor benefits to muscular endurance and, to a lesser extent, strength, whilst reducing the loss of exercise tolerance that occurs after blood donation [217,224]. These findings suggest that the effects of nitrate may be more apparent when oxygen delivery or utilisation is physiologically constrained.

Against this background, fermentation represents a potentially important but largely unexplored variable in beetroot-based sports nutrition. Fermentation can alter the matrix, stability, and bioaccessibility of nitrates, betacyanins, and phenolic compounds, while also generating microbial metabolites and modifying interactions with the microbiota. As oral nitrate reduction is a key step in NO formation, changes in the food matrix or microbial environment could theoretically influence nitrate metabolism. However, it remains unclear whether fermentation enhances this pathway sufficiently to impact exercise performance or recovery.

Thus, there is currently insufficient human evidence to conclude that fermented beetroot provides additional ergogenic or recovery benefits compared to an equivalent non-fermented preparation. Future randomised trials should directly compare fermented and non-fermented products with equivalent nitrate doses and well-defined fermentation conditions. These trials should also assess nitrate–nitrite concentrations, betacyanin and phenolic profiles, microbiota, plasma nitrate–nitrite kinetics and relevant exercise outcomes.

### 7.4. Anti-Cancer Effects

The potential anti-cancer properties of beetroot are of interest due to its phenolic compounds, flavonoids and betacyanins. Experimental evidence suggests that these constituents may influence processes involved in carcinogenesis, including cell proliferation, apoptosis, oxidative stress, inflammation, cell-cycle regulation and angiogenesis in some models [90,116,225]. However, as the evidence is predominantly preclinical, beetroot should be considered a potential source of chemopreventive compounds rather than an established anticancer treatment [226]. Among beetroot constituents, the best characterised in cancer models are the pigments known as betalains, particularly betanin. Their effects appear to involve several pathways that regulate cancer cell survival [22,226,227]. In breast cancer cells, betanin/isobetanin-enriched beetroot extract has been shown to reduce proliferation and viability, activate intrinsic and extrinsic apoptotic pathways, and induce changes in apoptosis-related proteins and mitochondrial membrane potential. Autophagy may also contribute to this response [228]. Similar pro-apoptotic effects of betanin have been reported in colorectal cancer models, including increased expression of BAD, FAS-R and caspases, as well as reduced cancer cell survival [227].

These findings are relevant to fermented beetroot, as the fermentation process can modify the phytochemical profile and availability of individual metabolites. Kazimierczak et al. (2014) [116] demonstrated that naturally fermented beetroot juices could be metabolically distinguished according to production conditions, with juice from organically produced beetroot exhibiting stronger anti-cancer properties than the conventional equivalent. Further evidence comes from lacto-fermented beetroot juice, where products fermented with *Lactobacillus brevis* 0944 and *Lactobacillus paracasei* 0920 reduced carcinogen-induced aberrant crypt foci in the colons of rats. Changes in intestinal microbial conditions and antioxidant status associated with this suggest that potential protection may involve mechanisms beyond direct cytotoxicity [153,229].

However, a major limitation is the discrepancy between the activity observed in experimental models and exposure achievable through normal dietary consumption. Betalains have relatively limited systemic bioavailability: following the consumption of red beetroot juice, only around 0.28% of the ingested intact betanin and isobetanin was recovered in the urine [79,230]. Subsequent studies have also reported low urinary recovery of betacyanins, with substantial variation between individuals and depending on the food matrix [231]. Therefore, concentrations that produce cytotoxic effects in vitro may not be achievable through conventional beetroot intake.

This limitation is particularly important in the case of fermented beetroot. The fermentation process can affect the release, stability, transformation and intestinal metabolism of phytochemicals such as betalains, and may generate additional metabolites with biological activity. However, there is currently insufficient human evidence to determine whether these changes increase systemic exposure or result in clinically relevant anti-cancer effects. Future research should therefore prioritise well-characterised fermented products, standardised fermentation conditions, and the pharmacokinetic assessment of betacyanins and fermentation-derived metabolites.

### 7.5. Other Potential Applications

In addition to its potential anti-cancer properties, beetroot has attracted interest due to its possible effects on metabolic regulation, neurovascular function, tissue protection, microbial ecology and cellular resilience. The latter may be of particular relevance to fermented beetroot, given that fermentation can modify the food matrix, phenolic accessibility and the formation of biologically active metabolites. For instance, the lactic fermentation of beetroot beverages has been shown to increase the levels of free phenolic compounds and antioxidant activity; however, the extent and direction of these changes depend on the matrix and fermentation conditions [46]. Fermentation may also modify the metabolic potential of beetroot. Fermentation with *Latilactobacillus curvatus* PN39 produced strong in vitro inhibition of DPP-IV and α-glucosidase, reaching 87.45% and approximately 80.5%, respectively, and generated dihydromyricetin, which was absent from unfermented beetroot [232]. These findings suggest that fermentation can produce a metabolically distinct matrix; however, enzyme inhibition in vitro cannot be equated with antidiabetic efficacy in humans.

This metabolic potential is closely linked to the interactions between beetroot and microbiota. Beetroot contains nitrates, polyphenols, dietary fibre, fermentable carbohydrates and betaine, all of which can influence the composition of the microbiota and the production of metabolites. Studies in humans and animals suggest that consuming beetroot may affect nitrate-responsive oral bacteria, thereby influencing nitrate reduction and NO availability [233,234]. In the intestine, fermentable carbohydrates and polyphenols may contribute to the production of SCFAs, including butyrate, which supports the integrity of the epithelial barrier and immune regulation [235,236]. In healthy adults, 14 days of beetroot juice consumption altered the abundance of several bacterial taxa, including an increase in *Akkermansia muciniphila*, and betacyanin-derived metabolites were associated with these microbial changes and increased faecal (iso)butyric acid levels [183]. Similar microbial and metabolite changes were observed in adults with the long-term effects of SARS-CoV-2 infection. However, the short intervention and clinical heterogeneity limit conclusions regarding therapeutic relevance [237]. These findings are particularly relevant to fermented beetroot, as the presence of microorganisms and fermentation-derived metabolites prior to consumption may modify subsequent host–microbiome interactions.

Another potential application relates to neurovascular and cognitive function. Dietary nitrate can improve NO-dependent vascular responsiveness and cerebral perfusion, suggesting a possible connection between nitrate-rich beetroot and cognitive function [238,239]. A single dose of nitrate-rich beetroot juice has been shown to alter prefrontal cortical haemodynamic responses and improve performance in one cognitive task, while a high-nitrate diet containing beetroot juice has been shown to increase regional cerebral perfusion in older adults [239,240]. However, cognitive outcomes have been inconsistent. For example, beetroot juice improved simple reaction time in older adults with type 2 diabetes, but did not improve other cognitive measures [241]. Other studies have found no cognitive benefit, despite increased plasma nitrite and reduced blood pressure [242], or after 13 weeks of supplementation in overweight and obese older adults [243]. Differences in age, vascular and metabolic status, dose, duration and cognitive assessment may contribute to this variability. Therefore, beetroot cannot currently be considered an established cognitive-enhancing intervention. Furthermore, almost all human research has focused on non-fermented beetroot or isolated nitrate, meaning the neurovascular effects of fermented beetroot remain largely unexplored. Fermentation may also modify the accessibility and bioaccessibility of phenolic compounds, which could interact with nitrate-dependent vascular signalling [46].

The potential neuroprotective properties of beetroot may be attributed to its betalains and phenolic compounds, which have been shown to modulate oxidative stress and inflammatory signalling in experimental models [244,245]. Fermentation may further alter these compounds in a matrix-dependent manner [46]. However, direct neuroprotection is currently supported mainly by cellular and animal studies, while research in humans has primarily focused on cerebrovascular and cognitive outcomes following the consumption of non-fermented products [239,244]. Long-term studies, particularly of fermented beetroot, are therefore required to establish clinical relevance.

Beetroot also has potential antimicrobial applications. Its phenolic compounds and betacyanins have been investigated for use in functional foods, antimicrobial formulations and active food systems [246,247]. Beetroot extracts can inhibit various microorganisms in vitro, but the extent of this inhibition depends strongly on the cultivar, the extraction conditions, the concentration, the microbial strain and the experimental design [248,249,250]. There have been reports of antibacterial effects against *Bacillus cereus* and *Salmonella typhimurium*, including effects on bacterial physiology and apoptosis-like cell death [251,252]. As most of the evidence is currently in vitro and the experimental conditions vary substantially, it may be more realistic to consider near-term applications in functional foods, antimicrobial packaging and other active food systems than in therapeutic antimicrobial preparations.

Table 3 summarises the current evidence for the biological and health-related effects of beetroot-derived products, categorised by study design, population, product characteristics, fermentation status and level of evidence.

Thus, the potential applications of fermented beetroot extend beyond antioxidant and cardiovascular effects to include metabolic regulation, modulation of the microbiome, neurovascular function, tissue protection and antimicrobial properties. However, the strength of the evidence varies considerably. Most findings are derived from in vitro and animal studies, and human evidence is generally limited to short-term interventions and specific physiological endpoints. Therefore, fermented beetroot should currently be considered a promising functional food rather than an established therapeutic intervention. Future research should focus on well-characterised fermentation protocols, dose–response relationships, fermentation-derived metabolites, bioavailability, microbiome-dependent mechanisms and adequately powered randomised trials, in order to determine whether fermentation-related biochemical changes translate into meaningful health benefits for humans.

## 8. Safety, Quality and Standardisation

The potential for fermented beetroot to be used as a functional food depends on various factors, including its phytochemical composition and biological activity, as well as the safety, reproducibility and stability of the final product. It is important to note that “fermented beetroot” does not represent a single standardised product. Its composition can vary depending on the cultivar, maturity, agricultural conditions, indigenous microbiota, starter culture, fermentation temperature and duration, salt concentration, oxygen exposure and storage conditions. This variability is particularly relevant when biological effects are reported without a detailed characterisation of the administered product. Studies of fermented vegetables confirm that nutritional and chemical changes depend strongly on the raw material, the microbial community, the fermentation conditions and the post-fermentation processing [258].

From a technological perspective, controlled lactic acid fermentation generally offers greater reproducibility than spontaneous fermentation. The rapid establishment of lactic acid bacteria and reduction in pH can limit the growth of unwanted microorganisms and improve process control. In fermented red beetroot, for example, starter cultures have been shown to modify free amino acids, biogenic amines, antioxidant properties and the composition of betalains, with the extent of the modification varying according to the bacterial strain and the cultivar of the beetroot [26]. Thus, while defined starter cultures can reduce variability, they cannot eliminate it. Product safety also depends on the quality of the raw material, hygiene, the temperature and duration of fermentation, pH development, salt concentration, packaging, oxygen exposure and storage. Across fermented vegetables, microbial safety, nitrite accumulation and biogenic amines remain important quality control concerns [258].

Among these, biogenic amines are an important chemical safety concern. Histamine, tyramine, putrescine and related compounds can be produced by microorganisms with decarboxylase activity, and this property tends to be strain-specific rather than universal to a species or genus [259,260,261]. In fermented red beetroot, for example, the selection of the starter culture influences the formation of biogenic amines [26], which supports the use of cultures with low aminogenic potential. Therefore, fermentation should not be assumed to reduce biogenic amines. Rather, strain selection, process control and analytical monitoring, where appropriate, should be incorporated into safety assessments, particularly for products intended for regular consumption.

Nitrate and nitrite also require careful interpretation. Beetroot is naturally nitrate-rich, whereas nitrite levels may increase through microbial nitrate reduction during fermentation. These changes must be distinguished from the enterosalivary conversion of nitrate after consumption and subsequent systemic nitrate/nitrite metabolism. Consequently, changes in food nitrate or nitrite concentrations alone cannot be interpreted as evidence of improved or impaired physiological nitrate utilisation. The biological effects of dietary nitrate depend on the sequential nitrate–nitrite–NO pathways, as well as the food matrix and reaction conditions [262]. Current evidence does not prove that fermentation consistently increases the bioavailability of nitrates or nitrites compared to nitrate-matched non-fermented beetroot. Therefore, food nitrate and nitrite concentrations, plasma kinetics and NO-related biomarkers should be considered distinct stages of nitrate metabolism.

Another consideration is the potential formation of N-nitroso compounds, particularly where nitrite accumulates. The nitrosation process is influenced by various factors, including pH, nitrite concentration, temperature and substrate availability [263,264]. However, dietary nitrate or nitrite should not automatically be equated with toxicological risk since vegetable-derived nitrate occurs within a complex matrix containing antioxidants and other compounds that may influence nitrosation and nitrate metabolism [265]. There is limited data specifically quantifying N-nitroso compounds in fermented beetroot, which supports a product-specific rather than generalised safety assessment.

Salt concentration also affects technological performance and nutritional quality. It influences microbial selection, acidification, texture, pigment stability, and storage; however, excessive sodium may reduce the suitability of fermented beetroot for regular consumption. In the lactic fermentation of red beetroot, salt concentration affects pH, acidity, microbial growth, pigment content, colour and physicochemical properties. Lower salt levels may allow for reduced sodium content without compromising key fermentation characteristics [121]. Salt should therefore be considered as a technological, microbiological and nutritional parameter simultaneously.

Product stability is also strongly influenced by the preservation of betalaine pigments. Betacyanins and betaxanthins are sensitive to temperature, pH, oxygen, light, water activity and storage conditions [49,142]. The effects of fermentation are strain-dependent: different lactic acid bacteria can alter the profiles of betalaine and polyphenols, including the formation of isobetanin and neobetanin, as well as changes in flavonoids [27]. Therefore, fermentation may preserve, transform, or degrade individual compounds depending on the processing conditions. Consequently, an increase in antioxidant capacity should not necessarily be taken as evidence of greater stability of betalauns, as antioxidant activity may also reflect the release of matrix-bound phenolic compounds, phytochemical transformation or the formation of fermentation-derived metabolites.

These findings highlight the need for broader product standardisation. The composition of fermented beetroot may be influenced by cultivar, geographical and agricultural conditions, maturity, nitrate fertilisation, pre-fermentation storage, particle size, sugar availability, inoculum size, microbial strain, temperature, fermentation duration, pH, salt concentration, and post-fermentation processing [258]. Therefore, results obtained with one preparation cannot necessarily be extrapolated to another product described simply as “fermented beetroot”.

Accordingly, standardisation in experimental and clinical studies should cover the source of the raw material or cultivar, the fermentation microorganisms used, the level of inoculum, the temperature and duration of fermentation, the pH trajectory, the salt concentration, the storage conditions and the post-fermentation processing. Chemical characterisation should preferably cover nitrates and nitrites, major betalains, selected phenolic compounds and relevant fermentation-derived metabolites. Where appropriate, this should be supplemented by microbiological characterisation and viable counts. Recent studies illustrate the importance of this approach, as different starter cultures can generate substantially different profiles of betalains, polyphenols, biogenic amines and antioxidant activity [26,27].

These requirements are particularly important when translating experimental findings into clinical applications. Much of the evidence for the cardiovascular effects of beetroot derives from conventional beetroot juice or dietary nitrate, whereas direct clinical evidence for the effects of fermented beetroot remains limited. Therefore, fermentation should not be assumed to reproduce or enhance the physiological effects of conventional beetroot products. Well-characterised fermented and non-fermented preparations should be evaluated in comparative human studies, preferably matched for nitrate content, while assessing plasma nitrate–nitrite kinetics, vascular responses, relevant redox biomarkers, gastrointestinal tolerance and clinically meaningful outcomes.

Thus, fermentation represents a technological opportunity rather than a guarantee of improved safety or quality. Future development should prioritise validated fermentation conditions, microbiological safety, monitoring of nitrate, nitrite, and biogenic amines, phytochemical characterisation, betalain stability, and batch-to-batch reproducibility. Such standardisation is necessary to determine whether fermentation-related compositional changes produce reproducible biological effects in humans rather than simply generating a chemically distinct beetroot product.

## 9. Limitations

Despite the growing interest in fermented beetroot as a source of bioactive compounds, the available evidence is limited, which restricts its clinical application. A major challenge lies in the variable bioaccessibility and bioavailability of phytochemicals, which depend on factors such as the food matrix, processing, fermentation, gastrointestinal digestion and gut microbiota. Phenolic compounds may undergo extensive transformation during digestion and microbial metabolism, meaning that the metabolites present in the bloodstream can differ substantially from the original compounds in the product. Consequently, the biological effects cannot always be directly attributed to the parent compounds measured in fermented beetroot [8,266].

This complexity is further increased by substantial inter-individual variability. Polyphenol absorption and biotransformation are influenced by the composition and activity of the gut microbiota, as well as by age, sex, body composition, physiological or pathological status, genetic factors and lifestyle [266]. Therefore, the same fermented beetroot preparation may produce different metabolic and physiological responses in different individuals, particularly since fermentation introduces an additional biological transformation prior to consumption.

Another limitation is the discrepancy between the concentrations used in experiments and the exposure achieved through normal dietary intake. Much of the evidence for the antioxidant, anti-inflammatory, antimicrobial, cytoprotective and neuroprotective effects of beetroot-derived compounds is based on in vitro and animal studies, which often use concentrations or doses that cannot be achieved through normal consumption. Furthermore, cellular models cannot fully reproduce human digestion, absorption, first-pass metabolism, microbial transformation, tissue distribution and elimination. Therefore, such findings should be regarded primarily as hypothesis-generating rather than as direct evidence of clinical efficacy.

The complex beetroot matrix adds another layer of uncertainty. Nitrates, betalains, phenolic compounds, organic acids, dietary fibre and other constituents may interact during fermentation and digestion. This means that the effects of the whole product may reflect the combined or potentially synergistic actions of multiple compounds rather than those of a single compound [68]. Therefore, it is difficult to determine whether fermentation primarily preserves existing compounds, releases matrix-bound constituents, degrades specific molecules, or generates new metabolites. These matrix-dependent effects also complicate comparisons between isolated compounds, fresh beetroot, conventional beetroot juice and fermented preparations [8].

Interpretation is further limited by the heterogeneity of fermented beetroot products. The outcome of fermentation depends on the microorganisms used, their abundance, the composition of the substrate, temperature and duration of fermentation, oxygen availability, pH, salt concentration and storage conditions. Consequently, products described simply as ‘fermented beetroot’ may differ substantially in their chemical and microbial profiles. Therefore, fermentation should be regarded as a dynamic biological process involving microbial growth and enzymatic transformation, rather than a uniform treatment with predictable consequences [24].

This heterogeneity complicates comparisons between studies, as apparent inconsistencies may reflect differences in cultivar, nitrate and betacyanin content, phenolic composition, fermentation strain and conditions, or storage. Human studies also vary in terms of the dose administered, the duration of the intervention, the comparator used, the dietary background of the participants, their health status, and the outcome measures employed. As fermented preparations are often characterised less comprehensively than conventional beetroot products, such differences limit reproducibility and mechanistic interpretation [68].

Most importantly, the majority of human evidence relates to conventional beetroot juice or dietary nitrate, rather than fermented beetroot. Therefore, the established effects of nitrate-rich beetroot on blood pressure, endothelial function, cerebral perfusion and exercise-related outcomes cannot automatically be attributed to fermentation. Similarly, increased phenolic bioaccessibility or the formation of fermentation-derived metabolites does not demonstrate a clinically meaningful benefit. Well-characterised direct head-to-head studies of fermented and non-fermented beetroot are needed to determine whether fermentation provides an additional physiological advantage.

The clinical evidence is also limited by the small number of long-term, well-controlled human studies. Many investigations are acute or short-term, involve modest sample sizes, or rely on surrogate biomarkers rather than clinically meaningful outcomes. This is particularly limiting with regard to neuroprotection, the prevention of metabolic disease, microbiome-mediated effects and long-term cardiovascular protection, as these require sustained exposure and longitudinal assessment. Changes in microbiota or circulating metabolites should not be interpreted as evidence of a health benefit in the absence of corresponding functional or clinical outcomes.

Therefore, fermented beetroot should be considered a promising, albeit incompletely characterised, functional food rather than a clinically established therapeutic intervention. Future research should combine rigorous product characterisation with controlled fermentation protocols and validated assessment of nitrate–nitrite–NO metabolism. It should also include comprehensive metabolomic and microbiome analyses. Adequately powered human trials should address dose–response relationships, inter-individual variability and the biological roles of fermentation-derived metabolites. This is essential in order to distinguish the effects that are specifically attributable to fermentation from those that arise from the intrinsic composition of beetroot, and to establish whether these changes result in reproducible, clinically meaningful benefits.

## 10. Future Perspectives and Research Directions

Future research on fermented beetroot should move beyond documenting changes in individual phytochemicals, focusing instead on how fermentation-induced transformations translate into biological responses in humans. As fermentation involves interactions between plant compounds, microorganisms and fermentation-derived metabolites, an integrated approach combining metabolomics, microbiome profiling, transcriptomics, proteomics and nutrigenomics may provide a more comprehensive understanding of the underlying biological pathways [267,268].

A key objective should be to identify the metabolites that mediate these effects. Changes in betacyanins, phenolic acids, nitrate-derived compounds and other phytochemicals do not necessarily indicate which molecules remain biologically active after ingestion. Fermentation, gastrointestinal digestion and microbial metabolism may generate more bioavailable or biologically distinct metabolites. Metabolomics can therefore help to identify biomarkers of exposure and response, and distinguish fermentation products from compounds that are mechanistically linked to physiological effects [269,270].

This research framework should integrate the gut microbiome because microbial metabolism influences the transformation of nitrates and phytochemicals, as well as host physiology. Future studies should determine whether specific microbial taxa or functional pathways are associated with differences in nitrate–nitrite–NO metabolism, phenolic metabolism, short-chain fatty acid production and inflammatory or redox signalling. This may help to explain variability in responses to fermented beetroot between individuals [271].

Well-designed, adequately powered, long-term human studies are equally important. Current beetroot research is dominated by short-term or acute interventions, while the long-term effects of consuming fermented beetroot regularly on vascular, metabolic, cognitive and inflammatory health remain unclear. Future trials should use well-characterised products and assess whether changes in NO-related signalling, endothelial function, redox status and microbiome composition are sustained. Populations with impaired endothelial function, reduced NO bioavailability or chronic low-grade inflammation may provide valuable insights into potential benefits.

Direct comparisons between fermented and non-fermented beetroot are essential to establish whether fermentation provides a biological advantage beyond chemical modification. Where feasible, products should be matched for nitrate content, dose, and energy intake, while simultaneously assessing plasma nitrate and nitrite levels, NO-related metabolites, vascular responses, inflammatory and oxidative stress markers, and changes to the oral and intestinal microbiome. Such a design would help to distinguish the effects that are attributable to dietary nitrate from those that are specifically associated with fermentation-derived phenolics, betacyanins, and microbial metabolites.

From a technological perspective, research should progress from empirical fermentation towards reproducible processes based on defined microbial and metabolic characteristics. Optimising starter strains, temperature, duration and substrate conditions could improve consistency and enable the targeted modulation of metabolite production. Integrating multi-omics data with fermentation parameters could facilitate the rational selection of starter cultures and the development of products with consistent chemical and functional profiles [267,268].

The potential health benefits of fermented beetroot should be investigated within a broader functional-food framework. Rather than focusing on individual compounds such as nitrates or betanin, the development of future products could involve combining phytochemical sources, fermentable substrates and selected microorganisms in order to establish whether particular combinations have consistent effects on vascular function, redox homeostasis, intestinal barrier integrity or immune signalling. The concept of fermented foods as complex systems containing both microorganisms and fermentation-derived metabolites is a useful basis for such research [24,272].

The significant variability in individual responses to diet makes fermented beetroot a potential model for precision nutrition. Responses to nitrate, betalains, and fermentation-derived metabolites may be influenced by age, metabolic phenotype, genetic background, habitual diet, and gut microbiota. Integrating microbiome, metabolomic, metabolic, and clinical data could help to identify those most likely to benefit from specific fermented beetroot preparations [269,271].

Thus, advancing fermented beetroot research will require the integration of food science, microbial ecology, metabolomics, and human nutrition, rather than investigating them in isolation. Standardised products, longitudinal clinical studies, mechanistic validation and precision nutrition approaches will be critical to determining whether fermented beetroot can progress from a promising functional food concept to a scientifically supported dietary strategy.

## 11. Conclusions

Over the past two decades, beetroot has emerged as a multidisciplinary research topic spanning vascular physiology, redox biology, microbiome science and food processing. This review highlights the interconnected roles of nitrate–nitrite–NO signalling, betalains, phenolic compounds, and fermentation-derived metabolites in determining its biological potential. These effects may involve multiple interacting pathways, including the interaction of oral and gastrointestinal microbiota with redox signalling, vascular function and host metabolic responses.

A key finding is that fermentation can significantly alter the chemical and functional properties of beetroot. For example, lactic acid fermentation can alter nitrate- and nitrite-related metabolites, transform phenolic compounds and betalains, generate microbial metabolites, and modify the food matrix and bioaccessibility of phytochemicals. However, the extent of these effects depends on the microbial strain, fermentation conditions, raw material and storage. Therefore, fermentation should not be considered inherently beneficial, and increased antioxidant activity or recovery of individual compounds cannot be interpreted as evidence of improved clinical efficacy in isolation.

The microbiome is another component that could be important for the activity of fermented beetroot. Beetroot-derived nitrates, fibres and phytochemicals can influence microbial metabolism, and fermentation introduces additional microbial products that may affect the host’s response after ingestion. While consumption of beetroot has been linked to changes in certain microbial taxa and short-chain fatty acid production, evidence for fermented beetroot remains limited. The likelihood of such effects depends on product composition, dose, duration, habitual diet, and host characteristics, and they should therefore be regarded as promising, albeit not yet established, clinical mechanisms.

The available evidence supports the biological plausibility of beetroot-derived interventions for vascular function, metabolic regulation, redox homeostasis, exercise-related responses and, potentially, cognitive and neurovascular function. However, the strength of the evidence varies substantially. The strongest human evidence is for dietary nitrate from conventional beetroot products, whereas research on fermented beetroot is dominated by food technology studies, in vitro experiments and preclinical models. Consequently, findings obtained using conventional beetroot juice or isolated constituents cannot be directly extrapolated to fermented preparations.

Thus, fermentation is a promising technological strategy for developing functional beetroot-based foods with customised phytochemical and microbial profiles. Its potential value lies in its ability to modify the concentrations of individual bioactive compounds, alter the food matrix, and generate metabolites that may influence host–microbiome interactions. However, it remains to be established in well-controlled human studies whether these compositional changes translate into reproducible improvements in vascular, metabolic, cognitive, or redox-related outcomes. Therefore, fermented beetroot should currently be considered a promising approach to functional foods rather than a clinically established intervention. Future research should focus on standardised products and direct comparative clinical evaluation.

## Figures and Tables

**Figure 1:**
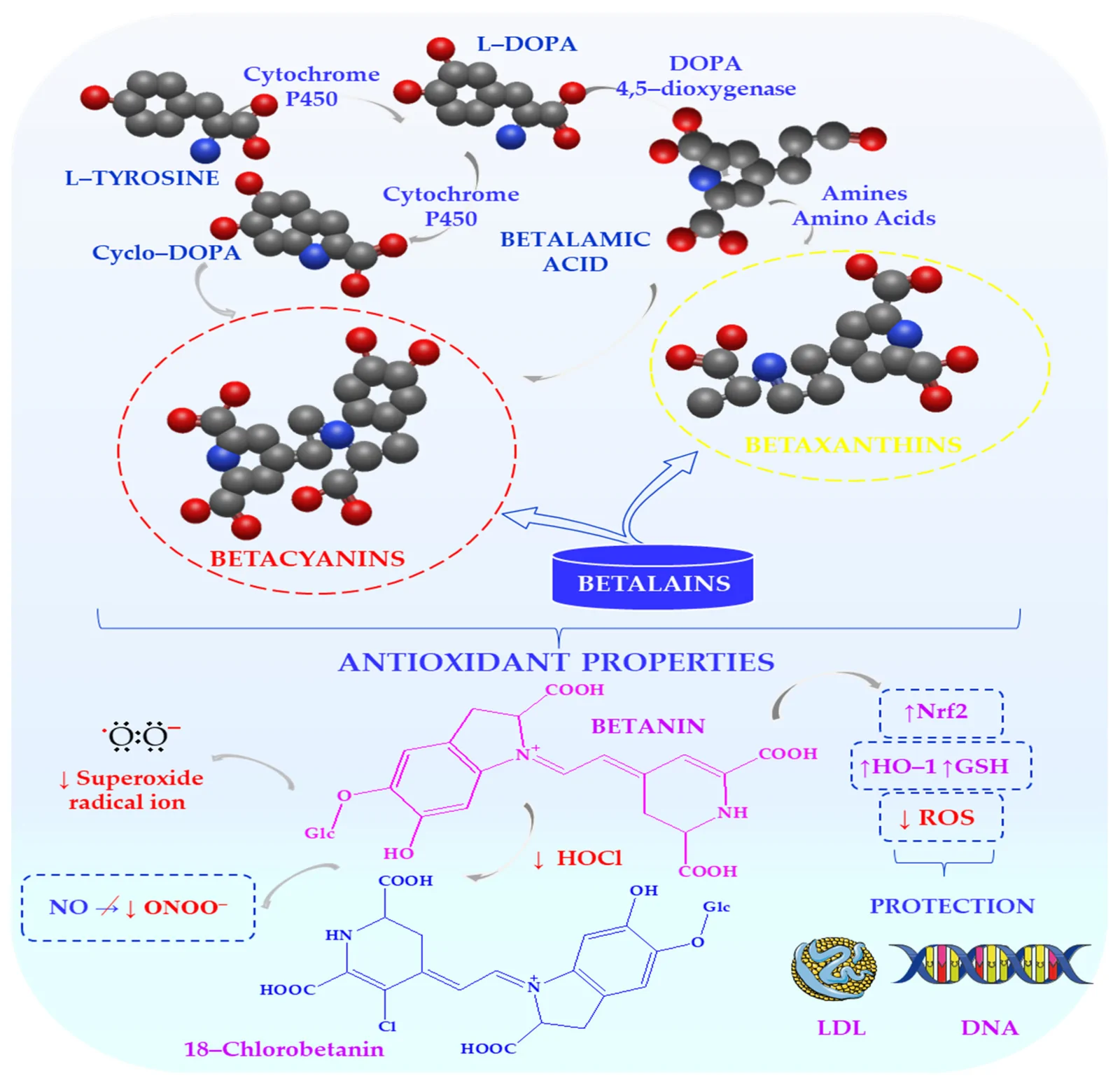
Biosynthesis, redox properties, and anti-inflammatory activity of beetroot betalains. Betalains are biosynthesized from L-tyrosine via L-DOPA and betalamic acid to form betacyanins and betaxanthins, and they participate in the regulation of redox balance. Betalains, particularly betanin, exhibit potent antioxidant activity and the ability to neutralise HOCl. Betanin activates Nrf2-dependent transcription, protecting LDL and DNA from oxidative damage. These mechanisms contribute to endothelial protection, limiting oxidative stress and suppressing inflammatory processes. Image provided by Servier Medical Art (https://smart.servier.com, accessed on 23 June 2026), licenced under CC BY 4.0 (https://creativecommons.org/licences/by/4.0/, accessed on 20 March 2026). Abbreviations: DNA—deoxyribonucleic acid; GSH—glutathione; HO-1—heme oxygenase-1; HOCl—hypochlorous acid; L-DOPA—L-3,4-dihydroxyphenylalanine; LDL—low-density lipoprotein; Nrf2—nuclear factor erythroid 2–related factor 2; ROS—reactive oxygen species.

**Figure 2:**
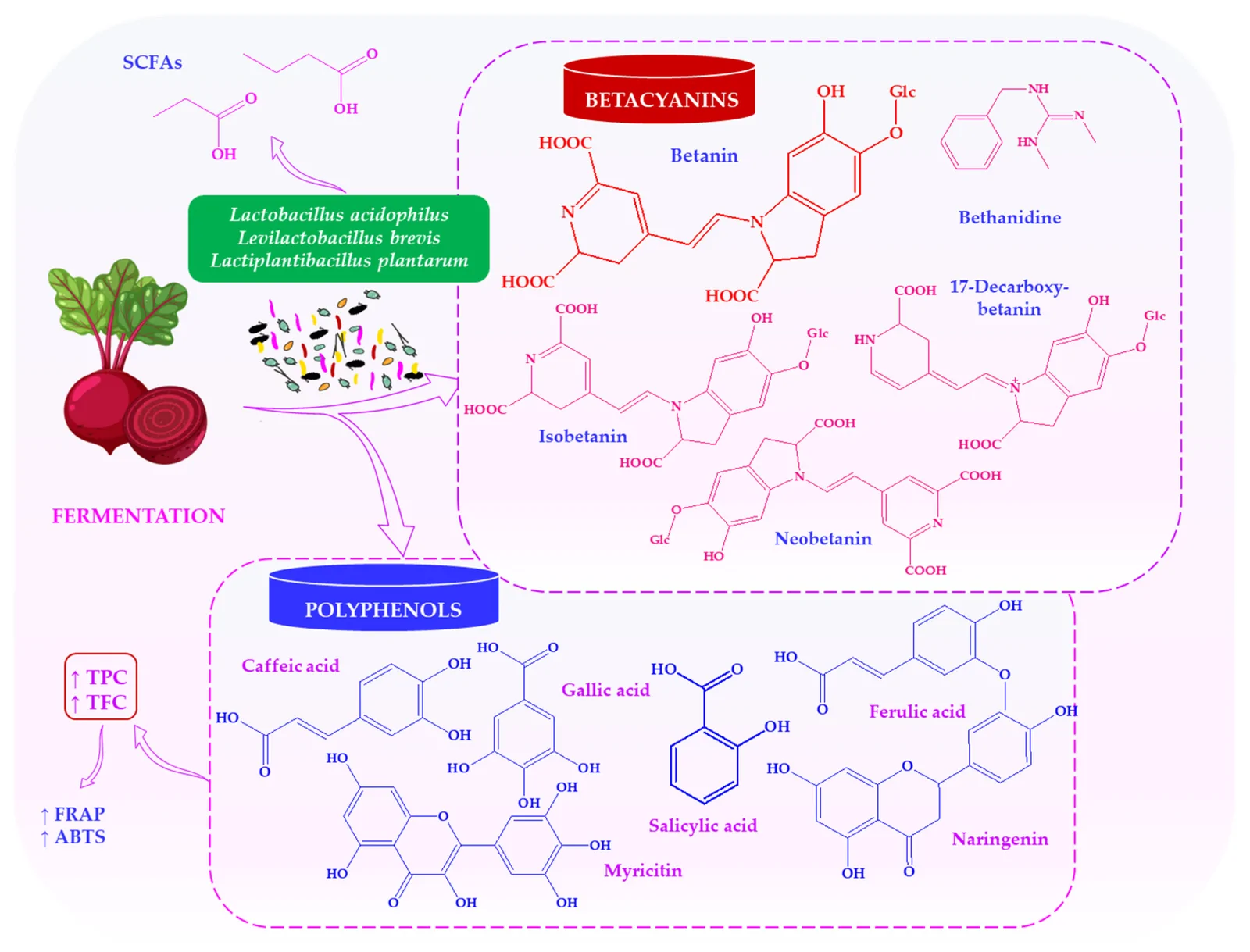
Impact of fermentation on phytochemical transformation and antioxidant capacity in beetroot juice. Lactic acid fermentation remodels the beetroot matrix through microbial and enzymatic transformations. Depending on the microorganism, process conditions and product matrix, fermentation may release, transform or degrade phenolic compounds and betalains, alter nitrate and nitrite concentrations, and generate organic acids and other microbial metabolites. These changes can modify antioxidant and anti-inflammatory potential and produce product-dependent effects on gastrointestinal bioaccessibility; however, improved systemic bioavailability or clinical efficacy cannot be assumed without direct evidence. Short-chain fatty acids may arise subsequently from intestinal microbial metabolism of fermentable substrates and should be distinguished from metabolites formed directly during product fermentation. Image provided by Servier Medical Art (https://smart.servier.com, accessed on 23 June 2026), licenced under CC BY 4.0 (https://creativecommons.org/licences/by/4.0/, accessed on 20 March 2026). Abbreviations: ABTS—2,2′-azino-bis(3-ethylbenzothiazoline-6-sulfonic acid); FRAP—ferric reducing antioxidant power; SCFAs—short-chain fatty acids; TFC—total flavonoid content; TPC—total phenolic content.

**Figure 3:**
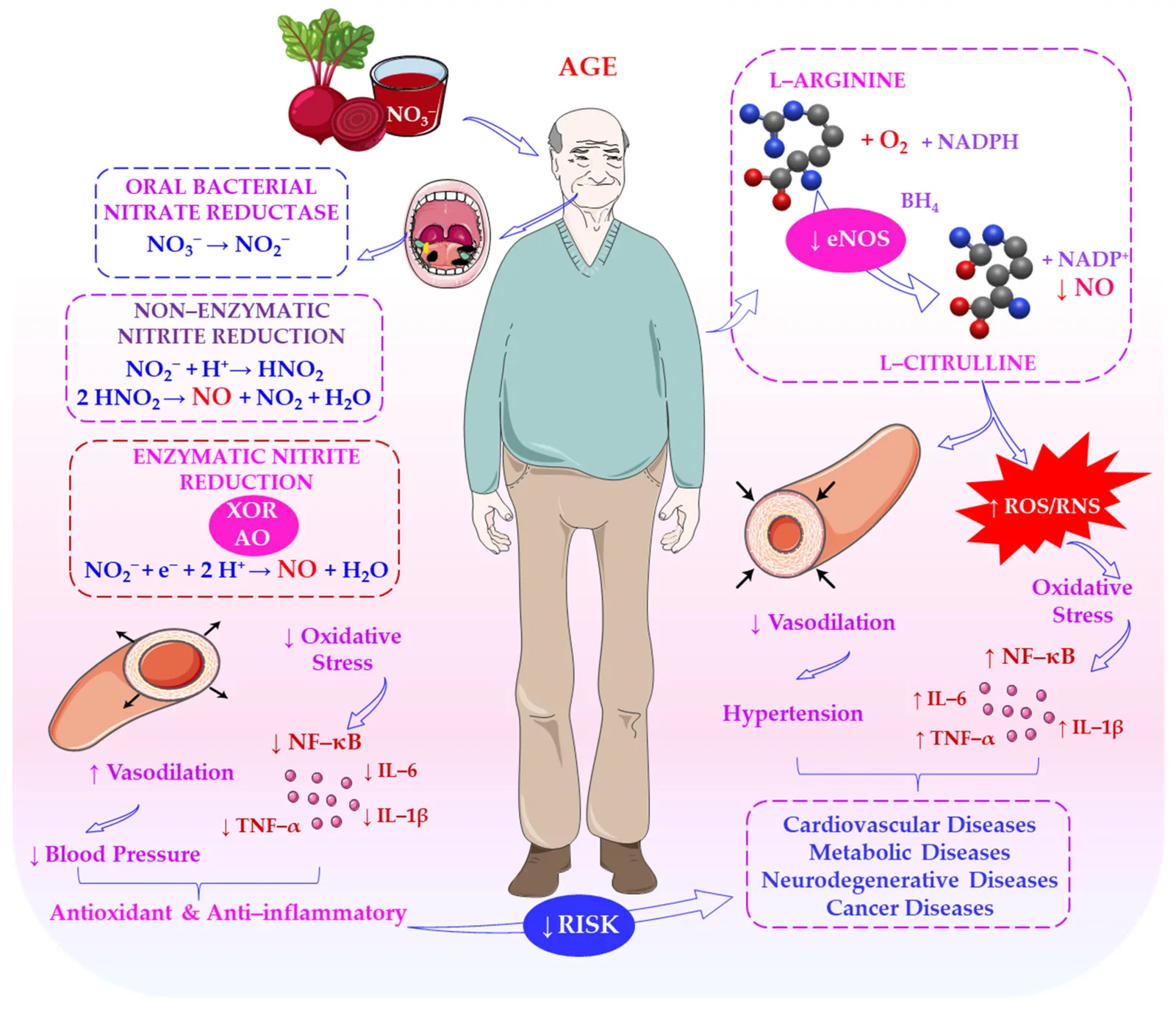
Mechanisms by which beetroot-derived dietary nitrates regulate redox balance and inflammatory signalling. The nitrate–nitrite–NO pathway increases nitric oxide (NO) bioavailability, enhances antioxidant defence systems, reduces ROS accumulation, and suppresses NF-κB activation and pro-inflammatory cytokine production, thereby improving endothelial function and lowering the risk of cardiovascular and other age-related diseases. Image provided by Servier Medical Art (https://smart.servier.com, accessed on 23 June 2026), licenced under CC BY 4.0 (https://creativecommons.org/licences/by/4.0/, accessed on 20 March 2026). Abbreviations: AO—antioxidant activity; BH4—tetrahydrobiopterin; eNOS—endothelial nitric oxide synthase; IL-1β—interleukin-1 beta; IL-6—interleukin-6; NADP^+^—nicotinamide adenine dinucleotide phosphate (oxidised form); NADPH—nicotinamide adenine dinucleotide phosphate (reduced form); NF-κB—nuclear factor kappa B; NO—nitric oxide; RNS—reactive nitrogen species; ROS—reactive oxygen species; TNF-α—tumour necrosis factor alpha; XOR—xanthine oxidoreductase.

**Table 1:** Major bioactive and nutritional constituents of red beetroot (*Beta vulgaris* L.) before fermentation and their potential biological relevance.

Constituent/Compound Group	Representative Compounds	Main Nutritional or Biological Relevance	Factors Affecting Content	References
Nitrate	NO_3_^−^	Precursor of the nitrate–nitrite–NO pathway; contributes to NO bioavailability and vascular regulation	Cultivar, plant tissue, formulation, agronomic and environmental conditions, processing and storage	[54,55,56,59]
Nitrite	NO_2_^−^	Intermediate in nitrate reduction and a direct precursor of NO, particularly under hypoxic or acidic conditions	Oral microbial nitrate reduction, storage, processing and physicochemical conditions of the food matrix	[40,59,61,62]
Betacyanins	Betanin, isobetanin, betanidin and related derivatives	Redox-active pigments with antioxidant, potential anti-inflammatory and cytoprotective activities	Cultivar, plant maturity, growing conditions, pH, temperature, oxygen, light, processing and storage	[25,68,69,74]
Betaxanthins	Vulgaxanthin I and II, indicaxanthin and related pigments	Antioxidant and redox-modulating activity; potential contribution to the overall antioxidant properties of beetroot	Cultivar, maturity, processing temperature, pH, oxygen and storage	[69,73,74]
Betaine	Glycine betaine (trimethylglycine)	Methyl donor and osmoprotective compound; participates in one-carbon metabolism and homocysteine remethylation	Cultivar, plant tissue, environmental conditions and processing	[56,92,99]
Phenolic acids	Gallic, caffeic, ferulic, p-coumaric, chlorogenic, syringic, vanillic and p-hydroxybenzoic acids	Antioxidant and redox-modulating activities; potential effects on inflammatory, metabolic and vascular pathways	Cultivar, plant tissue, maturity, growing conditions, storage and processing	[7,56,68,87]
Flavonoids and other polyphenols	Betavulgarin, betagarin, cochliolin A, dihydroisorhamnetin and other flavonoid derivatives	Potential antioxidant, cytoprotective and anti-inflammatory activities; may modulate redox-sensitive signalling pathways	Cultivar, plant tissue, growing conditions, storage, processing and microbial biotransformation	[7,90,91]
Vitamin C	Ascorbic acid	Water-soluble antioxidant and enzymatic cofactor; contributes to protection against oxidative damage and maintenance of connective tissue	Storage, oxygen exposure and thermal processing	[56,92,96,97]
B-group vitamins	B-vitamins present in beetroot tissues	Participate in energy metabolism, cellular metabolism and metabolic homeostasis	Cultivar, plant tissue, environmental conditions, storage and processing	[7,56,68]
Potassium	K^+^	Essential electrolyte involved in vascular, neuromuscular and cellular function	Cultivar, plant tissue, soil composition and agricultural conditions	[56,100,101]
Magnesium	Mg^2+^	Cofactor in numerous enzymatic and metabolic processes; contributes to neuromuscular and cellular function	Cultivar, plant tissue and growing conditions	[56,100,101]
Iron	Fe	Essential for oxygen transport and multiple cellular and enzymatic processes	Soil composition, cultivar, plant tissue and agricultural conditions	[56,68,102]
Manganese and other trace minerals	Mn and other mineral constituents	Enzyme cofactors involved in cellular metabolism and antioxidant defence	Cultivar, plant tissue, soil composition and growing conditions	[7,56,68]
Dietary fibre	Soluble and insoluble polysaccharides and cell wall components	Supports intestinal function and provides a fermentable substrate for intestinal microorganisms	Plant tissue, maturity and processing	[8,46,52]
Carbohydrates	Sugars and structural polysaccharides	Major nutritional energy substrates and potential substrates for microbial fermentation	Cultivar, plant tissue, maturity and processing	[8,56,92]
Organic acids	Malic, citric, ascorbic, fumaric and oxalic acids; succinic acid in selected products or formulations	Contribute to flavour, acidity, physicochemical properties and the metabolic and redox characteristics of the food matrix	Cultivar, plant material, formulation, maturity and processing	[56,92,93]
Amino acids and nitrogen-containing compounds	Methionine, cysteine, leucine, isoleucine, histidine, arginine and related compounds	Contribute to nutritional value and cellular metabolism; arginine is the physiological substrate for NOS-dependent NO synthesis	Cultivar, plant tissue and processing	[56,90,92,103]
Triterpenoid saponins	Betavulgarosides, oleanolic acid-derived saponins and related triterpenoids	Potential antioxidant, anti-inflammatory, hepatoprotective and metabolic activities	Plant tissue, particularly differences between roots and leaves, cultivar and environmental conditions	[104,105]
Carotenoids	β-Carotene, lutein and related xanthophylls	Antioxidant activity; β-carotene provides provitamin A activity, whereas lutein and related xanthophylls contribute to ocular antioxidant protection	Plant tissue, cultivar, growing conditions and processing; concentrations are generally more prominent in leaves than in roots	[2,7,56,90]

**Table 2:** Reported effects of fermentation on the composition, stability, bioaccessibility, and functional properties of beetroot products.

Fermentation System/Product	Microorganisms or Fermentation Conditions	Components Investigated	Reported Changes After Fermentation	Potential Relevance	Evidence Type	Reference
Fermented beetroot juice	*Lactiplantibacillus plantarum*, *Levilactobacillus brevis*, or *Lactobacillus acidophilus*; controlled lactic fermentation	Betalains, phenolic compounds, antioxidant and enzyme-inhibitory activities	Fermentation altered the betalain profile and phenolic composition. Isobetanin, neobetanin and other betalain derivatives were detected, while changes in flavonoids and antioxidant activity were strain- and inoculum-dependent. Fermented juices also showed inhibition of protein glycation in vitro	Demonstrates that microbial strain and fermentation conditions can selectively reshape beetroot phytochemicals and their measured in vitro activities	In vitro/food model	[27]
Lactic-fermented beetroot beverages with different matrices	Lactic acid fermentation; low-fibre juice versus high-fibre puree-based beverage	Phenolic compounds, phenolic bioaccessibility, radical-scavenging activity	Fermentation increased the proportion of free phenolics, but its effect on bioaccessibility depended strongly on the food matrix. In vitro phenolic bioaccessibility decreased in the juice but increased in the puree-based beverage	Shows that release of free phenolics does not necessarily translate into greater gastrointestinal bioaccessibility and highlights the importance of matrix effects	In vitro gastrointestinal digestion model	[46]
Fermented beetroot juice	Controlled lactic fermentation	Phenolic acids and flavonoids	Fermentation increased free phenolic acids and decreased conjugated phenolic acids, indicating redistribution of phenolic forms rather than a uniform increase in total phenolics	Supports the concept that fermentation modifies the chemical form and potential accessibility of phenolics	Food analysis; human exposure study	[133]
Fermented beetroot juice	Lactic acid bacteria; different formulations/additives	Total phenolics, flavonoids, vitamin C, antioxidant activity	Fermented juices differed substantially in phenolic and flavonoid contents and in FRAP, ABTS and DPPH responses depending on formulation	Demonstrates that formulation and fermentation conditions influence the chemical antioxidant potential of fermented beetroot products	Food/fermentation study; in vitro assays	[30]
Red beetroot fermented with different microbial consortia	Different microbial consortia under spontaneous/controlled fermentation conditions	Volatile organic compounds and sensory characteristics	The microbial consortium influenced the volatile profile, aroma, taste and other sensory attributes of fermented beetroot	Indicates that microbial composition is important not only for biochemical transformation but also for sensory quality and product development	Food/fermentation and sensory study	[115]
Lactic acid fermentation of red beetroot	LAB; different NaCl concentrations	Betalains and physicochemical properties	Salt concentration affected fermentation behaviour, physicochemical properties and betalain retention. The results demonstrate substantial interactions between salinity and pigment stability	Supports the need for process standardisation when preservation of betalains is a target	Food/fermentation study	[121]
Lactofermented beetroot juice	*Lactobacillus brevis* 0944 + *Lactobacillus paracasei* 0920	Gut microbiota, microbial metabolic activity, β-glucuronidase, antioxidant status	Dietary administration of lactofermented beetroot juice modulated intestinal microbiota and microbial activity in rats. β-Glucuronidase activity decreased, and antioxidant capacity of the aqueous serum fraction increased under the experimental conditions	Provides in vivo evidence that fermented beetroot may influence gut microbial metabolism and systemic redox status, although translation to humans remains uncertain	Animal study	[153]
*Lactobacillus*-fermented beetroot juice	*Lactobacillus casei* 0920 and *Lactobacillus brevis* 0944	Cecal microbiota and microbial metabolic activity	Fermented beetroot juice positively modulated the cecal microbial ecosystem and its metabolic activity in rats	Provides early evidence for microbiota-mediated effects of fermented beetroot	Animal study	[154]
Fermented beetroot product (PN39)	Defined fermented beetroot preparation	Gut microbiota, microbial carbohydrate metabolism, SCFAs, glucose tolerance	PN39 promoted changes in selected microbial taxa and increased short-chain fatty acid production. In mice receiving a high-fat diet, concomitant PN39 consumption prevented impairment of glucose tolerance and attenuated dysbiosis. Effects were not reproduced when PN39 was administered after prediabetes had already developed	Provides recent evidence linking fermented beetroot with gut microbial metabolism and metabolic outcomes, while demonstrating that effects depend on disease stage and experimental context	Animal study + ex vivo human microbiota model	[155]
Fermented beetroot	Spontaneous lactic fermentation	Phenolic acids, flavonoids, betalains; anti-glycation activity	Fermentation altered the phenolic and flavonoid composition of beetroot and changed its in vitro inhibition of AGE formation. The study identified differences in the relationship between phytochemical composition and anti-AGE activity before and after fermentation	Suggests that fermentation may modify functional activity through qualitative changes in the phytochemical profile rather than simply increasing total phenolic content	In vitro study	[156]
Fermented beetroot-derived LAB	*Lactococcus lactis* and *Weissella cibaria* isolated from pickled beetroot	B-group vitamins, lactic acid, antimicrobial properties and volatile compounds	*L. lactis* demonstrated the ability to produce riboflavin and niacin under selected culture conditions; both strains produced lactic acid and exhibited properties relevant to starter-culture development. Volatile compounds included alcohols, ketones, acids and aldehydes.	Indicates that beetroot fermentation can select microorganisms with metabolic and technological properties relevant to functional-food development	Microbiological/in vitro study	[120]
Fermented beetroot juice	Different LAB species and inoculum concentrations	Betalains, polyphenols, antioxidant capacity	Fermentation effects differed among *L. acidophilus*, *L. brevis* and *L. plantarum*. Changes included formation or enrichment of selected betalain derivatives, modification of polyphenol concentrations and strain-dependent changes in DPPH and ABTS activity	Supports a strain-specific model of fermentation in which microbial identity is an important determinant of the final phytochemical and functional profile	In vitro/food model	[27]

Abbreviations: AGE—advanced glycation end-products; ABTS—2,2′-azinobis-(3-ethylbenzothiazoline-6-sulfonic acid); DPPH—2,2-diphenyl-1-picrylhydrazyl; FRAP—ferric reducing antioxidant power; LAB—lactic acid bacteria; NO—nitric oxide; SCFAs—short-chain fatty acids; TPC—total phenolic content; TFC—total flavonoid content; CFU—colony-forming units.

**Table 3:** Evidence for biological and health effects of beetroot-derived products according to study design and fermentation status.

Study Design/Population	Product/Intervention	Main Bioactive Constituents	Main Outcome	Fermentation Status	Evidence Level	Main Limitation	Reference
Healthy men (*n* = 16)	250 mL beetroot juice vs. 300 g whole beetroot vs. placebo	Nitrate, betanin, betalains, polyphenols	Plasma NOx increased for up to 8 h after juice and 5 h after whole beetroot; betanin was not detected in plasma	Non-fermented	Human randomised crossover intervention	Small sample; acute exposure; no fermented comparator	[80]
Healthy volunteers	Dietary nitrate/nitrate-rich beetroot juice	Nitrate, nitrite, NO-related species	Increased circulating nitrate/nitrite and changes in vascular responses following nitrate intake	Non-fermented/isolated nitrate	Human intervention	Effects cannot be attributed specifically to fermented beetroot	[238]
Healthy adults (*n* = 40)	450 mL nitrate-rich beetroot juice vs. placebo	Nitrate	Altered prefrontal cerebral hemodynamics and improved performance on a serial subtraction task	Non-fermented	Randomised controlled human study	Acute intervention; modest sample; cognitive effects were task-specific	[239]
Older adults (~75 years)	High-nitrate diet including beetroot juice vs. low-nitrate diet	Dietary nitrate	Increased cerebral perfusion, particularly in frontal brain regions	Non-fermented	Human intervention	Acute intervention; small study; cerebral perfusion rather than long-term cognition	[240]
Older adults with type 2 diabetes (*n* = 27)	250 mL beetroot juice containing ~7.5 mmol nitrate/day for 2 weeks vs. nitrate-depleted placebo	Nitrate	Improved simple reaction time; no consistent improvement across other cognitive domains	Non-fermented	Randomised crossover human study	Small sample; selective cognitive outcome; short intervention	[241]
Healthy young adults/exercise study	Beetroot juice before exercise	Nitrate	Reduced cerebral deoxygenation during exercise, without clear improvement in reaction time or information processing	Non-fermented	Human intervention	Acute intervention; no fermented product; cognition not improved	[253]
Healthy adults/nitrate interventions	Beetroot juice and other dietary nitrate formulations	Nitrate, nitrite	Vascular and blood-pressure responses varied according to nitrate dose, formulation and timing	Non-fermented	Human intervention	Product heterogeneity limits direct comparison	[141]
Adults, RCTs	Dietary nitrate supplementation	Nitrate	Modest reductions in SBP and DBP; effects depended on intervention duration and measurement setting	Predominantly non-fermented	Systematic review/meta-analysis	Includes isolated nitrate and different nitrate-rich foods, not specifically fermented beetroot	[254]
Adults, 75 RCTs; *n* = 1823	Dietary nitrate supplementation	Nitrate	Dose–response increase in plasma nitrate and reductions in systolic BP; evidence also supported vascular effects	Predominantly non-fermented	GRADE-assessed systematic review/meta-analysis	Not specific to beetroot fermentation	[141]
Adults, 113 studies/2013 participants	Dietary nitrate supplementation	Nitrate	Reduced resting SBP and DBP, reduced PWV and augmentation index, and increased FMD	Predominantly non-fermented	Umbrella review and updated meta-analysis	Strong product and population heterogeneity; fermented beetroot not separately evaluated	[255]
HepG2 cells	Purified betanin and betaine	Betanin, betaine	Concentration-dependent inhibition of HepG2 cell proliferation; betanin showed greater activity than betaine	Non-fermented/isolated compounds	In vitro	Concentrations may exceed physiologically achievable systemic exposure	[256]
Rats with N-nitroso-N-methylurea-induced oxidative/biochemical disturbances	Lactofermented beetroot juice	Betalains, phenolics and fermentation-derived metabolites	Modulation of intestinal microbiota and metabolic activity; reduced β-glucuronidase activity; increased antioxidant capacity of the aqueous serum fraction in MNU-treated rats	Fermented	Animal intervention	Small animal study; effects differed among tissues; no direct human evidence	[153]
Beetroot beverages; in vitro digestion model	LAB-fermented beetroot juice and puree	Phenolic compounds, organic acids, betalains	Fermentation increased free phenolic content; antioxidant activity increased in juice, whereas phenolic bioaccessibility decreased in fermented juice but increased in fermented puree	Fermented	Food/in vitro study	Simulated digestion does not establish human bioavailability or clinical efficacy	[46]
Beetroot juice fermented with LAB	Fermented red beetroot juice	Betalains, polyphenols	Fermentation modified betalain and polyphenol profiles and increased/modified antioxidant and selected in vitro bioactivities; anti-AGE activity was detected in selected fermented preparations	Fermented	Food/in vitro study	Outcomes were assay-dependent; no human clinical endpoints	[27]
Beetroot juice fermented with different LAB strains	*L. acidophilus*, *L. brevis* or *L. plantarum* fermentation	Betalains, polyphenols	Fermentation produced strain-dependent changes in betalains and phenolics; selected products showed anti-AChE and anti-AGE activity in vitro	Fermented	Food/in vitro study	Biological activity depended strongly on strain and fermentation conditions	[27]
Wistar rats; 8-week feeding study	Lactofermented beetroot juice produced with *L. brevis* 0944 and *L. paracasei* 0920	Beetroot phytochemicals and fermentation products	Positive modulation of gut-associated microbiota and increased serum antioxidant capacity under chemically induced oxidative stress	Fermented	Animal intervention	Animal model; limited information on translation to human health	[229]
Healthy adults (*n* = 24)	200 mL fermented red beetroot juice/60 kg body weight for 42 days	Beetroot-derived compounds; nitrate and fermentation-related metabolites	Significant changes in selected haematological parameters and PFAS concentrations; no significant changes in measured blood biochemical parameters	Fermented	Human intervention	Small non-randomised design; intervention was not designed to test cardiovascular efficacy or clinical benefit	[257]
Healthy adults; short-term fermented beetroot studies	Fermented beetroot juice	Polyphenols, betalains, organic acids	Fermentation can modify phenolic and betalain composition and antioxidant capacity	Fermented	Human-relevant food evidence, but not clinical efficacy evidence	Limited controlled human trials; composition is highly strain- and process-dependent	[27,30]
Adults with Long COVID; randomised controlled pilot trial	200 mL beetroot juice/day containing ~600 mg nitrate for 14 days	Nitrate, beetroot phytochemicals	Altered gut microbiota and inflammatory/metabolic profiles, but no clear improvement in functional clinical outcomes	Non-fermented	Human randomised pilot study	Short duration; small sample; product was not fermented	[187]
Older adults	Nitrate-rich beetroot juice vs. nitrate-depleted placebo	Nitrate	Changes in nitrate-sensitive oral microbiota, including increased nitrate-reducing taxa	Non-fermented	Human randomised crossover evidence	Oral microbiome effects cannot be extrapolated directly to fermented beetroot	[234]

Abbreviations: AChE—acetylcholinesterase; AGE—advanced glycation end products; BP—blood pressure; DBP—diastolic blood pressure; FMD—flow-mediated dilation; GRADE—Grading of Recommendations Assessment, Development and Evaluation; LAB—lactic acid bacteria; Long COVID—post-COVID-19 condition; MNU—N-nitroso-N-methylurea; NO—nitric oxide; NOx—nitrogen oxides (primarily nitrate and nitrite); PFAS—per- and polyfluoroalkyl substances; PWV—pulse wave velocity; RCT—randomised controlled trial; SBP—systolic blood pressure; T2D—type 2 diabetes.

## Data Availability

No new data were created or analysed in this study. The data presented in this study are available within the article.

## References

[1] Chhikara N., Kushwaha K., Sharma P., Gat Y., Panghal A. (2019). Bioactive compounds of beetroot and utilization in food processing industry: A critical review. Food Chem..

[2] Stoica F., Râpeanu G., Rațu R.N., Stănciuc N., Croitoru C., Țopa D., Jităreanu G. (2025). Red Beetroot and Its By-Products: A Comprehensive Review of Phytochemicals, Extraction Methods, Health Benefits, and Applications. Agriculture.

[3] Calva-Estrada S.J., Jiménez-Fernández M., Lugo-Cervantes E. (2022). Betalains and their applications in food: The current state of processing, stability and future opportunities in the industry. Food Chem. Mol. Sci..

[4] Sadowska-Bartosz I., Bartosz G. (2021). Biological Properties and Applications of Betalains. Molecules.

[5] Guldiken B., Toydemir G., Nur Memis K., Okur S., Boyacioglu D., Capanoglu E. (2016). Home-Processed Red Beetroot (*Beta vulgaris* L.) Products: Changes in Antioxidant Properties and Bioaccessibility. Int. J. Mol. Sci..

[6] Ersoyak N., Acoglu Celik B., Koc Alibasoglu E., Bekar E., Unal T.T., Yetan E., Kamiloglu S., Akpinar Bayizit A., Copur O.U., Aral Baskaya I. (2025). Production of Red Beetroot (*Beta vulgaris* L.) Pestil as an Alternative Healthy Snack: Effects of Traditional, Thermosonication, and Microwave Pretreatments on Physicochemical, Sensorial, Nutritional, and Chemometric Profiles. Foods.

[7] Chen L., Zhu Y., Hu Z., Wu S., Jin C. (2021). Beetroot as a functional food with huge health benefits: Antioxidant, antitumor, physical function, and chronic metabolomics activity. Food Sci. Nutr..

[8] Shahidi F., Pan Y. (2022). Influence of food matrix and food processing on the chemical interaction and bioaccessibility of dietary phytochemicals: A review. Crit. Rev. Food Sci. Nutr..

[9] Lundberg J.O., Carlström M., Larsen F.J., Weitzberg E. (2011). Roles of dietary inorganic nitrate in cardiovascular health and disease. Cardiovasc. Res..

[10] Hezel M.P., Weitzberg E. (2015). The oral microbiome and nitric oxide homoeostasis. Oral Dis..

[11] McMahon N.F., Leveritt M.D., Pavey T.G. (2017). The Effect of Dietary Nitrate Supplementation on Endurance Exercise Performance in Healthy Adults: A Systematic Review and Meta-Analysis. Sports Med..

[12] Qu X.M., Wu Z.F., Pang B.X., Jin L.Y., Qin L.Z., Wang S.L. (2016). From Nitrate to Nitric Oxide: The Role of Salivary Glands and Oral Bacteria. J. Dent. Res..

[13] Raubenheimer K., Hickey D., Leveritt M., Fassett R., Ortiz de Zevallos Munoz J., Allen J.D., Briskey D., Parker T.J., Kerr G., Peake J.M. (2017). Acute Effects of Nitrate-Rich Beetroot Juice on Blood Pressure, Hemostasis and Vascular Inflammation Markers in Healthy Older Adults: A Randomized, Placebo-Controlled Crossover Study. Nutrients.

[14] Bahadoran Z., Mirmiran P., Kabir A., Azizi F., Ghasemi A. (2017). The Nitrate-Independent Blood Pressure-Lowering Effect of Beetroot Juice: A Systematic Review and Meta-Analysis. Adv. Nutr..

[15] Benjamim C.J.R., Porto A.A., Valenti V.E., Sobrinho A.C.D.S., Garner D.M., Gualano B., Bueno Júnior C.R. (2022). Nitrate Derived From Beetroot Juice Lowers Blood Pressure in Patients With Arterial Hypertension: A Systematic Review and Meta-Analysis. Front. Nutr..

[16] McLellan A.G., Acton J.P., O’Donnell E., Rowland S.N., Shepherd A.I., Perissiou M., James L.J., Clifford T., Bailey S.J. (2026). The dose-response effects of nitrate-rich beetroot ingestion on cardiovascular and endothelial function: A randomised controlled trial. Food Funct..

[17] Jones A.M. (2014). Dietary nitrate supplementation and exercise performance. Sports Med..

[18] Hoon M.W., Johnson N.A., Chapman P.G., Burke L.M. (2013). The effect of nitrate supplementation on exercise performance in healthy individuals: A systematic review and meta-analysis. Int. J. Sport Nutr. Exerc. Metab..

[19] Poon E.T., Iu J.C., Sum W.M., Wong P.S., Lo K.K., Ali A., Burns S.F., Trexler E.T. (2025). Dietary Nitrate Supplementation and Exercise Performance: An Umbrella Review of 20 Published Systematic Reviews with Meta-analyses. Sports Med..

[20] Dalmau M.E., Llabrés P.J., Eim V.S., Rosselló C., Simal S. (2019). Influence of freezing on the bioaccessibility of beetroot (*Beta vulgaris*) bioactive compounds during in vitro gastric digestion. J. Sci. Food Agric..

[21] Nowak K.W., Miszczak I., Pszczółkowski B., Rejmer W., Zielińska M. (2025). Application of Ultrasound in Convective Drying of Fermented, Frozen-Thawed, and Osmotically Dehydrated Beetroot Slices. Pol. J. Food Nutr. Sci..

[22] Martinez R.M., Melo C.P.B., Pinto I.C., Mendes-Pierotti S., Vignoli J.A., Verri W.A., Casagrande R. (2024). Betalains: A Narrative Review on Pharmacological Mechanisms Supporting the Nutraceutical Potential Towards Health Benefits. Foods.

[23] Dissanayake I.H., Tabassum W., Alsherbiny M., Chang D., Li C.G., Bhuyan D.J. (2025). Lactic acid bacterial fermentation as a biotransformation strategy to enhance the bioavailability of phenolic antioxidants in fruits and vegetables: A comprehensive review. Food Res. Int..

[24] Marco M.L., Sanders M.E., Gänzle M., Arrieta M.C., Cotter P.D., De Vuyst L., Hill C., Holzapfel W., Lebeer S., Merenstein D. (2021). The International Scientific Association for Probiotics and Prebiotics (ISAPP) consensus statement on fermented foods. Nat. Rev. Gastroenterol. Hepatol..

[25] Sawicki T., Wiczkowski W. (2018). The effects of boiling and fermentation on betalain profiles and antioxidant capacities of red beetroot products. Food Chem..

[26] Choińska R., Piasecka-Jóźwiak K., Woźniak Ł., Świder O., Bartosiak E., Bujak M., Roszko M.Ł. (2022). Starter culture-related changes in free amino acids, biogenic amines profile, and antioxidant properties of fermented red beetroot grown in Poland. Sci. Rep..

[27] Sawicki T., Jabłońska M., Starowicz M., Kłębukowska L., Błaszczak W. (2024). The effects of fermentation by different species of lactic acid bacteria on betalains and polyphenol profile and in vitro bioactive potential of red beetroot juice. J. Food Drug Anal..

[28] Bescos R., Rollason M.L., Davies T.S., Casas-Agustench P. (2023). Content of nitrate and nitrite in commercial and self-made beetroot juices and the effect of storage temperature. Food Sci. Nutr..

[29] Janiszewska-Turak E., Walczak M., Rybak K., Pobiega K., Gniewosz M., Woźniak Ł., Witrowa-Rajchert D. (2022). Influence of Fermentation Beetroot Juice Process on the Physico-Chemical Properties of Spray Dried Powder. Molecules.

[30] Jakubczyk K., Melkis K., Janda-Milczarek K., Skonieczna-Żydecka K. (2024). Phenolic Compounds and Antioxidant Properties of Fermented Beetroot Juices Enriched with Different Additives. Foods.

[31] Czyżowska A., Siemianowska K., Śniadowska M., Nowak A. (2020). Bioactive compounds and microbial quality of red beetroots and stored fermented red beetroot juice. Pol. J. Food Nutr. Sci..

[32] Küçükgöz K., Venema K., Chamorro F., Cassani L., Donn P., Prieto M.A., Trząskowska M. (2025). Unlocking the potential of fermented beetroot ketchup: Enhancing polyphenol recovery and gut microbiota interactions. Food Chem..

[33] Fejes R., Lutnik M., Weisshaar S., Pilat N., Wagner K.H., Stüger H.P., Peake J.M., Woodman R.J., Croft K.D., Bondonno C.P. (2024). Increased nitrate intake from beetroot juice over 4 weeks affects nitrate metabolism, but not vascular function or blood pressure in older adults with hypertension. Food Funct..

[34] Mrakic-Sposta S., Vezzoli A., Parenza M., Magno M., D’Angelo G., Nannipieri F., Battaglia S., Solfanelli L., Tacconi E., Dellanoce C. (2026). Effect of Beetroot Nitrate Supplementation on Nitric Oxide Pathways and Oxy-Inflammatory Biomarkers in Amateur Triathletes: A Randomized Cross-Over Pilot Study. Nutrients.

[35] Haswell C., Rutherfurd-Markwick K., Hurst R., Wong M., Mazahery H., Ali A., Page R. (2026). Effects of Nitrate- and Betalain-Rich Beetroot Juice Ingestion on Vascular Function and Metabolic Syndrome Risk Factors in Healthy Adults: A Pilot Study. Metabolites.

[36] Grönroos R., Eggertsen R., Bernhardsson S., Praetorius Björk M. (2024). Effects of beetroot juice on blood pressure in hypertension according to European Society of Hypertension Guidelines: A systematic review and meta-analysis. Nutr. Metab. Cardiovasc. Dis..

[37] Farid M.S., Łopusiewicz Ł. (2026). Fermentation-enhanced functional properties of beetroot: From bioactive compounds to novel food products. Trends Food Sci. Technol..

[38] Kapil V., Khambata R.S., Robertson A., Caulfield M.J., Ahluwalia A. (2015). Dietary nitrate provides sustained blood pressure lowering in hypertensive patients: A randomized, phase 2, double-blind, placebo-controlled study. Hypertension.

[39] Miller G.D., Collins S., Ives J., Williams A., Basu S., Kim-Shapiro D.B., Berry M.J. (2023). Efficacy and Variability in Plasma Nitrite Levels during Long-Term Supplementation with Nitrate Containing Beetroot Juice. J. Diet. Suppl..

[40] Koch C.D., Gladwin M.T., Freeman B.A., Lundberg J.O., Weitzberg E., Morris A. (2017). Enterosalivary nitrate metabolism and the microbiome: Intersection of microbial metabolism, nitric oxide and diet in cardiac and pulmonary vascular health. Free Radic. Biol. Med..

[41] Blekkenhorst L.C., Bondonno N.P., Liu A.H., Ward N.C., Prince R.L., Lewis J.R., Devine A., Croft K.D., Hodgson J.M., Bondonno C.P. (2018). Nitrate, the oral microbiome, and cardiovascular health: A systematic literature review of human and animal studies. Am. J. Clin. Nutr..

[42] Govoni M., Jansson E.A., Weitzberg E., Lundberg J.O. (2008). The increase in plasma nitrite after a dietary nitrate load is markedly attenuated by an antibacterial mouthwash. Nitric Oxide.

[43] McDonagh S.T., Wylie L.J., Winyard P.G., Vanhatalo A., Jones A.M. (2015). The effects of chronic nitrate supplementation and the use of strong and weak antibacterial agents on plasma nitrite concentration and exercise blood pressure. Int. J. Sports Med..

[44] Woessner M., Smoliga J.M., Tarzia B., Stabler T., Van Bruggen M., Allen J.D. (2016). A stepwise reduction in plasma and salivary nitrite with increasing strengths of mouthwash following a dietary nitrate load. Nitric Oxide.

[45] Küçükgöz K., Venema K., Trząskowska M. (2024). Gut microbiota modulatory capacity of fermented ketchup in a validated in vitro model of the colon. Food Res. Int..

[46] Mana de Wolf R.X., Hider R.N., Breitmeyer J., Serventi L. (2025). Effect of lactic fermentation and matrix on phenolic content, bioaccessibility, and scavenging activity of beetroot beverages. Eur. Food Res. Technol..

[47] Wruss J., Waldenberger G., Huemer S., Uygun P., Lanzerstorfer P., Müller U., Höglinger O., Weghuber J. (2015). Compositional characteristics of commercial beetroot products and beetroot juice prepared from seven beetroot varieties grown in Upper Austria. J. Food Compos. Anal..

[48] Clifford T., Howatson G., West D.J., Stevenson E.J. (2015). The potential benefits of red beetroot supplementation in health and disease. Nutrients.

[49] Fu Y., Shi J., Xie S.Y., Zhang T.Y., Soladoye O.P., Aluko R.E. (2020). Red Beetroot Betalains: Perspectives on Extraction, Processing, and Potential Health Benefits. J. Agric. Food Chem..

[50] Sawicki T., Bączek N., Wiczkowski W. (2016). Betalain profile, content and antioxidant capacity of red beetroot dependent on the genotype and root part. J. Funct. Foods.

[51] Ribas-Agustí A., Martín-Belloso O., Soliva-Fortuny R., Elez-Martínez P. (2018). Food processing strategies to enhance phenolic compounds bioaccessibility and bioavailability in plant-based foods. Crit. Rev. Food Sci. Nutr..

[52] Vareltzis P., Kyroglou S., Pasidi E., Oikonomou G., Gkogkou T., Govari M., Kalogiannis K., Gortzi O. (2026). Food Matrix Effects on Plant-Derived Bioactive Compounds and Micronutrients: Implications for Functional Food Development. Int. J. Mol. Sci..

[53] Wang T., Liu L., Rakhmanova A., Wang X., Shan Y., Yi Y., Liu B., Zhou Y., Lü X. (2020). Stability of Bioactive Compounds and In Vitro Gastrointestinal Digestion of Red Beetroot Jam: Effect of Processing and Storage. Food Biosci..

[54] Coles L.T., Clifton P.M. (2012). Effect of Beetroot Juice on Lowering Blood Pressure in Free-Living, Disease-Free Adults: A Randomized, Placebo-Controlled Trial. Nutr. J..

[55] Domínguez R., Cuenca E., Maté-Muñoz J.L., García-Fernández P., Serra-Paya N., Estevan M.C., Herreros P.V., Garnacho-Castaño M.V. (2017). Effects of Beetroot Juice Supplementation on Cardiorespiratory Endurance in Athletes: A Systematic Review. Nutrients.

[56] Baião D.D.S., Silva D.V.T.D., Paschoalin V.M.F. (2020). Beetroot, a Remarkable Vegetable: Its Nitrate and Phytochemical Contents Can Be Adjusted in Novel Formulations to Benefit Health and Support Cardiovascular Disease Therapies. Antioxidants.

[57] Lundberg J.O., Weitzberg E., Gladwin M.T. (2008). The Nitrate-Nitrite-Nitric Oxide Pathway in Physiology and Therapeutics. Nat. Rev. Drug Discov..

[58] Förstermann U., Sessa W.C. (2012). Nitric Oxide Synthases: Regulation and Function. Eur. Heart J..

[59] Lundberg J.O., Carlström M., Weitzberg E. (2018). Metabolic Effects of Dietary Nitrate in Health and Disease. Cell Metab..

[60] Hyde E.R., Andrade F., Vaksman Z., Parthasarathy K., Jiang H., Parthasarathy D.K., Torregrossa A.C., Tribble G., Kaplan H.B., Petrosino J.F. (2014). Metagenomic Analysis of Nitrate-Reducing Bacteria in the Oral Cavity: Implications for Nitric Oxide Homeostasis. PLoS ONE.

[61] Gladwin M.T., Raat N.J., Shiva S., Dezfulian C., Hogg N., Kim-Shapiro D.B., Patel R.P. (2006). Nitrite as a Vascular Endocrine Nitric Oxide Reservoir That Contributes to Hypoxic Signaling, Cytoprotection, and Vasodilation. Am. J. Physiol. Heart Circ. Physiol..

[62] Lundberg J.O., Gladwin M.T., Ahluwalia A., Benjamin N., Bryan N.S., Butler A., Cabrales P., Fago A., Feelisch M., Ford P.C. (2009). Nitrate and Nitrite in Biology, Nutrition and Therapeutics. Nat. Chem. Biol..

[63] Moncada S., Palmer R.M., Higgs E.A. (1991). Nitric Oxide: Physiology, Pathophysiology, and Pharmacology. Pharmacol. Rev..

[64] Francis S.H., Busch J.L., Corbin J.D., Sibley D. (2010). cGMP-Dependent Protein Kinases and cGMP Phosphodiesterases in Nitric Oxide and cGMP Action. Pharmacol. Rev..

[65] Siervo M., Lara J., Ogbonmwan I., Mathers J.C. (2013). Inorganic Nitrate and Beetroot Juice Supplementation Reduces Blood Pressure in Adults: A Systematic Review and Meta-Analysis. J. Nutr..

[66] Förstermann U. (2010). Nitric Oxide and Oxidative Stress in Vascular Disease. Pflug. Arch..

[67] Pacher P., Beckman J.S., Liaudet L. (2007). Nitric Oxide and Peroxynitrite in Health and Disease. Physiol. Rev..

[68] Milton-Laskibar I., Martínez J.A., Portillo M.P. (2021). Current Knowledge on Beetroot Bioactive Compounds: Role of Nitrate and Betalains in Health and Disease. Foods.

[69] Esatbeyoglu T., Wagner A.E., Schini-Kerth V.B., Rimbach G. (2015). Betanin—A Food Colorant with Biological Activity. Mol. Nutr. Food Res..

[70] Allegra M., Furtmüller P.G., Jantschko W., Zederbauer M., Tesoriere L., Livrea M.A., Obinger C. (2005). Mechanism of Interaction of Betanin and Indicaxanthin with Human Myeloperoxidase and Hypochlorous Acid. Biochem. Biophys. Res. Commun..

[71] Reddy M.K., Alexander-Lindo R.L., Nair M.G. (2005). Relative Inhibition of Lipid Peroxidation, Cyclooxygenase Enzymes, and Human Tumor Cell Proliferation by Natural Food Colors. J. Agric. Food Chem..

[72] Szabó C. (2003). Multiple Pathways of Peroxynitrite Cytotoxicity. Toxicol. Lett..

[73] Carreón-Hidalgo J.P., Franco-Vásquez D.C., Gómez-Linton D.R., Pérez-Flores L.J. (2022). Betalain Plant Sources, Biosynthesis, Extraction, Stability Enhancement Methods, Bioactivity, and Applications. Food Res. Int..

[74] Khan M.I. (2016). Plant Betalains: Safety, Antioxidant Activity, Clinical Efficacy, and Bioavailability. Compr. Rev. Food Sci. Food Saf..

[75] Güneşer O. (2016). Pigment and Color Stability of Beetroot Betalains in Cow Milk during Thermal Treatment. Food Chem..

[76] Gandía-Herrero F., Escribano J., García-Carmona F. (2016). Biological Activities of Plant Pigments Betalains. Crit. Rev. Food Sci. Nutr..

[77] Vieira Teixeira da Silva D., Dos Santos Baião D., de Oliveira Silva F., Alves G., Perrone D., Mere Del Aguila E., M Flosi Paschoalin V. (2019). Betanin, a Natural Food Additive: Stability, Bioavailability, Antioxidant and Preservative Ability Assessments. Molecules.

[78] Igual M., Fernandes Â., Dias M.I., Pinela J., García-Segovia P., Martínez-Monzó J., Barros L. (2023). The In Vitro Simulated Gastrointestinal Digestion Affects the Bioaccessibility and Bioactivity of *Beta vulgaris* Constituents. Foods.

[79] Frank T., Stintzing F.C., Carle R., Bitsch I., Quaas D., Strass G., Bitsch R., Netzel M. (2005). Urinary Pharmacokinetics of Betalains Following Consumption of Red Beet Juice in Healthy Humans. Pharmacol. Res..

[80] Wootton-Beard P.C., Brandt K., Fell D., Warner S., Ryan L. (2014). Effects of a Beetroot Juice with High Neobetanin Content on the Early-Phase Insulin Response in Healthy Volunteers. J. Nutr. Sci..

[81] Massoud A., Essawy A.E., Alfredan M.A., Abdel-Moneim A.M., Gomaa R.A., Abdel Salam S. (2026). Betanin from *Beta vulgaris* Attenuates Complete Freund’s Adjuvant-Induced Inflammatory Pain: Integrated Preclinical and In Silico Insights. Biomedicines.

[82] Vulić J.J., Cebović T.N., Canadanović V.M., Cetković G.S., Djilas S.M., Canadanović-Brunet J.M., Velićanski A.S., Cvetković D.D., Tumbas V.T. (2013). Antiradical, Antimicrobial and Cytotoxic Activities of Commercial Beetroot Pomace. Food Funct..

[83] Rahimi P., Mesbah-Namin S.A., Ostadrahimi A., Abedimanesh S., Separham A., Asghary Jafarabadi M. (2019). Effects of Betalains on Atherogenic Risk Factors in Patients with Atherosclerotic Cardiovascular Disease. Food Funct..

[84] Mansouri-Baseri A., Moohebati M., Bahrami L.S., Tabesh H., Rezaie M., Nematy M., Davoudi F., Rezvani R. (2025). Effect of Beetroot Extract Supplementation on Serum Fatty Acid Profiles and Oxidative Stress Markers in Chronic Coronary Artery Disease Patients: A Secondary Analysis of a Randomized Controlled Trial. Biomed. Res. Int..

[85] Purushothaman J.R., Rizwanullah M. (2024). Ferulic Acid: A Comprehensive Review. Cureus.

[86] Almatroodi S.A., Rahmani A.H. (2026). Caffeic Acid and Human Health: Evidence-Based Roles in Disease Prevention and Treatment. Int. J. Mol. Sci..

[87] Nguyen V., Taine E.G., Meng D., Cui T., Tan W. (2024). Chlorogenic Acid: A Systematic Review on the Biological Functions, Mechanistic Actions, and Therapeutic Potentials. Nutrients.

[88] Hadidi M., Liñán-Atero R., Tarahi M., Christodoulou M.C., Aghababaei F. (2024). The Potential Health Benefits of Gallic Acid: Therapeutic and Food Applications. Antioxidants.

[89] Pavlíková N. (2023). Caffeic Acid and Diseases—Mechanisms of Action. Int. J. Mol. Sci..

[90] Tan M.L., Hamid S.B.S. (2021). Beetroot as a Potential Functional Food for Cancer Chemoprevention, a Narrative Review. J. Cancer Prev..

[91] Kumar S., Pandey A.K. (2013). Chemistry and Biological Activities of Flavonoids: An Overview. Sci. World J..

[92] Baião D.S., Silva F.O., d’El-Rei J., Neves M.F., Perrone D., Del Aguila E.M., Paschoalin V.M.F. (2018). A New Functional Beetroot Formulation Enhances Adherence to Nitrate Supplementation and Health Outcomes in Clinical Practice. SDRP J. Food Sci. Technol..

[93] Panchal P., Miller A.J., Giri J. (2021). Organic Acids: Versatile Stress-Response Roles in Plants. J. Exp. Bot..

[94] Tang X., Liu J., Dong W., Li P., Li L., Lin C., Zheng Y., Hou J., Li D. (2013). The Cardioprotective Effects of Citric Acid and L-Malic Acid on Myocardial Ischemia/Reperfusion Injury. Evid.-Based Complement. Altern. Med..

[95] Mahdavian K. (2025). Synergistic Modulation of Antioxidant Enzymes by Citric Acid and EDTA to Enhance Lead Tolerance and Phytoextraction Efficiency in Harmel. Sci. Rep..

[96] Gęgotek A., Skrzydlewska E. (2022). Antioxidative and Anti-Inflammatory Activity of Ascorbic Acid. Antioxidants.

[97] Chaudhary P., Janmeda P., Docea A.O., Yeskaliyeva B., Abdull Razis A.F., Modu B., Calina D., Sharifi-Rad J. (2023). Oxidative Stress, Free Radicals and Antioxidants: Potential Crosstalk in the Pathophysiology of Human Diseases. Front. Chem..

[98] Zhang W., Lang R. (2023). Succinate Metabolism: A Promising Therapeutic Target for Inflammation, Ischemia/Reperfusion Injury and Cancer. Front. Cell Dev. Biol..

[99] Arumugam M.K., Paal M.C., Donohue T.M., Ganesan M., Osna N.A., Kharbanda K.K. (2021). Beneficial Effects of Betaine: A Comprehensive Review. Biology.

[100] Tang X., Wu B., Luo Y., Peng L., Chen Y., Zhu J., Peng C., Li S., Liu J. (2017). Effect of Potassium Supplementation on Vascular Function: A Meta-Analysis of Randomized Controlled Trials. Int. J. Cardiol..

[101] Liguori S., Moretti A., Paoletta M., Gimigliano F., Iolascon G. (2024). Role of Magnesium in Skeletal Muscle Health and Neuromuscular Diseases: A Scoping Review. Int. J. Mol. Sci..

[102] Jomova K., Alomar S.Y., Valko R., Nepovimova E., Kuca K., Valko M. (2025). The Role of Redox-Active Iron, Copper, Manganese, and Redox-Inactive Zinc in Toxicity, Oxidative Stress, and Human Diseases. EXCLI J..

[103] Kurhaluk N., Tkaczenko H. (2025). L-Arginine and Nitric Oxide in Vascular Regulation—Experimental Findings in the Context of Blood Donation. Nutrients.

[104] Mroczek A., Kapusta I., Stochmal A., Janiszowska W. (2019). MS/MS and UPLC-MS Profiling of Triterpenoid Saponins from Leaves and Roots of Four Red Beet (*Beta vulgaris* L.) Cultivars. Phytochem. Lett..

[105] Günther A., Bednarczyk-Cwynar B. (2025). Oleanolic Acid: A Promising Antioxidant—Sources, Mechanisms of Action, Therapeutic Potential, and Enhancement of Bioactivity. Antioxidants.

[106] Spórna-Kucab A., Tekieli A., Grzegorczyk A., Świątek Ł., Boguszewska A., Skalicka-Woźniak K. (2023). Betaxanthin Profiling in Relation to the Biological Activities of Red and Yellow *Beta vulgaris* L. Extracts. Metabolites.

[107] Skalicky M., Kubes J., Shokoofeh H., Tahjib-Ul-Arif M., Vachova P., Hejnak V. (2020). Betacyanins and Betaxanthins in Cultivated Varieties of *Beta vulgaris* L. Compared to Weed Beets. Molecules.

[108] Santamaria P. (2006). Nitrate in vegetables: Toxicity, content, intake and EC regulation. J. Sci. Food Agric..

[109] Sobhy E.S., Abdo E., Shaltout O., Abdalla A., Zeitoun A. (2020). Nutritional evaluation of beetroots (*Beta vulgaris* L.) and its potential application in a functional beverage. Plants.

[110] Preczenhak A.P., Orsi B., Lima G.P.P., Tezotto-Uliana J.V., Minatel I.O., Kluge R.A. (2019). Cysteine enhances the content of betalains and polyphenols in fresh-cut red beet. Food Chem..

[111] Herbach K.M., Stintzing F.C., Carle R. (2006). Betalain stability and degradation—Structural and chromatic aspects. J. Food Sci..

[112] Ramírez-Melo L.M., Cruz-Cansino N.d.S., Delgado-Olivares L., Ramírez-Moreno E., Zafra-Rojas Q.Y., Hernández-Traspeña J.L., Suárez-Jacobo Á. (2022). Optimization of antioxidant activity properties of a thermosonicated beetroot (*Beta vulgaris* L.) juice and further in vitro bioaccessibility comparison with thermal treatments. LWT.

[113] Trych U., Buniowska-Olejnik M., Marszałek K. (2022). Bioaccessibility of betalains in beetroot (*Beta vulgaris* L.) juice under different high-pressure techniques. Molecules.

[114] Di Cagno R., Coda R., De Angelis M., Gobbetti M. (2013). Exploitation of vegetables and fruits through lactic acid fermentation. Food Microbiol..

[115] Casciano F., Mayr H., Nissen L., Putti A., Zoli F., Gianotti A., Conterno L. (2022). Red beetroot fermentation with different microbial consortia to develop foods with improved aromatic features. Foods.

[116] Kazimierczak R., Hallmann E., Lipowski J., Drela N., Kowalik A., Püssa T., Matt D., Luik A., Gozdowski D., Rembiałkowska E. (2014). Beetroot (*Beta vulgaris* L.) and naturally fermented beetroot juices from organic and conventional production: Metabolomics, antioxidant levels and anticancer activity. J. Sci. Food Agric..

[117] Staninska-Pięta J., Czarny J., Wolko Ł., Cyplik P., Drożdżyńska A., Przybylak M., Ratajczak K., Piotrowska-Cyplik A. (2023). Temperature, salinity and garlic additive shape the microbial community during traditional beetroot fermentation process. Foods.

[118] Miszczak I., Tańska M., Rejmer W., Konopka I., Zielińska M. (2024). Spontaneous fermentation of beetroot: Effect of fermentation time and temperature and slice thickness on leaven quality. Pol. J. Food Nutr. Sci..

[119] Kumari V.B.C., Huligere S.S., Ramu R., Naik Bajpe S., Sreenivasa M.Y., Silina E., Stupin V., Achar R.R. (2022). Evaluation of probiotic and antidiabetic attributes of *Lactobacillus* strains isolated from fermented beetroot. Front. Microbiol..

[120] Maślak E., Złoch M., Arendowski A., Sugajski M., Janczura I., Rudnicka J., Walczak-Skierska J., Buszewska-Forajta M., Rafińska K., Pomastowski P. (2022). Isolation and identification of *Lactococcus lactis* and *Weissella cibaria* strains from fermented beetroot and investigation of their properties as potential starter cultures and probiotics. Foods.

[121] Janiszewska-Turak E., Wierzbicka A., Rybak K., Pobiega K., Synowiec A., Woźniak Ł., Trych U., Krzykowski A., Gramza-Michałowska A. (2024). Studying the influence of salt concentrations on betalain and selected physical and chemical properties in the lactic acid fermentation process of red beetroot. Molecules.

[122] Gangakhedkar P.S., Deshpande H.W., Törős G., El-Ramady H., Elsakhawy T., Abdalla N., Shaikh A., Kovács B., Mane R., Prokisch J. (2025). Fermentation of fruits and vegetables: Bridging traditional wisdom and modern science for food preservation and nutritional value improvements. Foods.

[123] Jung S., Hwang I.M., Lee J.H. (2024). Temperature impact on microbial and metabolic profiles in kimchi fermentation. Heliyon.

[124] Salinas-Ruiz J.P., Guevara García J., Rios Tovar D., Cruz Cabrera R.P., Torrico D.D. (2026). Effects of fermentation time and temperature on the physicochemical quality of kombucha. Foods.

[125] Lund P.A., De Biase D., Liran O., Scheler O., Mira N.P., Cetecioglu Z., Fernández E.N., Bover-Cid S., Hall R., Sauer M. (2020). Understanding how microorganisms respond to acid pH is central to their control and successful exploitation. Front. Microbiol..

[126] Heród-Leszczyńska T., Miedzobrodzka A. (1992). Wpływ procesu fermentacji na zmianę poziomu azotanów i azotynów w wybranych warzywach [Effect of the fermentation process on levels of nitrates and nitrites in selected vegetables]. Rocz. Państw. Zakł. Hig..

[127] Ding Z., Johanningsmeier S.D., Price R., Reynolds R., Truong V.-D., Payton S.C., Breidt F. (2018). Evaluation of nitrate and nitrite contents in pickled fruit and vegetable products. Food Control.

[128] Andersson R.E. (1985). Nitrate reduction during fermentation by Gram-negative bacterial activity in carrots. Int. J. Food Microbiol..

[129] Sohn H., Chang Y.H., Yune J.H., Jeong C.H., Shin D.M., Kwon H.C., Kim D.H., Hong S.W., Hwang H., Jeong J.Y. (2020). Probiotic properties of *Lactiplantibacillus plantarum* LB5 isolated from kimchi based on nitrate reducing capability. Foods.

[130] González-Soltero R., Bailén M., de Lucas B., Ramírez-Goercke M.I., Pareja-Galeano H., Larrosa M. (2020). Role of oral and gut microbiota in dietary nitrate metabolism and its impact on sports performance. Nutrients.

[131] Yang Z., Du C., Chang Z., Yang Y., Hu L. (2025). Role of oral and gut microbiomes in enterosalivary nitrate metabolism and their effects on systemic disease. Front. Cell. Infect. Microbiol..

[132] Hu X., Gao F., Cheng M., Zhang M., Yang B., Wei W., Zhu L., Xiao X. (2025). Genotype and phenotype-based elucidation of the dominant microbial community and metabolic characteristics responsible for nitrite hazards in fermented vegetable. LWT.

[133] Płatosz N., Sawicki T., Wiczkowski W. (2020). Profile of phenolic acids and flavonoids of red beet and its fermentation products. Pol. J. Food Nutr. Sci..

[134] Lidder S., Webb A.J. (2013). Vascular effects of dietary nitrate (as found in green leafy vegetables and beetroot) via the nitrate-nitrite-nitric oxide pathway. Br. J. Clin. Pharmacol..

[135] Ozaki M.M., Munekata P.E.S., Jacinto-Valderrama R.A., Efraim P., Pateiro M., Lorenzo J.M., Pollonio M.A.R. (2021). Beetroot and radish powders as natural nitrite source for fermented dry sausages. Meat Sci..

[136] Zhou Q., Zang S., Zhao Z., Li X. (2018). Dynamic changes of bacterial communities and nitrite character during northeastern Chinese sauerkraut fermentation. Food Sci. Biotechnol..

[137] Pennisi L., Verrocchi E., Paludi D., Vergara A. (2020). Effects of vegetable powders as nitrite alternative in Italian dry fermented sausage. Ital. J. Food Saf..

[138] Stanaway L., Rutherfurd-Markwick K., Page R., Wong M., Jirangrat W., Teh K.H., Ali A. (2019). Acute Supplementation with Nitrate-Rich Beetroot Juice Causes a Greater Increase in Plasma Nitrite and Reduction in Blood Pressure of Older Compared to Younger Adults. Nutrients.

[139] van der Avoort C.M.T., Jonvik K.L., Nyakayiru J., van Loon L.J.C., Hopman M.T.E., Verdijk L.B. (2020). A nitrate-rich vegetable intervention elevates plasma nitrate and nitrite concentrations and reduces blood pressure in healthy young adults. J. Acad. Nutr. Diet..

[140] Siervo M., Shannon O., Kandhari N., Prabhakar M., Fostier W., Köchl C., Rogathi J., Temu G., Stephan B.C.M., Gray W.K. (2020). Nitrate-Rich Beetroot Juice Reduces Blood Pressure in Tanzanian Adults with Elevated Blood Pressure: A Double-Blind Randomized Controlled Feasibility Trial. J. Nutr..

[141] Norouzzadeh M., Hasan Rashedi M., Ghaemi S., Saber N., Mirdar Harijani A., Habibi H., Mostafavi S., Sarv F., Farhadnejad H., Teymoori F. (2025). Plasma Nitrate, Dietary Nitrate, Blood Pressure, and Vascular Health Biomarkers: A GRADE-Assessed Systematic Review and Dose-Response Meta-Analysis of Randomized Controlled Trials. Nutr. J..

[142] Martins N., Roriz C.L., Morales P., Barros L., Ferreira I.C.F.R. (2017). Coloring attributes of betalains: A key emphasis on stability and future applications. Food Funct..

[143] Shahidi F., Hossain A. (2023). Importance of Insoluble-Bound Phenolics to the Antioxidant Potential Is Dictated by Source Material. Antioxidants.

[144] Zhang P., Zhang J., Li L., Gu T., Chen S., Wang J., Gao M. (2025). The Release of Bound Phenolics to Enhance the Antioxidant Activity of Cornmeal by Liquid Fermentation with *Bacillus subtilis*. Foods.

[145] Rocchetti G., Perez Gregorio R., Lorenzo J.M., Barba F.J., García Oliveira P., Prieto M.A., Simal-Gandara J., Mosele J.I., Motilva M.-J. (2022). Functional implications of bound phenolic compounds and phenolics–food interaction: A review. Compr. Rev. Food Sci. Food Saf..

[146] Deseva I., Stoyanova M., Petkova N., Mihaylova D. (2020). Red beetroot juice phytochemicals bioaccessibility: An in vitro approach. Pol. J. Food Nutr. Sci..

[147] Nicolescu A., Babotă M., Barros L., Rocchetti G., Lucini L., Tanase C., Mocan A., Bunea C.I., Crișan G. (2023). Bioaccessibility and bioactive potential of different phytochemical classes from nutraceuticals and functional foods. Front. Nutr..

[148] Wang Y., Adekolurejo O.O., Wang B., McDermott K., Do T., Marshall L.J., Boesch C. (2024). Bioavailability and excretion profile of betacyanins—Variability and correlations between different excretion routes. Food Chem..

[149] Swallah M.S., Fu H., Sun H., Affoh R., Yu H. (2020). The impact of polyphenol on general nutrient metabolism in the monogastric gastrointestinal tract. J. Food Qual..

[150] Arfaoui L. (2021). Dietary Plant Polyphenols: Effects of Food Processing on Their Content and Bioavailability. Molecules.

[151] Tang Y., Liu W., Zhang J., Juan B., Zhu Y., Zhu L., Zhao Y., Daglia M., Xiao X., He Y. (2025). Advances in Intestinal-Targeted Release of Phenolic Compounds. Nutrients.

[152] Yang F., Chen C., Ni D., Yang Y., Tian J., Li Y., Chen S., Ye X., Wang L. (2023). Effects of Fermentation on Bioactivity and the Composition of Polyphenols Contained in Polyphenol-Rich Foods: A Review. Foods.

[153] Klewicka E., Zduńczyk Z., Juśkiewicz J., Klewicki R. (2015). Effects of lactofermented beetroot juice alone or with N-nitroso-N-methylurea on selected metabolic parameters, composition of the microbiota adhering to the gut epithelium and antioxidant status of rats. Nutrients.

[154] Klewicka E., Zduńczyk Z., Juśkiewicz J. (2009). Effect of *lactobacillus* fermented beetroot juice on composition and activity of cecal microflora of rats. Eur. Food Res. Technol..

[155] Daliri E.B., Megur A., Mingaila J., Vijaya A.K., Balnionytė T., Sakar D., Carnicero-Mayo Y., Behrends V., Costabile A., Burokas A. (2025). Fermented beetroot modulates gut microbial carbohydrate metabolism in prediabetes and prevents high-fat diet induced hyperglycemia in a prediabetic model. Curr. Res. Food Sci..

[156] Starowicz M., Płatosz N., Bączek N., Szawara-Nowak D., Šimková K., Wiczkowski W. (2024). Unraveling the in vitro anti-advanced glycation end-product (anti-AGE) potential of fermented red cabbage and beetroot: Insights into composition and activities. Foods.

[157] Duncan C., Dougall H., Johnston P., Green S., Brogan R., Leifert C., Smith L., Golden M., Benjamin N. (1995). Chemical Generation of Nitric Oxide in the Mouth from the Enterosalivary Circulation of Dietary Nitrate. Nat. Med..

[158] Lundberg J.O., Weitzberg E. (2009). NO Generation from Inorganic Nitrate and Nitrite: Role in Physiology, Nutrition and Therapeutics. Arch. Pharm. Res..

[159] Lundberg J.O., Weitzberg E. (2022). Nitric Oxide Signaling in Health and Disease. Cell.

[160] Kapil V., Khambata R.S., Jones D.A., Rathod K., Primus C., Massimo G., Fukuto J.M., Ahluwalia A. (2020). The Noncanonical Pathway for In Vivo Nitric Oxide Generation: The Nitrate-Nitrite-Nitric Oxide Pathway. Pharmacol. Rev..

[161] Lara J., Ashor A.W., Oggioni C., Ahluwalia A., Mathers J.C., Siervo M. (2016). Effects of inorganic nitrate and beetroot supplementation on endothelial function: A systematic review and meta-analysis. Eur. J. Nutr..

[162] Burleigh M., Liddle L., Muggeridge D.J., Monaghan C., Sculthorpe N., Butcher J., Henriquez F., Easton C. (2019). Dietary nitrate supplementation alters the oral microbiome but does not improve the vascular responses to an acute nitrate dose. Nitric Oxide.

[163] Vanhatalo A., Blackwell J.R., L’Heureux J.E., Williams D.W., Smith A., van der Giezen M., Winyard P.G., Kelly J., Jones A.M. (2018). Nitrate-responsive oral microbiome modulates nitric oxide homeostasis and blood pressure in humans. Free Radic. Biol. Med..

[164] Capper T.E., Siervo M., Clifford T., Taylor G., Iqbal W., West D., Stevenson E.J. (2022). Pharmacokinetic profile of incremental oral doses of dietary nitrate in young and older adults: A crossover randomized clinical trial. J. Nutr..

[165] Kapil V., Rathod K.S., Khambata R.S., Bahra M., Velmurugan S., Purba A., Watson D.S., Barnes M.R., Wade W.G., Ahluwalia A. (2018). Sex differences in the nitrate-nitrite-NO^•^ pathway: Role of oral nitrate-reducing bacteria. Free Radic. Biol. Med..

[166] Lundberg J.O., Gladwin M.T., Weitzberg E. (2015). Strategies to increase nitric oxide signalling in cardiovascular disease. Nat. Rev. Drug Discov..

[167] Bondonno C.P., Blekkenhorst L.C., Liu A.H., Bondonno N.P., Ward N.C., Croft K.D., Hodgson J.M. (2018). Vegetable-derived bioactive nitrate and cardiovascular health. Mol. Asp. Med..

[168] Sanlier N., Ejder Z.B., Yalcinkaya I. (2026). Health effects of red beetroot as a functional food: Healing from the root. J. Food Sci..

[169] Esatbeyoglu T., Wagner A.E., Motafakkerazad R., Nakajima Y., Matsugo S., Rimbach G. (2014). Free radical scavenging and antioxidant activity of betanin: Electron spin resonance spectroscopy studies and studies in cultured cells. Food Chem. Toxicol..

[170] Kim S.L., Choi H.S., Ko Y.C., Yun B.S., Lee D.S. (2021). 5-Hydroxymaltol derived from beetroot juice through *Lactobacillus* fermentation suppresses inflammatory effect and oxidant stress via regulating NF-κB, MAPKs pathway and NRF2/HO-1 expression. Antioxidants.

[171] Tkaczenko H., Kurhaluk N. (2025). Antioxidant-rich functional foods and exercise: Unlocking metabolic health through Nrf2 and related pathways. Int. J. Mol. Sci..

[172] Moreno-Ley C.M., Osorio-Revilla G., Hernández-Martínez D.M., Ramos-Monroy O.A., Gallardo-Velázquez T. (2021). Anti-Inflammatory Activity of Betalains: A Comprehensive Review. Hum. Nutr. Metab..

[173] Ahmadi H., Nayeri Z., Minuchehr Z., Sabouni F., Mohammadi M. (2020). Betanin purification from red beetroots and evaluation of its anti-oxidant and anti-inflammatory activity on LPS-activated microglial cells. PLoS ONE.

[174] Asgary S., Afshani M.R., Sahebkar A., Keshvari M., Taheri M., Jahanian E., Rafieian-Kopaei M., Malekian F., Sarrafzadegan N. (2016). Improvement of hypertension, endothelial function and systemic inflammation following short-term supplementation with red beet (*Beta vulgaris* L.) juice: A randomized crossover pilot study. J. Hum. Hypertens..

[175] Karimzadeh L., Behrouz V., Sohrab G., Hedayati M., Emami G. (2022). A randomized clinical trial of beetroot juice consumption on inflammatory markers and oxidative stress in patients with type 2 diabetes. J. Food Sci..

[176] Liu H., Huang Y., Huang M., Wang M., Ming Y., Chen W., Chen Y., Tang Z., Jia B. (2023). From nitrate to NO: Potential effects of nitrate-reducing bacteria on systemic health and disease. Eur. J. Med. Res..

[177] Fujii A., Akatsu T., Souno H., Kawano S., Minegishi Y., Ota N. (2025). Enhanced dominance of nitrate-reducing bacteria using a combination of nitrate and erythritol in in vitro cultured oral biofilm. J. Oral Microbiol..

[178] Willmott T., Ormesher L., McBain A.J., Humphreys G.J., Myers J.E., Singh G., Lundberg J.O., Weitzberg E., Nihlen C., Cottrell E.C. (2023). Altered oral nitrate reduction and bacterial profiles in hypertensive women predict blood pressure lowering following acute dietary nitrate supplementation. Hypertension.

[179] du Toit L., Sundqvist M.L., Redondo-Rio A., Brookes Z., Casas-Agustench P., Hickson M., Benavente A., Montagut G., Weitzberg E., Gabaldón T. (2024). The effect of dietary nitrate on the oral microbiome and salivary biomarkers in individuals with high blood pressure. J. Nutr..

[180] Fejes R., Séneca J., Pjevac P., Lutnik M., Weisshaar S., Pilat N., Steiner R., Wagner K.H., Woodman R.J., Bondonno C.P. (2025). Increased nitrate intake from beetroot juice over 4 weeks changes the composition of the oral, but not the intestinal microbiome. Mol. Nutr. Food Res..

[181] Mosele J.I., Macià A., Motilva M.J. (2015). Metabolic and microbial modulation of the large intestine ecosystem by non-absorbed diet phenolic compounds: A review. Molecules.

[182] Sawant S.S., Park H.-Y., Sim E.-Y., Kim H.-S., Choi H.-S. (2025). Microbial fermentation in food: Impact on functional properties and nutritional enhancement—A review of recent developments. Fermentation.

[183] Wang Y., Do T., Marshall L.J., Boesch C. (2023). Effect of two-week red beetroot juice consumption on modulation of gut microbiota in healthy human volunteers—A pilot study. Food Chem..

[184] Ko G.P., Jo H., Kim J., Kim J.S., Boo K.H., Kim C.S. (2024). Enterotype-specific effects of red beetroot (*Beta vulgaris* L.) powder and betanin on human gut microbiota: A preliminary study based on in vitro fecal fermentation model. Life.

[185] Anumudu C.K., Miri T., Onyeaka H. (2024). Multifunctional applications of lactic acid bacteria: Enhancing safety, quality, and nutritional value in foods and fermented beverages. Foods.

[186] Wang Y., Wu J., Lv M., Shao Z., Hungwe M., Wang J., Bai X., Xie J., Wang Y., Geng W. (2021). Metabolism characteristics of lactic acid bacteria and the expanding applications in food industry. Front. Bioeng. Biotechnol..

[187] Calvani R., Giampaoli O., Marini F., Del Chierico F., De Rosa M., Conta G., Sciubba F., Tosato M., Picca A., Ciciarello F. (2024). Beetroot juice intake positively influenced gut microbiota and inflammation but failed to improve functional outcomes in adults with long COVID: A pilot randomized controlled trial. Clin. Nutr..

[188] Khubber S., Marti-Quijal F.J., Tomasevic I., Remize F., Barba F.J. (2022). Lactic acid fermentation as a useful strategy to recover antimicrobial and antioxidant compounds from food and by-products. Curr. Opin. Food Sci..

[189] Foss K., Starowicz M., Kłębukowska L., Sawicki T. (2023). Effect of lactic acid fermentation of red beetroot juice on volatile compounds profile and content. Eur. Food Res. Technol..

[190] Gaur G., Gänzle M.G. (2023). Conversion of (poly)phenolic compounds in food fermentations by lactic acid bacteria: Novel insights into metabolic pathways and functional metabolites. Curr. Res. Food Sci..

[191] LeBlanc J.G., Laiño J.E., del Valle M.J., Vannini V., van Sinderen D., Taranto M.P., de Valdez G.F., de Giori G.S., Sesma F. (2011). B-group vitamin production by lactic acid bacteria—Current knowledge and potential applications. J. Appl. Microbiol..

[192] Keyvan E., Adesemoye E., Champomier-Vergès M.C., Chanséaume-Bussiere E., Mardon J., Nikolovska Nedelkoska D., Palamutoglu R., Russo P., Sarand I., Songre-Ouattara L. (2025). Vitamins formed by microorganisms in fermented foods: Effects on human vitamin status—A systematic narrative review. Front. Nutr..

[193] Clifford T., Howatson G., West D.J., Stevenson E.J. (2017). Beetroot juice is more beneficial than sodium nitrate for attenuating muscle pain after strenuous eccentric-bias exercise. Appl. Physiol. Nutr. Metab..

[194] Jones L., Bailey S.J., Rowland S.N., Alsharif N., Shannon O.M., Clifford T. (2022). The effect of nitrate-rich beetroot juice on markers of exercise-induced muscle damage: A systematic review and meta-analysis of human intervention trials. J. Diet. Suppl..

[195] Webb A.J., Patel N., Loukogeorgakis S., Okorie M., Aboud Z., Misra S., Rashid R., Miall P., Deanfield J., Benjamin N. (2008). Acute blood pressure lowering, vasoprotective, and antiplatelet properties of dietary nitrate via bioconversion to nitrite. Hypertension.

[196] Mirmiran P., Houshialsadat Z., Gaeini Z., Bahadoran Z., Azizi F. (2020). Functional properties of beetroot (*Beta vulgaris*) in management of cardio-metabolic diseases. Nutr. Metab..

[197] Widmer R.J., Lerman A. (2014). Endothelial dysfunction and cardiovascular disease. Glob. Cardiol. Sci. Pract..

[198] Velmurugan S., Gan J.M., Rathod K.S., Khambata R.S., Ghosh S.M., Hartley A., Van Eijl S., Sagi-Kiss V., Chowdhury T.A., Curtis M. (2016). Dietary nitrate improves vascular function in patients with hypercholesterolemia: A randomized, double-blind, placebo-controlled study. Am. J. Clin. Nutr..

[199] Jones T., Dunn E.L., Macdonald J.H., Kubis H.P., McMahon N., Sandoo A. (2019). The effects of beetroot juice on blood pressure, microvascular function and large-vessel endothelial function: A randomized, double-blind, placebo-controlled pilot study in healthy older adults. Nutrients.

[200] Vanhatalo A., L’Heureux J.E., Black M.I., Blackwell J.R., Aizawa K., Thompson C., Williams D.W., van der Giezen M., Winyard P.G., Jones A.M. (2025). Ageing modifies the oral microbiome, nitric oxide bioavailability and vascular responses to dietary nitrate supplementation. Free Radic. Biol. Med..

[201] da Silva L.S.L., Gonçalves L.D.S., Junior M.F.T., Sousa Y.B.A., Rebelo M.A., Guimaraes C.S., Tanus-Santos J.E., Bueno Junior C.R., Benjamim J. (2026). Beetroot juice enhances nitrate metabolism and endothelial function but not cardiovascular or strength performance in bodybuilders with a history of anabolic-androgenic steroid abuse: A crossover trial. Antioxidants.

[202] Chang P.Y., Hafiz M.S., Boesch C. (2018). Beetroot juice attenuates glycaemic response in healthy volunteers. Proc. Nutr. Soc..

[203] Aliahmadi M., Amiri F., Bahrami L.S., Hosseini A.F., Abiri B., Vafa M. (2021). Effects of raw red beetroot consumption on metabolic markers and cognitive function in type 2 diabetes patients. J. Diabetes Metab. Disord..

[204] Beals J.W., Binns S.E., Davis J.L., Giordano G.R., Klochak A.L., Paris H.L., Schweder M.M., Peltonen G.L., Scalzo R.L., Bell C. (2017). Concurrent beet juice and carbohydrate ingestion: Influence on glucose tolerance in obese and nonobese adults. J. Nutr. Metab..

[205] Abedimanesh N., Asghari S., Mohammadnejad K., Daneshvar Z., Rahmani S., Shokoohi S., Farzaneh A.H., Hosseini S.H., Jafari Anarkooli I., Noubarani M. (2021). The anti-diabetic effects of betanin in streptozotocin-induced diabetic rats through modulating AMPK/SIRT1/NF-κB signaling pathway. Nutr. Metab..

[206] Allegra M., Tesoriere L., Livrea M.A. (2007). Betanin inhibits the myeloperoxidase/nitrite-induced oxidation of human low-density lipoproteins. Free Radic. Res..

[207] Wroblewska M., Juskiewicz J., Wiczkowski W. (2011). Physiological properties of beetroot crisps applied in standard and dyslipidaemic diets of rats. Lipids Health Dis..

[208] Sakihama Y., Maeda M., Hashimoto M., Tahara S., Hashidoko Y. (2012). Beetroot betalain inhibits peroxynitrite-mediated tyrosine nitration and DNA strand cleavage. Free Radic. Res..

[209] Amirpoor A., Zavar R., Amerizadeh A., Asgary S., Moradi S., Farzaei M.H., Masoumi G., Sadeghi M. (2022). Effect of beetroot consumption on serum lipid profile: A systematic review and meta-analysis. Curr. Probl. Cardiol..

[210] Afzali N., Shokri Y., Zare M., Ashtary-Larky D. (2020). Effect of *Beta vulgaris* extract on liver enzymes and lipid profile in patients with non-alcoholic fatty liver disease: A randomized controlled trial. J. Complement. Integr. Med..

[211] Domínguez R., Maté-Muñoz J.L., Cuenca E., García-Fernández P., Mata-Ordoñez F., Lozano-Estevan M.C., Veiga-Herreros P., da Silva S.F., Garnacho-Castaño M.V. (2018). Effects of beetroot juice supplementation on intermittent high-intensity exercise efforts. J. Int. Soc. Sports Nutr..

[212] Zoughaib W.S., Fry M.J., Singhal A., Coggan A.R. (2024). Beetroot juice supplementation and exercise performance: Is there more to the story than just nitrate?. Front. Nutr..

[213] Jones A.M. (2014). Influence of dietary nitrate on the physiological determinants of exercise performance: A critical review. Appl. Physiol. Nutr. Metab..

[214] Lee E., Park H.Y., Sun Y., Choi J.H., Woo S., Cho S., Kim S., Zheng Y., Kim S.W., Lim K. (2026). Beetroot juice and exercise for clinical health and athletic performance: A narrative review. Nutrients.

[215] Wylie L.J., Bailey S.J., Kelly J., Blackwell J.R., Vanhatalo A., Jones A.M. (2016). Influence of beetroot juice supplementation on intermittent exercise performance. Eur. J. Appl. Physiol..

[216] Thompson C., Wylie L.J., Blackwell J.R., Fulford J., Black M.I., Kelly J., McDonagh S.T., Carter J., Bailey S.J., Vanhatalo A. (2017). Influence of dietary nitrate supplementation on physiological and muscle metabolic adaptations to sprint interval training. J. Appl. Physiol..

[217] McDonagh S.T., Vanhatalo A., Fulford J., Wylie L.J., Bailey S.J., Jones A.M. (2016). Dietary nitrate supplementation attenuates the reduction in exercise tolerance following blood donation. Am. J. Physiol. Heart Circ. Physiol..

[218] Delleli S., Ouergui I., Messaoudi H., Trabelsi K., Glenn J.M., Ammar A., Chtourou H. (2023). Does beetroot supplementation improve performance in combat sports athletes? A systematic review of randomized controlled trials. Nutrients.

[219] Meng F., Liu Y., Qiu B., Li J. (2025). Does nitrate supplementation improve muscle strength, power, and sprint performance in females? A systematic review and meta-analysis. Life.

[220] Clifford T., Bell O., West D.J., Howatson G., Stevenson E.J. (2016). The effects of beetroot juice supplementation on indices of muscle damage following eccentric exercise. Eur. J. Appl. Physiol..

[221] Clifford T., Berntzen B., Davison G.W., West D.J., Howatson G., Stevenson E.J. (2016). Effects of beetroot juice on recovery of muscle function and performance between bouts of repeated sprint exercise. Nutrients.

[222] Gamonales J.M., Rojas-Valverde D., Muñoz-Jiménez J., Serrano-Moreno W., Ibáñez S.J. (2022). Effectiveness of nitrate intake on recovery from exercise-related fatigue: A systematic review. Int. J. Environ. Res. Public Health.

[223] Tian C., Jiang Q., Han M., Guo L., Huang R., Zhao L., Mao S. (2025). Effects of beetroot juice on physical performance in professional athletes and healthy individuals: An umbrella review. Nutrients.

[224] Alvares T.S., Oliveira G.V., Volino-Souza M., Conte-Junior C.A., Murias J.M. (2022). Effect of dietary nitrate ingestion on muscular performance: A systematic review and meta-analysis of randomized controlled trials. Crit. Rev. Food Sci. Nutr..

[225] Bian C., Ji L., Xu W., Dong S., Pan N. (2024). Research progress on bioactive substances of beets and their functions. Molecules.

[226] Lechner J.F., Stoner G.D. (2019). Red beetroot and betalains as cancer chemopreventative agents. Molecules.

[227] Saber A., Abedimanesh N., Somi M.H., Khosroushahi A.Y., Moradi S. (2023). Anticancer properties of red beetroot hydro-alcoholic extract and its main constituent; betanin on colorectal cancer cell lines. BMC Complement. Med. Ther..

[228] Nowacki L., Vigneron P., Rotellini L., Cazzola H., Merlier F., Prost E., Ralanairina R., Gadonna J.P., Rossi C., Vayssade M. (2015). Betanin-enriched red beetroot (*Beta vulgaris* L.) extract induces apoptosis and autophagic cell death in MCF-7 cells. Phytother. Res..

[229] Klewicka E., Nowak A., Zduńczyk Z., Juśkiewicz J., Cukrowska B. (2012). Protective effect of lactofermented red beetroot juice against aberrant crypt foci formation, genotoxicity of fecal water and oxidative stress induced by 2-amino-1-methyl-6-phenylimidazo[4,5-b]pyridine in rats model. Environ. Toxicol. Pharmacol..

[230] Sawicki T., Topolska J., Romaszko E., Wiczkowski W. (2018). Profile and Content of Betalains in Plasma and Urine of Volunteers after Long-Term Exposure to Fermented Red Beet Juice. J. Agric. Food Chem..

[231] Wiczkowski W., Romaszko E., Szawara-Nowak D., Piskula M.K. (2018). The impact of the matrix of red beet products and interindividual variability on betacyanins bioavailability in humans. Food Res. Int..

[232] Daliri E.B.-M., Balnionytė T., Stankevičiūtė J., Lastauskienė E., Meškys R., Burokas A. (2023). High temperature lacto-fermentation improves antioxidant and antidiabetic potentials of Lithuanian red beetroot. LWT.

[233] Kurhaluk N., Kołodziejska R., Buyun L., Kamiński P., Tkaczenko H. (2026). Dietary Nitrate-Rich Vegetables as Natural Modulators of Health: Mechanisms and Benefits in Ageing Populations. Int. J. Mol. Sci..

[234] Vanhatalo A., L’Heureux J.E., Kelly J., Blackwell J.R., Wylie L.J., Fulford J., Winyard P.G., Williams D.W., van der Giezen M., Jones A.M. (2021). Network analysis of nitrate-sensitive oral microbiome reveals interactions with cognitive function and cardiovascular health across dietary interventions. Redox Biol..

[235] Mann E.R., Lam Y.K., Uhlig H.H. (2024). Short-chain fatty acids: Linking diet, the microbiome and immunity. Nat. Rev. Immunol..

[236] Parada Venegas D., De la Fuente M.K., Landskron G., González M.J., Quera R., Dijkstra G., Harmsen H.J.M., Faber K.N., Hermoso M.A. (2019). Short chain fatty acids (SCFAs)-mediated gut epithelial and immune regulation and its relevance for inflammatory bowel diseases. Front. Immunol..

[237] Marzetti E., Coelho-Júnior H.J., Calvani R., Girolimetti G., Di Corato R., Ciciarello F., Galluzzo V., Di Mario C., Tolusso B., Santoro L. (2025). Mitochondria-Derived Vesicles and Inflammatory Profiles of Adults with Long COVID Supplemented with Red Beetroot Juice: Secondary Analysis of a Randomized Controlled Trial. Int. J. Mol. Sci..

[238] Bond V., Curry B.H., Adams R.G., Asadi M.S., Millis R.M., Haddad G.E. (2013). Effects of Dietary Nitrates on Systemic and Cerebrovascular Hemodynamics. Cardiol. Res. Pract..

[239] Wightman E.L., Haskell-Ramsay C.F., Thompson K.G., Blackwell J.R., Winyard P.G., Forster J., Jones A.M., Kennedy D.O. (2015). Dietary Nitrate Modulates Cerebral Blood Flow Parameters and Cognitive Performance in Humans: A Double-Blind, Placebo-Controlled, Crossover Investigation. Physiol. Behav..

[240] Presley T.D., Morgan A.R., Bechtold E., Clodfelter W., Dove R.W., Jennings J.M., Kraft R.A., King S.B., Laurienti P.J., Rejeski W.J. (2011). Acute Effect of a High Nitrate Diet on Brain Perfusion in Older Adults. Nitric Oxide.

[241] Gilchrist M., Winyard P.G., Fulford J., Anning C., Shore A.C., Benjamin N. (2014). Dietary Nitrate Supplementation Improves Reaction Time in Type 2 Diabetes: Development and Application of a Novel Nitrate-Depleted Beetroot Juice Placebo. Nitric Oxide.

[242] Kelly J., Fulford J., Vanhatalo A., Blackwell J.R., French O., Bailey S.J., Gilchrist M., Winyard P.G., Jones A.M. (2013). Effects of Short-Term Dietary Nitrate Supplementation on Blood Pressure, O2 Uptake Kinetics, and Muscle and Cognitive Function in Older Adults. Am. J. Physiol. Regul. Integr. Comp. Physiol..

[243] Babateen A.M., Shannon O.M., O’Brien G.M., Okello E., Smith E., Olgacer D., Koehl C., Fostier W., Wightman E., Kennedy D. (2022). Incremental Doses of Nitrate-Rich Beetroot Juice Do Not Modify Cognitive Function and Cerebral Blood Flow in Overweight and Obese Older Adults: A 13-Week Pilot Randomised Clinical Trial. Nutrients.

[244] Matysek M., Kowalczuk-Vasilev E., Szalak R., Baranowska-Wójcik E., Arciszewski M.B., Szwajgier D. (2022). Can Bioactive Compounds in Beetroot/Carrot Juice Have a Neuroprotective Effect? Morphological Studies of Neurons Immunoreactive to Calretinin of the Rat Hippocampus after Exposure to Cadmium. Foods.

[245] Szymański J., Szwajgier D., Baranowska-Wójcik E. (2023). The Role of Beetroot Ingredients in the Prevention of Alzheimer’s Disease. Appl. Sci..

[246] Oliveira Filho J.G., Bertolo M.R.V., Rodrigues M.Á.V., Silva G.D.C., Mendonça G.M.N., Bogusz Junior S., Ferreira M.D., Egea M.B. (2022). Recent Advances in the Development of Smart, Active, and Bioactive Biodegradable Biopolymer-Based Films Containing Betalains. Food Chem..

[247] Abedi-Firoozjah R., Parandi E., Heydari M., Kolahdouz-Nasiri A., Bahraminejad M., Mohammadi R., Rouhi M., Garavand F. (2023). Betalains as Promising Natural Colorants in Smart/Active Food Packaging. Food Chem..

[248] Gawlik-Dziki U., Świeca M., Sułkowski M., Dziki D., Baraniak B., Czyż J. (2013). Antioxidant and Anticancer Activities of *Chenopodium quinoa* Leaves Extracts—In Vitro Study. Food Chem. Toxicol..

[249] Kumar S., Brooks M.S.-L. (2018). Use of Red Beet (*Beta vulgaris* L.) for Antimicrobial Applications—A Critical Review. Food Bioprocess Technol..

[250] Ananda S., Pangsibidang R., Wahyuningtias D. (2026). Antibacterial Activity of Beet Extract (*Beta vulgaris* L.) against Gram-Positive Bacteria: Systematic Literature Review. MEDALION J. Med. Res. Nurs. Health Midwife Particip..

[251] Gong S., Jiao C., Guo L., Jiang Y. (2023). Beetroot (*Beta vulgaris*) Extract against *Salmonella Typhimurium* via Apoptosis-Like Death and Its Potential for Application in Cooked Pork. Int. J. Mol. Sci..

[252] Gong S., Jiao C., Liu B., Qu W., Guo L., Jiang Y. (2023). Beetroot (*Beta vulgaris*) Extract Exerts an Antibacterial Effect by Inducing Apoptosis-Like Death in *Bacillus cereus*. J. Funct. Foods.

[253] Thompson K.G., Turner L., Prichard J., Dodd F., Kennedy D.O., Haskell C., Blackwell J.R., Jones A.M. (2014). Influence of Dietary Nitrate Supplementation on Physiological and Cognitive Responses to Incremental Cycle Exercise. Respir. Physiol. Neurobiol..

[254] Ashor A.W., Lara J., Siervo M. (2017). Medium-Term Effects of Dietary Nitrate Supplementation on Systolic and Diastolic Blood Pressure in Adults: A Systematic Review and Meta-Analysis. J. Hypertens..

[255] Norouzzadeh M., Hasan Rashedi M., Payandeh N., Mirdar Harijani A., Shahinfar H. (2024). The effects of dietary nitrate on blood pressure and vascular health: An umbrella review and updated meta-analysis and meta-regression. J. Funct. Foods.

[256] Lee E.J., An D., Nguyen C.T., Patil B.S., Kim J., Yoo K.S. (2014). Betalain and Betaine Composition of Greenhouse- or Field-Produced Beetroot (*Beta vulgaris* L.) and Inhibition of HepG2 Cell Proliferation. J. Agric. Food Chem..

[257] Surma M., Sawicki T., Piskuła M., Wiczkowski W. (2023). Relationship between the Consumption of Fermented Red Beetroot Juice and Levels of Perfluoroalkyl Substances in the Human Body’s Fluids and Blood Parameters. Int. J. Mol. Sci..

[258] Tan X., Cui F., Wang D., Lv X., Li X., Li J. (2024). Fermented Vegetables: Health Benefits, Defects, and Current Technological Solutions. Foods.

[259] Azeredo H.M.C. (2009). Betalains: Properties, Sources, Applications, and Stability—A Review. J. Food Sci..

[260] Gardini F., Özogul Y., Suzzi G., Tabanelli G., Özogul F. (2016). Technological Factors Affecting Biogenic Amine Content in Foods: A Review. Front. Microbiol..

[261] Barbieri F., Montanari C., Gardini F., Tabanelli G. (2019). Biogenic Amine Production by Lactic Acid Bacteria: A Review. Foods.

[262] Habermeyer M., Roth A., Guth S., Diel P., Engel K.H., Epe B., Fürst P., Heinz V., Humpf H.U., Joost H.G. (2015). Nitrate and Nitrite in the Diet: How to Assess Their Benefit and Risk for Human Health. Mol. Nutr. Food Res..

[263] Walters C.L. (1992). Reactions of Nitrate and Nitrite in Foods with Special Reference to the Determination of N-Nitroso Compounds. Food Addit. Contam..

[264] Gangolli S.D., van den Brandt P.A., Feron V.J., Janzowsky C., Koeman J.H., Speijers G.J., Spiegelhalder B., Walker R., Wisnok J.S. (1994). Nitrate, Nitrite and N-Nitroso Compounds. Eur. J. Pharmacol..

[265] Bowles E.F., Burleigh M., Mira A., Van Breda S.G.J., Weitzberg E., Rosier B.T. (2025). Nitrate: “The Source Makes the Poison”. Crit. Rev. Food Sci. Nutr..

[266] Favari C., Rinaldi de Alvarenga J.F., Sánchez-Martínez L., Tosi N., Mignogna C., Cremonini E., Manach C., Bresciani L., Del Rio D., Mena P. (2024). Factors Driving the Inter-Individual Variability in the Metabolism and Bioavailability of (Poly)phenolic Metabolites: A Systematic Review of Human Studies. Redox Biol..

[267] Shi H., An F., Lin H., Li M., Wu J., Wu R. (2022). Advances in Fermented Foods Revealed by Multi-Omics: A New Direction toward Precisely Clarifying the Roles of Microorganisms. Front. Microbiol..

[268] Wen L., Yang L., Chen C., Li J., Fu J., Liu G., Kan Q., Ho C.T., Huang Q., Lan Y. (2024). Applications of Multi-Omics Techniques to Unravel the Fermentation Process and the Flavor Formation Mechanism in Fermented Foods. Crit. Rev. Food Sci. Nutr..

[269] Brennan L., de Roos B. (2023). Role of Metabolomics in the Delivery of Precision Nutrition. Redox Biol..

[270] Gao Y., Hou L., Gao J., Li D., Tian Z., Fan B., Wang F., Li S. (2021). Metabolomics Approaches for the Comprehensive Evaluation of Fermented Foods: A Review. Foods.

[271] Humblot C., Alvanoudi P., Alves E., Assunçao R., Belovic M., Bulmus-Tuccar T., Chassard C., Derrien M., Karagöz M.F., Karakaya S. (2025). A Scoping Review of the Health Effects of Fermented Foods in Specific Human Populations and Their Potential Role in Precision Nutrition: Current Knowledge and Gaps. Front. Nutr..

[272] Caffrey E.B., Sonnenburg J.L., Devkota S. (2024). Our Extended Microbiome: The Human-Relevant Metabolites and Biology of Fermented Foods. Cell Metab..

